# The Identification of Transcriptomic and Phytohormonal Biomarkers for Monitoring Drought and Evaluating the Potential of Acibenzolar-S-Methyl Root Application to Prime Two Apple Rootstock Genotypes for Drought Resistance

**DOI:** 10.3390/ijms26146986

**Published:** 2025-07-21

**Authors:** Kirstin V. Wurms, Tony Reglinski, Erik H. A. Rikkerink, Nick Gould, Catrin S. Günther, Janine M. Cooney, Poppy Buissink, Annette Ah Chee, Christina B. Fehlmann, Dwayne J. A. Jensen, Duncan Hedderley

**Affiliations:** 1 Ruakura Research Centre, Plant & Food Research Group, Bioeconomy Science Institute, Hamilton 3214, New Zealand; tony.reglinski@plantandfood.co.nz (T.R.); catrin.guenther@plantandfood.co.nz (C.S.G.); janine.cooney@plantandfood.co.nz (J.M.C.); poppy.buissink@plantandfood.co.nz (P.B.); annette.ahchee@plantandfood.co.nz (A.A.C.); christina.fehlmann@plantandfood.co.nz (C.B.F.); dwayne.jensen@plantandfood.co.nz (D.J.A.J.); 2 Mount Albert Research Centre, Plant & Food Research Group, Bioeconomy Science Institute, Auckland 1025, New Zealand; erik.rikkerink@plantandfood.co.nz; 3 Te Puke Research Centre, Plant & Food Research Group, Bioeconomy Science Institute, Te Puke 3182, New Zealand; nick.gould@plantandfood.co.nz; 4 Palmerston North Research Centre, Plant & Food Research Group, Bioeconomy Science Institute, Palmerston North 4410, New Zealand; duncan.hedderley@plantandfood.co.nz

**Keywords:** ABA metabolism, abiotic stress, *Malus domestica*, plant hormones, water stress

## Abstract

Droughts are predicted to intensify with climate change, posing a serious threat to global crop production. Increasing drought tolerance in plants requires an understanding of the underlying mechanisms. This study measured the physiological, phytohormonal and transcriptomic responses to drought in two apple rootstocks to identify drought ‘biomarkers’ and investigated whether the application of acibenzolar-S-methyl (ASM) to the roots could enhance drought tolerance. Two potted-plant trials were conducted on dwarfing (M9) and semi-dwarfing (CG202) apple rootstocks. In both trials, the response patterns in the roots and leaves were compared between irrigated and non-irrigated plants over a 14-day period. In trial 2, ASM was applied 14 days before and immediately before withdrawing irrigation. Drought induced significant decreases in transpiration, photosynthesis and stomatal conductance in both trials. This was accompanied by the accumulation of abscisic acid (ABA) metabolites and the upregulation of ABA pathway transcripts (CYP707A1/A2 and NCED3), a decrease in 12-oxophytodienoic acid (cis-OPDA) and the downregulation of ABA receptor genes (PYL4). The responses to drought were greater in the roots than the leaves, broadly similar across both rootstocks, but differed in strength and timing between the rootstocks. The application of ASM to the roots did not significantly affect the responsiveness to drought in either rootstock. The identified phytohormonal and transcriptomic biomarkers require further validation across a broader range of genotypes.

## 1. Introduction

Drought is one of the most impactful abiotic factors affecting yield in agricultural and horticultural systems [1,2,3,4]. For example, Kim et al. [5] analysed historical drought impact and global production data and showed an average yield reduction for annual crops in a single drought period of 8% for wheat, 7% for maize, 3% for rice and 7% for soy. The yield losses tend to be greater in slower-growing perennials. For instance, apple trees experienced a 26% yield reduction in a single drought season [6]. Water deficits can also impair flower initiation, shoot length and branching, leading to decreased yields in subsequent growing seasons [7]. With climate change, the frequency, intensity, duration and geographic spread of drought periods are predicted to increase globally [5]. Furthermore, agriculture and horticulture are the biggest consumers of freshwater [3,8]. Global demands for food (hence water) are increasing, placing even more pressure on a limited resource. Future crop yields and worldwide food security are at risk if we cannot address this issue.

Plants respond to drought through physical and chemical changes mediated by their transcriptome. Gene responses to drought have been intensely studied [3,4,9,10], with numerous reviews showing that key components include genes of the abscisic acid (ABA) pathway [11,12,13]. ABA is the primary phytohormone involved in responses to abiotic stresses such as water extremes (drought, flooding), temperature stress (both hot and cold) and excessive salinity [11,12,14,15]. ABA is a key mitigator of the drought response in plants because it quickly stimulates short-term physiological changes, such as stomatal closure, thus regulating water loss through transpiration. In addition, ABA also regulates longer-term responses, such as dormancy and growth inhibition, by regulating signalling peptides and drought-responsive genes, e.g., genes encoding for membrane and protein stabilisation, antioxidant activity and osmolyte biosynthesis [12,13,16]. While considerable progress has been made in understanding phytohormones’ roles in plant responses to abiotic stress, there are still gaps in our knowledge. These gaps include a clear understanding of the complex effects of hormonal crosstalk and multiple stresses; the potential trade-off between induced stress responses and yield; and the effects of innate vs. induced tolerance in different plant varieties. Moreover, the rationale behind most of the cited studies has been to provide an understanding of the molecular responses to water stress, with a view to breeding new plant varieties with improved drought resilience [3]. In contrast, there have been considerably fewer studies, particularly in perennials, that have combined the use of phytohormonal and transcriptional biomarkers to understand how treatments can be applied to established plantings and induce greater tolerance to subsequent drought stress, a process known as priming [17,18,19].

The treatments used to condition plants to improve their stress tolerance include gradual exposure to a stress factor, e.g., controlled water deprivation [20], and/or the exogenous application of compounds (priming agents) such as phytohormones and plant metabolites [21]. Experimental applications of phytohormones, including salicylic acid (SA) [22,23], cytokinins (CKs) [24] and ABA [25], have demonstrated their potential benefits in improving drought resilience. However, there are few commercialised priming agents to mitigate the impacts of drought stress. These are largely restricted to use in annuals, rather than woody perennials, because of the additional biological complexities and time constraints of the slower growth cycles associated with the latter. The impact of severe drought on existing plantings of perennials has more durable and deleterious effects than those on annuals, with the time taken to reach full production after re-planting requiring years. Consequently, the use of priming agents in perennials merits further research. One suitable candidate for such research is acibenzolar-S-methyl (ASM), a commercially available SA analogue that is often used to induce biotic stress resistance. The exogenous application of SA activated stress response molecular pathways and alleviated water stress in watermelon, rice, wheat and barley [26,27,28,29]. SA also triggered the accumulation of ABA in both normal and salinity-stressed tomato plants, which enhanced their osmotic adaptation and improved their photosynthetic pigment contents and growth [30]. Furthermore, the application of ASM has been shown to enhance drought tolerance in creeping bentgrass [31], induce stomatal closure in Japanese radish [32] and stimulate ABA accumulation in kiwifruit [33], which might improve tolerance to osmotic stress.

The exemplar crop chosen for this study was apple. Drought stress can affect apple quality and yield in both the current and subsequent seasons [34,35] and may cause tree death. Grafted apple trees require at least 7 years to reach full production, and breeding of new cultivars takes up to 15 years [36,37], leaving the industry with a significant recovery period should existing plantings suffer severe droughts. Commercial apple plantings comprise scions grafted onto rootstocks, with the rootstocks performing a key role in controlling tree growth and drought tolerance characteristics [38]. Rootstock genotype has been shown to have a major influence on the response of the scion to water stress [39]. Malling 9 rootstock (M9) is a commonly used strong-dwarfing rootstock thought to confer some drought resistance via ABA-mediated control of the stomata [40]. Cornell-Geneva 202 (CG202), a semi-dwarfing rootstock, appears to have a reduced drought tolerance relative to that of M9 [41], but the reverse result has also been observed [42]. The results from most studies on apple rootstocks have been based on empirical physiological data without any matching data on biochemical and genetic biomarkers [40,41,42,43,44,45].

To address these knowledge gaps, the aims of this study were

1. To identify robust phytohormonal and transcriptomic biomarkers associated with physiological drought responses in two apple rootstocks;
2. To evaluate the effect of the application of ASM on these biomarkers and compare the responses induced between rootstocks.

The underlying hypotheses are that phytohormonal and transcriptional markers can reveal the metabolic drought responses in rootstocks and that the SA analogue, ASM, can induce/prime for drought resistance in a woody perennial (apple).

The study reported here adopts a novel approach through the combined use of selected phytohormones and genetic targets, previously associated with stress response and signalling, to provide an initial system-level understanding of the drought responses in apple.

## 2. Results

### 2.1. Gas Exchange and Water Loss

Two trials were carried out, with trial 2 undertaken to validate the findings from trial 1 and to build further on the learnings by using a priming agent (ASM). Pot weight was used to monitor the relative reduction in the soil’s water content in the non-irrigated (dry) pots over the duration of each experiment. The pot weight for the irrigated plants was maintained throughout the course of the experiment for both trials. In comparison, the dry plants lost weight throughout the trial period because of water loss, resulting in increasing drought stress over the 14 days (Table 1 and Table 2). The loss of pot weight in the dry plants was similar between rootstocks and trials, suggesting similar water stress levels applied. However, the average temperature over the 14-day period differed between trial 1 (21.7 °C) and trial 2 (18.1 °C) (Supplementary Figure S1). This difference was particularly evident during days 1–5, with the maximum temperature being up to 8 °C higher in trial 1 and an average temperature of 22.6 °C in trial 1, compared with 17.1 °C in trial 2.

**Figure 1:**
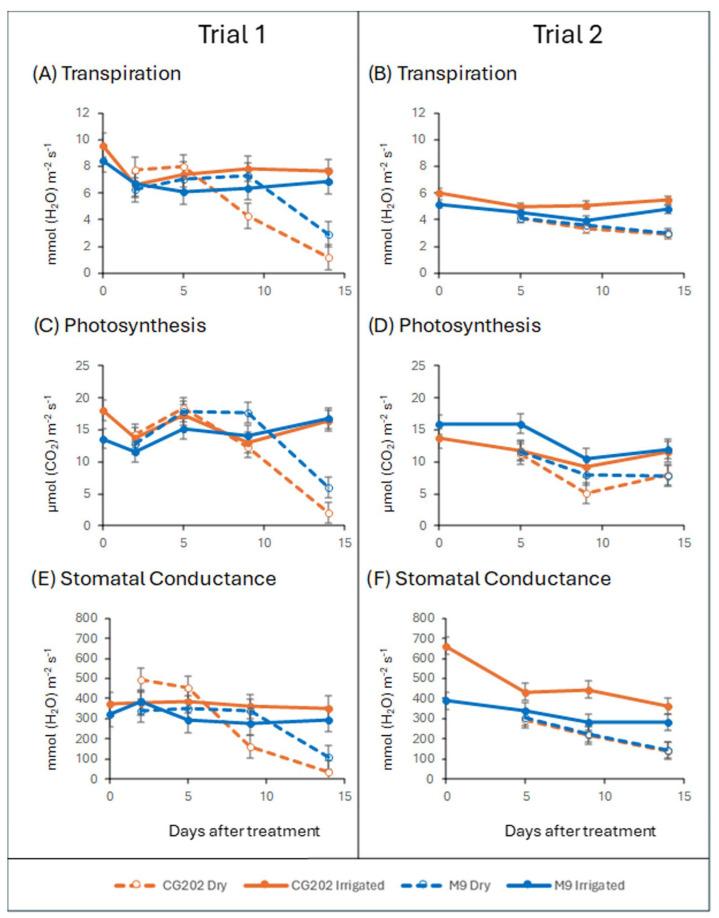
Transpiration rate (**A**,**B**), photosynthesis rate (**C**,**D**) and stomatal conductance (**E**,**F**) in CG202 and M9 *Malus domestica* rootstocks after 2 (trial 1 only), 5, 9 and 14 days without water (dry). Data for trial 1 given in (**A**,**C**,**E**) and data from trial 2 given in (**B**,**D**,**F**). Irrigated control plants received a total volume of 1.8 L water/day via drippers. There were five replicate plants per sampling time, treatment and rootstock. Mean ± standard error bars from the ANOVA shown; the details of significant differences for each treatment against the controls at each time point are given in the main text.

Water deprivation induced a reduction in transpiration in both the CG202 and M9 rootstocks, with the reduction occurring earlier in the former genotype (Figure 1A,B). In trial 1, the transpiration for CG202 was significantly lower in the dry (non-irrigated) plants than that in the irrigated control plants on days 9 (*p* = 0.010) and 14 (*p* < 0.001), whereas for M9, the difference was significant only at day 14 (*p* < 0.01). Similarly, in trial 2, the transpiration in CG202 was significantly lower on days 5 (*p* = 0.047), 9 (*p* < 0.001) and 14 (*p* < 0.001) in the dry plants compared with that for the irrigated control plants, whereas in M9, the difference was significant only at day 14 (*p* < 0.001), but the magnitude of the transpiration changes was smaller in trial 2 vs. trial 1 (Figure 1A,B).

Photosynthesis measurements show an effect of the dry treatment over time for both rootstocks in both trials. In trial 1, the plants under the dry treatment showed a significantly reduced photosynthesis rate on day 14 compared to that under the irrigated treatment (*p* < 0.001 for both rootstocks). In trial 2, photosynthesis was reduced after 9 days of treatment compared to that under the irrigated treatment in the CG202 rootstock (*p* = 0.064). In the M9 rootstock, there was a reduction after 5 (*p* = 0.053) and 14 (*p* = 0.059) days under the dry treatment (Figure 1C,D).

The effect of the dry treatment on the stomatal conductance for both rootstocks in both trials followed the same pattern as that for the transpiration rate. In trial 1, the dry treatment reduced the stomatal conductance compared to that under the irrigated treatment for each rootstock. In the CG202 genotype, transpiration was significantly reduced on days 9 (*p* = 0.024) and 14 (*p* = 0.001) in the dry treatment compared to that under the irrigated treatment. In the M9 rootstock, there was a significant difference only at day 14 (*p* = 0.032). In trial 2, a similar response was observed, with a significant reduction occurring in CG202 on days 5, 9 and 14 (*p* = 0.021, <0.01 and <0.01, respectively) and in M9 on day 14 (*p* = 0.022) (Figure 1E,F)

There was no significant effect of the ASM treatment (only tested in trial 2).

### 2.2. Gene Expression Data—The Selection of Suitable Markers

**Figure 2:**
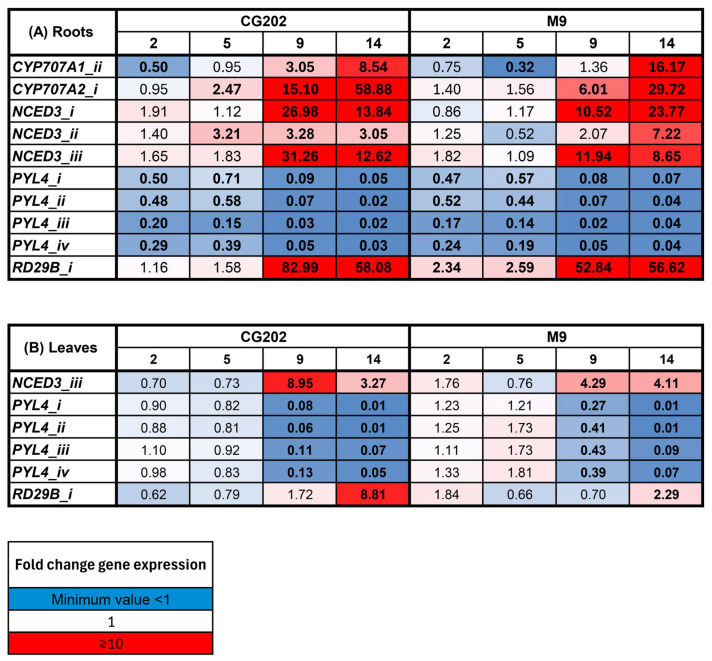
A heat map presenting the gene expression fold change data for the (**A**) root tissue and the (**B**) leaf tissue as induced by drought stress conditions in the *Malus domestica* GG202 and M9 rootstocks after 2, 5, 9 and 14 days without water in trial 1. The control irrigated plants received a total volume of 1.8 L water/day via drippers. There were five replicate plants/sample time/treatment/rootstock. The gene expression was quantified using the PlexSet^®^ NanoString using *CKB4*, *FYPP3* and *GPAT1* as the reference genes. Genes of interest are listed in the left-hand column. Numeric values give the fold changes relative to the values in the irrigated plants, sampled in the same time periods. Red colouration indicates a fold increase, blue represents a fold decrease and white is no fold change relative to the control. Colour intensity is indicative of the degree of change, but all fold increases greater than 10 have the same red colour intensity. Statistically significant differences from the control plants at each time point, as determined through the ANOVA (*p* ≤ 0.05), are indicated in bold typeface.

**Figure 3:**
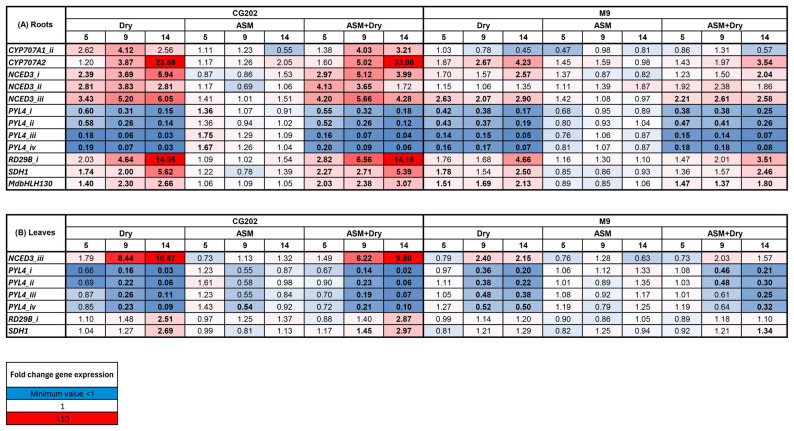
A heat map presenting the fold changes in the gene expression data for the (**A**) roots and (**B**) leaves induced by drought stress conditions and acibenzolar-S-methyl (ASM) application in *Malus domestica* ‘GG202’ and ‘M9’ rootstocks after 5, 9 and 14 days without water in trial 2. ASM (10 mg a.i./plant) was applied as a root drench, both 14 days before the start of experiment and again on day 0, immediately before dripper removal. The control irrigated plants received a total volume of 1.8 L water/day. There were three replicate plants/sample time/treatment/rootstock. The gene expression was quantified using the PlexSet^®^ NanoString using *CKB4*, *FYPP3*, *GPAT1*, *LTL1* and *Protein GRIP* as the reference genes. All fold changes (numerical values in the figure) are relative to the values for irrigated plants at the same time. Red colouration indicates a fold increase, blue represents a fold decrease and white is no fold change relative to the control. Statistically significant differences from the control at each time point, as determined through the ANOVA (*p* ≤ 0.05), are indicated in bold typeface.

The expression of 53 genes of interest (GoIs) and six reference genes (RGs) was measured during the onset of drought stress in the leaf and root tissues. The selection of the GoIs was based on mining a list of candidate genes identified from the literature on both ABA metabolic synthesis and catabolic pathways in apple and model plants and a functional analysis of the genes involved in drought resistance, whilst the RGs were stably expressed apple genes identified in the literature (Supplementary Table S1). The gene expression data presented here are limited to those that showed statistically significant differential expression and/or consistent trends in both trial 1 and trial 2 (for genes measured in both trials). Statistically significant expression was usually considered less than 0.5-fold for downregulated genes and greater than 2-fold for upregulated genes (Figure 2 and Figure 3). All of the other gene expression results can be found in the Supplementary Files (Supplementary Figures S2–S9), and the ANOVAs for trials 1 and 2 can be found in the Supplementary Excel File.

The heat maps show the fold change in the expression (log_2_-transformed count data that have been backtransformed) relative to that in the irrigated control plants at each time point for trials 1 and 2, respectively (Figure 2 and Figure 3). In trial 1, water deficit induced significant upregulation of the NCED3 (ABA synthesis), CYP707A1/A2 (encoding cytochrome P450 monooxygenases involved in ABA catabolism) and RD29B (ABA-inducible) gene families in both rootstock genotypes (Figure 2). The gene upregulation depended on the isoform and was generally greater in the roots than the leaves and greater in CG202 than M9. For example, after 9 days without water, *NCED3_iii* increased 31-fold in the roots and 9-fold in the leaves of the CG202 plants, compared with the 12-fold and 4-fold increases, respectively, in the M9 plants (Figure 2). The expression of *CYP707A2*, which catabolises ABA, was upregulated in the roots only and was 59-fold higher in CG202 and 30-fold higher in M9 after 14 days without water relative to its expression in the irrigated plants. The most highly upregulated gene in the roots of both rootstocks, *RD29B_i*, was upregulated over 80-fold in CG202 and over 50-fold in M9 after 9 days. This can be compared with the more moderate upregulation in the leaves, where the *RD29B_i* expression increased approximately 9-fold and 2-fold in the CG202 and M9 plants, respectively, after 14 days without water (Figure 2). In contrast, water deficit resulted in the significant downregulation of four members of the PYL4 family of ABA receptors (which play a key role in ABA signalling and drought response). All four isoforms were downregulated, with 11- to 50-fold decreases after 9 days without water in the roots of both GC202 and M9 (Figure 2). The decreases in PYL4 occurred earlier in the leaf tissue from GC202 than they did in the leaf tissue from M9, with 14-to 100-fold decreases occurring by 9 days in the non-irrigated GG202 leaves, versus 14 days to reach similar fold decreases in the M9 leaves (Figure 2).

The gene expression patterns for the upregulated gene families (NCED3, CYP707A1/A2 and RD29B) and the downregulated gene family (PYL4) that were seen in trial 1 were also observed in trial 2 (Figure 3). Whilst the patterns of gene expression were consistent between the two trials, the amplitude of the response was much greater in trial 1 than in trial 2. For example, for the most highly upregulated gene, *RD29B_i*, the maximum fold change in the root tissue was 83-fold in CG202 and 57-fold in M9 after 9 and 14 days without water, respectively, in trial 1 (Figure 2). In contrast, the maximum fold increases in *RD29B_i* in the root tissue in trial 2 were 14-fold in CG202 and 5-fold in M9 after 14 days without water (Figure 3). Similarly, the two most downregulated isoforms, *PYL4_i* and *PYL4_ii*, had decreased 100-fold in the leaf tissue from GC202 and M9 after 14 days without water in trial 1, while averaged 25-fold and 5-fold decreases in *PYL4_i* and *PYL4_ii* were seen in the leaves from CG202 and M9, respectively, after 14 days of drought in trial 2 (Figure 2 and Figure 3).

The analysis of the 42 GoIs in trial 1 showed that the transcript abundance of 11 genes (*PYL9_i*, *PYL9_ii*, *PYL9_iii*, *ERD15_i*, *RAP2.4_ii*, *FKBP_ii*, *VHA-B_i*, *VHA-B_ii*, *SAT1*, *HY5* and *SERRATE*) did not differ significantly in expression between the treatments (Figure S2). These genes were replaced with 11 new candidates mined from the literature (*OST1/SnRK 2.6*, *SnRK 2.3*, *SnRK 2.8*, *ATG18_i*, *MADS-box AGL16_i*, *MADS-box AGL16_ii*, *SDH1*, *BES1/BZR2*, *MdbHLH130*, *MdNAC1*, *MdNAC143*) in the revised gene set used for trial 2. Of these newly added genes, only two, *MdbHLH130* and *SDH1*, showed significant upregulation (Figure 3, Figures S3 and S4). *MdbHLH130*, a transcription factor involved in regulating stomatal closure and reactive oxygen species homeostasis, increased up to 3-fold and 2-fold in the roots of CG202 and M9, respectively, after 14 days of drought, but there was no significant increase in the leaves (Figure 3 and Figure S4). *SDH1*, involved in sugar metabolism, increased up to 6-fold and 3-fold in the roots of CG202 and M9, respectively, after 14 days of drought and up to 3-fold in the leaves of GC202, but without a significant increase in the leaves from M9 after 14 days without water (Figure 3).

In trial 2, the application of the SA analogue, ASM, was tested to determine its potential to prime the rootstocks for greater drought resilience. Figure 3 shows that the treatment with ASM did not have a significant effect on the gene expression relative to that in the untreated irrigated and dry controls (F-statistics and *p*-values are given in the Supplementary Excel File). Moreover, the expression profiles for the dry and ASM + dry treatments were the same, indicating no significant effect of ASM on the genes associated with the dry treatment.

Additional information on the transcript counts for selected gene families is presented in the Supplementary Files. Differential expression of the CYP707A1/2 genes was found in the roots and leaves of both rootstock genotypes. In trial 1, the basal level of *CYP707A1_i* at T0 (baseline) was greater in the roots (500-600 transcripts) than that in the leaves (< 5), whilst the opposite was true for *CYP707A1_ii* and *CYP707A2_i*, which were more abundant in the leaves (400–1200) than in the roots (<5) (Figure S5). There was also a differential response to drought in the roots, with the *CYP707A1_ii* and *CYP707A2_i* transcripts increasing 10-fold and 50-fold, respectively, after 14 days, whilst the levels of *CYP707A1_i* were more than halved. The expression of *CYP707A* genes in the leaves fluctuated over time but was not statistically different between the irrigated and dry treatments after 14 days. Similar expression patterns were observed for the *CYP707A* genes in trial 2; however, the basal transcript levels were higher, and the fold differences in the counts between the irrigated and dry treatments were lower (Figure S6).

PYL4 family genes were strongly expressed in the day 0 baseline measurements for both rootstock genotypes (Figure S7). *PYL4_ii* was the most abundant isoform in the roots at T0 in both genotypes, with >2500 transcripts, compared with approximately 1700, 1000 and 700, respectively, for *PYL4_i*, *iii* and *iv*. The respective transcript levels in the leaves at T0 were lower, with 800–1000 copies of *PLY4_i* and *ii* and 70–200 copies of isoforms *iii* and *iv*. The abundance of each *PYL4* isoform reduced significantly in the dry treatment after 14 days, with the *PYL4_i* and *ii* transcript levels falling 100-fold in the leaves and between 14- and 50-fold in the roots (Figure S7). The *PYL4_iii* and *iv* transcript levels reduced 11- to 20-fold in the leaves and 25- to 50-fold in the roots. The response patterns for the *PYL4* genes in the dry treatment in trial 2 mirrored those in trial 1; however, as with the *CYP* genes, the basal transcript levels were generally higher and the fold changes lower in trial 2 than in trial 1 (Figure S8).

All *NCED3* isoforms were expressed at low levels (<50 copies) in the roots and leaves of the CG202 and M9 irrigated control plants in trial 1 (Figure S9). In the droughted plants, the copy numbers for *NCED3_i* and *NCED3_iii* increased to between 300 and 500 in the roots, irrespective of rootstock genotype, whilst *NCED3_ii* levels remained relatively low (Figure S9). The same trends, albeit to a lesser degree, were observed for *NCED3* isoforms in the irrigated and dry treatments in trial 2 (Figure S10). *RD29B_i* was expressed at low levels (<50 copies) in the roots of the irrigated controls but was one of the most strongly upregulated genes in the droughted plants, with its copy number increasing to over 3000 in CG202 and over 1800 in M9 after 14 days in trial 1 (Figure S11).

### 2.3. Phytohormone Analysis

Compounds associated with SA, JA and ABA metabolism, in addition to the plant growth promotor IAA, were targeted for analysis in the root and leaf tissues from the two apple rootstock genotypes. Fifteen compounds were identified in trial 1, and these included bioactive forms of stress-related phytohormones (ABA, SA, JA, JA-Ile) and IAA; hydroxylated catabolites (12-OH-JA, 7-OH-ABA, PA, DPA); glucose conjugates (ABA-GE, SAG); and precursors of SA (benzoic acid) and JA (cis-OPDA, OPC-4) biosynthesis and DHJA. Of these compounds, benzoic acid, 7-OH-ABA and ABA-GE were detected only in the root tissue, and 12 metabolites were common to both plant tissues. The phytohormone compositions of these common metabolites were primarily influenced by the tissue type, with the roots accumulating higher concentrations of jasmonates and IAA (Figure S12), whereas ABA- and SA-derived metabolites were predominant in the leaves from both rootstock genotypes.

To explore the relationships between multiple explanatory variables (genotype, treatment, sampling day) and phytohormone metabolites as the response variables in each plant organ separately, a redundancy analysis (RDA) was performed. Permutation testing of the respective models was significant (both *p* < 0.01) for the roots (F = 14.3) and the leaves (F = 13.6), with genotype explaining the greatest significant (both *p* < 0.001) variation in the phytohormone composition (F_roots_ = 21.4, F_leaves_ = 32.3), followed by treatment for the roots (F = 13.8) and sampling day for the leaves (F = 10.7).

**Figure 4:**
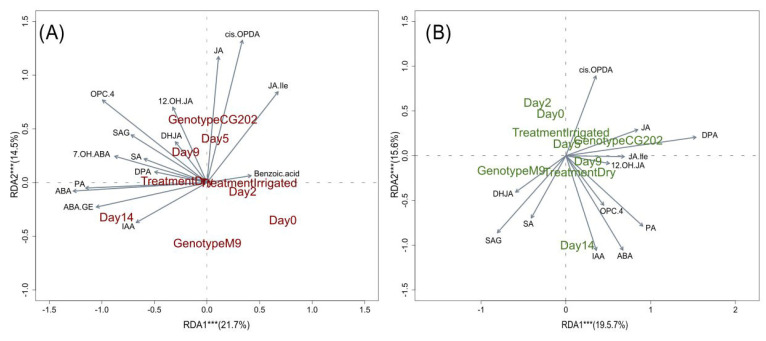
Redundancy analysis (RDA) biplots for (**A**) roots and **(B**) leaves from two apple rootstock genotypes (CG202, M9) with treatment (dry vs. irrigated), genotype and sampling day as the response variables in trial 1. The vectors visualise the variable loadings, and the sample distributions by group are highlighted in brown (**A**) and green (**B**) writing for roots and leaves, respectively. The asterisks indicate that the explained variance is significant at *** α = 0.001. SA: salicylic acid; SAG: salicylic acid glucoside; IAA: indole-3-acetic acid; ABA: abscisic acid; PA: phaseic acid; DPA: dihydrophaseic acid; X7.OH.ABA: 7′-hydroxy-abscisic acid; ABA-GE: abscisic acid glucoside; JA: jasmonic acid; JA-Ile: jasmonic acid–isoleucine; X12OH.JA: 12-hydroxyjasmonic acid; DHJA: dihydrojasmonic acid; OPC.4: 4-(3-oxo-2-(pent-2-en-1-yl)cyclopentyl)octanoic acid; cis.OPDA: 12-oxo-phytodienoic acid.

As a type of constrained principal component analysis, the experimental factors from the RDA that explained the greatest variation in the response data were visualised in sample plots (Figure S13) and loading plots (Figure 4). The F-values were significant (all *p* < 0.003) for RDA1 (F = 38.7)–RDA5 (F = 3.3) and RDA1 (F = 34)–RDA4 (F = 5.2) in the roots and leaves, respectively, indicating that the differences observed between groups were unlikely due to chance. For the roots, the sample plots show that the variation between genotypes was separated on the second component and the variation in the response to treatment on the first component (Figure S13A). Phytohormone responses to drought were already apparent from day 9, with sampling day 14 for the dry plants showing the most prominent separation in both rootstock genotypes. These trends are summarised in Figure 4A, which highlight the relationships of the response variables to each other and to the principal RDA components as vectors. The length of each vector resembles the relative weight of the variable in the component, and its angle indicates the correlation with the other variables. This demonstrates that the variation in phytohormone profiles between genotypes was primarily driven by higher concentrations of JA and cis-OPDA in CG202. The responses to the dry treatment were positively associated with IAA, ABA, ABA-GE and PA. In the leaves, samples were separated by genotype on the first component (Figure S13B) and by treatment on the second component, with responses to drought being apparent from day 14. The variable loadings (Figure 4B) confirmed higher JA and JA-Ile concentrations as characteristic for CG202, in addition to DPA, while M9 showed increased contents of SA, SAG and DHJA as a minor metabolite. The responses to drought were comparable to those observed in the roots and confirmed a rise in IAA, ABA and PA, while cis-OPDA showed the opposite trend.

**Figure 5:**
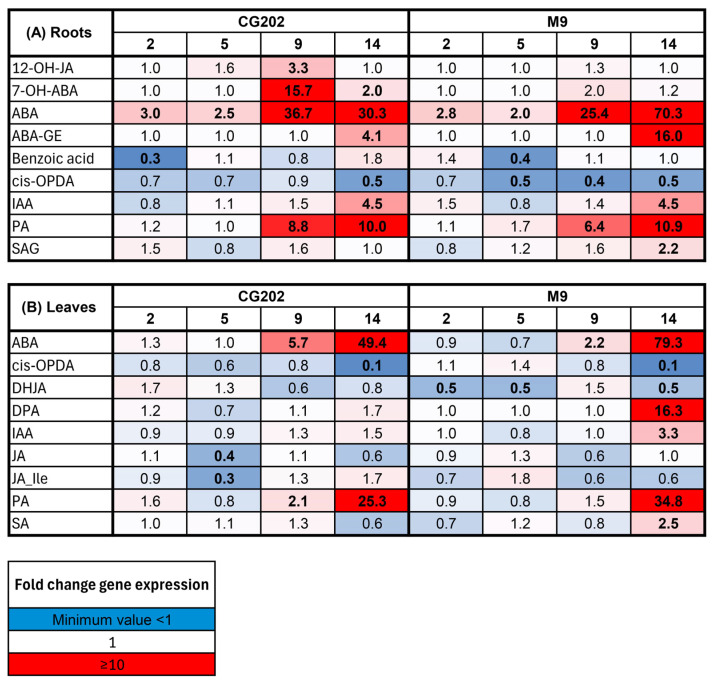
A heat map presenting the fold change data for the metabolite concentrations in the (**A**) roots and (**B**) leaves of the *Malus domestica* GG202 and M9 rootstocks after 0, 2, 5, 9 and 14 days without water in trial 1. All fold changes are relative to the values for irrigated plants at the same time point. Red colouration indicates a fold increase, blue indicates a fold decrease and white is no change relative to the irrigated control. Colour intensity is indicative of the degree of change, but all fold increases greater than 10 have the same red colour intensity. A 2-fold change cutoff was applied for variable selection and bolded data indicate samples with min 2-fold change difference. SA: salicylic acid; SAG: salicylic acid glucoside; IAA: indole-3-acetic acid; ABA: abscisic acid; PA: phaseic acid; DPA: dihydrophaseic acid; 7-OH-ABA: 7′-hydroxy-abscisic acid; ABA-GE: abscisic acid glucoside; JA: jasmonic acid; JA-Ile: jasmonic acid-isoleucine; 12-OH-JA: 12-hydroxyjasmonic acid; DHJA: dihydrojasmonic acid; cis.OPDA: 12-oxo-phytodienoic acid.

To validate the selection of drought-responsive phytohormones and gain an understanding of magnitude, a fold change (FC) analysis at a 2FC cutoff was performed between the dry and irrigated samples (Figure 5). In both genotypes, increased metabolite concentrations were observed for ABA across the entire time course in the roots (Figure 5A) and from day 9 in the leaves (Figure 5B). PA increased at least 2-fold from day 9 in the roots and from day 14 in the leaves, while IAA increased from day 14 in the roots in both rootstocks but only increased in the leaves of M9. The cis-OPDA concentrations decreased 2-fold in the roots in response to drought from day 5 in M9 and from day 14 in CG202 and 9-fold in the leaves from day 14. In both rootstocks, ABA-GE also increased in the roots from day 14, while SAG was 2-fold higher in the roots from M9 and SA was higher in the leaves from M9 at this time point. CG202 responded to drought with increased 7-OH-ABA concentrations in the roots from day 9 and a decreased content of bioactive jasmonates by day 5. In conclusion, ABA and PA were identified as positive and cis-OPDA as negative tissue-independent biomarkers of drought stress and IAA and ABA-GE as positive biomarkers of drought stress in the roots.

**Figure 6:**
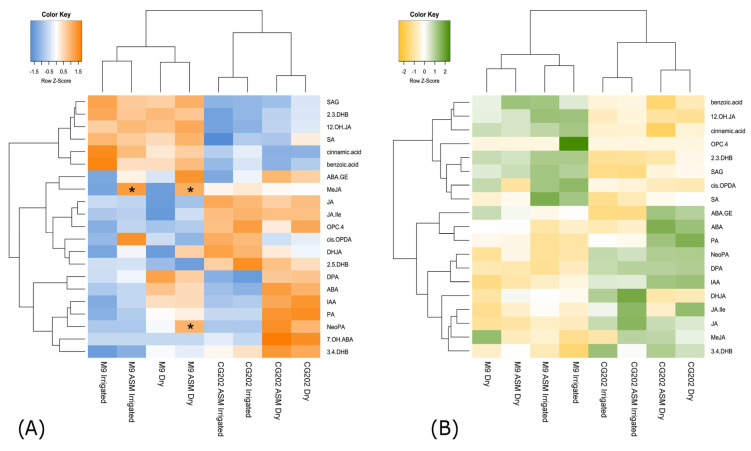
A clustered heat map of the phytohormone concentrations in two apple rootstock genotypes (CG202, M9) across common sampling time points (days 5, 9, 14) in trial 2. (**A**) Roots and (**B**) leaves. Dry: drought-treated; Irrigated: control; ASM: Actigard-treated. Data are scaled by row, with orange (**A**) and green (**B**) hues indicating positive and blue (**A**) and yellow (**B**) hues negative standard (Z-) scores, i.e., relative concentrations. Asterisks indicate statistical differences in ASM-treatment by sample based on pairwise comparisons with α = 0.05. SA: salicylic acid; SAG: salicylic acid glucoside; DHB: dihydroxy benzoic acid; IAA: indole-3-acetic acid; ABA: abscisic acid; PA: phaseic acid; DPA: dihydrophaseic acid; 7-OH-ABA: 7′-hydroxy-abscisic acid; ABA-GE: abscisic acid glucoside; NeoPA: neo phaseic acid; JA: jasmonic acid; JA-Ile: jasmonic acid–isoleucine; 12-OH-JA: 12-hydroxyjasmonic acid; DHJA: dihydrojasmonic acid; OPC-4: 4-(3-oxo-2-(pent-2-en-1-yl)cyclopentyl)octanoic acid; cis-OPDA: 12-oxo-phytodienoic acid; MeJA: methyl jasmonate.

In trial 2, the use of additional standards and an MS detector with higher sensitivity resulted in the quantification of five additional metabolites across all samples, namely bioactive methyl jasmonate (MeJA), the ABA catabolite neophaseic acid (NeoPA) and SA-associated compounds (cinnamic acid, 2.3-DHB and 3.4-DHB). Moreover, ABA-GE and benzoic acid were reliably quantified in the leaves and roots, while 7-OH-ABA and 2.5-DHB were detected only in the roots, resulting in 19 metabolites common to both plant tissues. In line with trial 1, the strongest variation between these phytohormones and associated metabolites was observed between tissue types (Figure S14), with jasmonates and IAA accumulating in the roots and abscisates and salicylates in the leaves of both rootstock genotypes. When the metabolite compositions were compared for each plant tissue separately (Figure 6), the samples clustered into two primary clusters with two subclusters each. For each tissue type, the primary cluster grouped the genotypes, reflecting higher contents of jasmonates and abscisates in CG202 and salicylates in M9 (Figure 6). Differences between the dry and irrigated treatments for each genotype were apparent from the subclusters. Although the absolute concentrations of phytohormones and their metabolites differed between genotypes, the response to drought was consistent between the apple rootstocks. These trends were not affected by the ASM treatment, and treated samples clustered directly with their respective untreated samples (Figure 6). Pairwise comparisons between the respective ASM-treated and untreated samples only highlighted a genotype-dependent significant increase in MeJA in M9 (Figure 6A), which was consistent for both the dry and irrigated roots, and higher concentrations of NeoPA, a minor catabolite, in the dry roots from M9. Thus, the phytohormone profiles appeared to be primarily affected by tissue type, genotype and drought treatment but not ASM priming.

**Figure 7:**
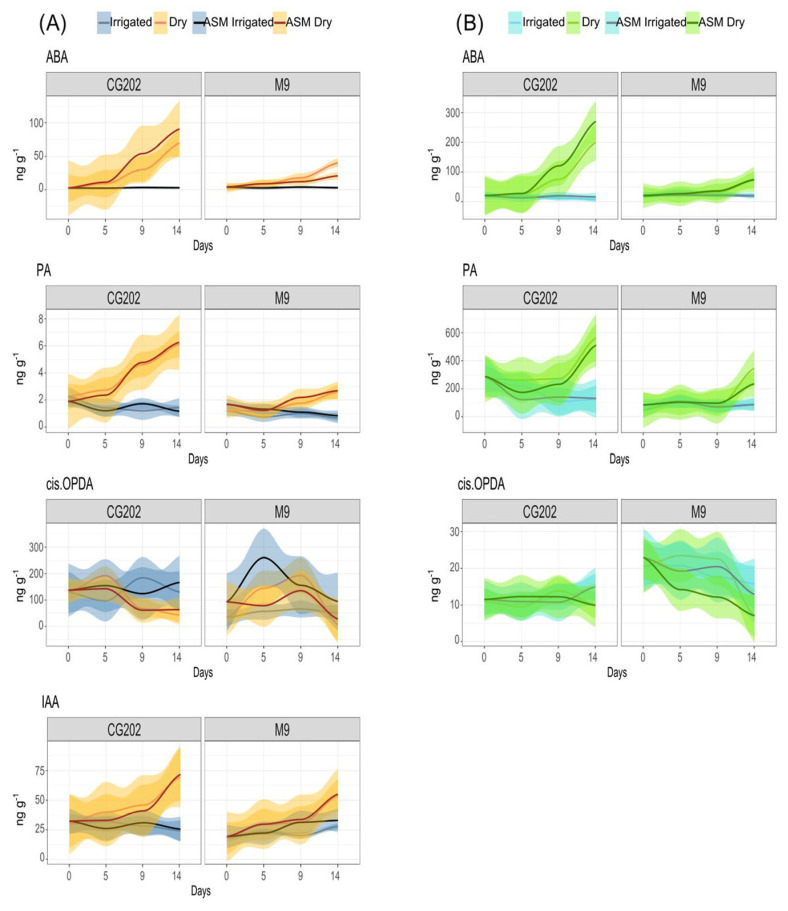
Line graphs showing the mean concentrations of drought-responsive phytohormone metabolites from the (**A**) roots and (**B**) leaves of two apple rootstock genotypes (CG202, M9) in trial 2. Days indicate the time since watering for drought-treated (dry) plants, while the control (irrigated) plants were watered during the entire time course. Concentrations are given per tissue fresh weight and represent the mean of four biological replicates. 95% confidence intervals are visualised using shading.

Since the effect of ASM on the phytohormone responses was considered negligible, only non-primed samples were taken forward for the RDA. The models significantly (*p* < 0.01) explained the metabolite variations for the roots (F = 8.2) and leaves (F = 6.4), which were primarily (*p* < 0.001) driven by genotype (F_roots_ = 20.5, F_leaves_ = 18.4) and treatment (F_roots_ = 10.5, F_leaves_ = 3.1) as explanatory variables. For both tissues, RDA1 and RDA2 were significant (*p* < 0.001), with F_RDA1_ > 19.7 and F_RDA2_ > 7.5. The sample plots (Figure S15) and loading plots (Figure S16) for trial 2 reflected the overall trends observed for trial 1; however, the drought-induced changes in the roots’ metabolite composition became pronounced at a later time point (day 14) compared with that in trial 1 (day 9), and IAA was only elevated in the roots but not in the leaves under dry conditions. The fold change analysis (Figure S17), however, was only significant for ABA and PA at day 14 across genotypes and tissue types and for IAA in the roots, with higher metabolite concentrations measured in the dry plants (Figure 7). In contrast to trial 1, ABA-GE did not show a 2FC in the drought-treated roots. Cis-OPDA concentrations were only positively associated with the irrigated roots across genotypes and the leaves for ‘M9’ at day 14 (Figure S17). In the leaves from CG202, cis-OPDA concentrations showed a decreasing trend, trending towards a 30% decrease by day 14 in trial 2.

## 3. Discussion

This research aimed to establish whether transcriptomic and phytohormonal markers could assess the response to drought in dwarfing (M9) and semi-dwarfing (CG202) apple rootstocks and whether pre-treatment with an SA analogue, ASM, could induce drought resistance. Drought resulted in a significant decrease in transpiration, photosynthesis and stomatal conductance in both rootstocks. The physiological responses occurred earlier in CG202 than those in M9 in both trials, suggesting a quicker response to water stress in CG202. Moreover, there was faster and greater accumulation of ABA metabolites (especially in trial 2) and stronger upregulation of ABA pathway genes (in both trials) in CG202 than in M9. The biochemical and genetic reactions were more intense in the roots than the leaf tissue, with the roots being the primary site of water stress perception. The application of ASM had no discernible effect on the physiological, transcriptomic or biochemical parameters measured. Taken together, these data suggest that statistically significant, differentially accumulated biochemical compounds (ABA, PA, cis-OPDA and IAA) and gene families (CYP707A1/A2, NCED3, RD29B, PYL4, MdbHLH130 and SDH1) showing consistent trends in this study were suitable biomarkers of the drought response and that ASM was not an effective primer—at least within the constraints of this study.

Extreme drought conditions were deliberately simulated to invoke the maximum responses, and accordingly, the gas exchange measurements showed that the dry treatment significantly affected both rootstocks in both trials, leading to a significant reduction in the transpiration rate, photosynthesis and stomatal conductance. Transpiration and stomatal responses generally occurred earlier in CG202 (after 5–9 days without water) than those in M9 (after 14 days without water). This faster physiological response in CG202, closing the stomata and reducing transpiration, is consistent with the earlier perception of water stress by CG202 and the subsequent hormonal changes described above, suggesting greater sensitivity to low soil water potential than that of the M9 rootstock. It is difficult to comment on the longer resistance and/or resilience to drought of these rootstocks. Drought resistance can be described as the ability to maintain growth during a drought and resilience as the ability to recover growth after a drought [46]. The gas exchange data indicated that the water stress imposed by day 14 was possibly too severe, as both rootstocks had low photosynthetic rates, transpiration rates and stomatal conductance. The plants showed physical symptoms of water stress, which in turn impaired health equally in both rootstocks, complicating any discussion of drought resistance. Whilst not a direct measurement of stomatal aperture, stomatal conductance measures the rate of gaseous exchange (e.g., CO_2_ exchange) through the stomata, with a drop indicating stomatal closure [47]. ABA-mediated stomatal closure is a common physiological response to drought [48] which aims to conserve water/limit water loss, as measured by the transpiration rate, but which also limits the gaseous exchange necessary for photosynthesis. In both trials, 14 days without water resulted in a 50% loss in pot weight, and the photosynthetic rates were significantly decreased to the same extent in both rootstocks. Visually, CG202 and M9 exhibited similar physical appearances indicative of severe water stress, i.e., leaf wilting/chlorosis, which was largely confined to the top 25% of each plant, but only non-wilted leaves were selected for all biological measurements so that these measurements were not compromised by tissue damage. However, for future studies, especially on potted plants, we would recommend less severe water stress—for example, shorter periods of drought deprivation followed by rewatering cycles [41]—and/or using water stress treatments that consist of percentage reductions in the maximum water content at pot capacity [45]. Recovery data after a period of re-watering were not collected in this study, so it was not possible to comment on drought resilience either. The recovery following dehydration stress is perhaps of greater significance in perennial crops than annuals and will be considered in our future studies. The information in the literature about the relative drought resistance and/or resilience of CG202 and M9 is conflicting, with Xu and Ediger [41] suggesting that M9 is more drought-resistant than CG202, which they defined as involving more efficient CO_2_ assimilation; higher net photosynthesis; smaller declines in stomatal conductance; i.e., more stringent stomatal control; and reduced water use/lower transpiration rates during stress. In contrast, Choi et al. [42] focused on measuring the soil and water potential, water use efficiency (WUE), vegetative growth and dry matter, both during drought stress and recovery periods, and found CG202 to be more drought-resilient than M9. They observed that although M9 had a better WUE than that of CG202 at the height of water stress, CG202 exhibited a lower leaf:fine root ratio than that in M9, which is thought to lessen the impact of xylem embolism (air-filled tracheids and/or vessels) on impairing stem water transport, thus contributing to better recovery post-drought stress. The anomalous findings between these studies may also be associated with differences between the definitions of resistance and resilience, as well as experimental differences, including the age of the rootstock–scion systems used (1–3 years), the scions used for grafting (‘Ambrosia’ vs. ‘Fuji’) and the duration and frequency of the drought regimes imposed, as well as the highly variable environmental conditions and soil characteristics in field trials. Moreover, these results are not directly comparable to those from the current study on rootstocks alone because the published studies were conducted on grafted rootstock–scions. However, whilst the scion will affect the results, generally, the rootstock is considered to play a more important role in determining drought resilience in grafted apples [39,49]. Interestingly, Xu and Ediger [41] found that the rootstock choice influenced both stomatal size and density on a common scion, Ambrosia™, with higher-density and smaller-sized stomata found on ‘Ambrosia’ leaves grafted onto M9 versus those grafted onto CG202. This may in part explain the more stringent stomatal control that they observed in the ‘Ambrosia’/M9 grafted plants.

To select suitable (transcriptional) gene markers, potential candidates needed to show convincing responses to the transcriptional regulation/mRNA expression associated with the drought response, i.e., statistically significant differential expression, consistent patterns over the two trials (for genes common to both gene sets) and responses that mirrored physiological and biochemical changes. Gene families that met these requirements were the PYL4, CYP707A1/A2, NCED3, RD29B, MdbHLH130 and SDH1 gene families. Apart from the first gene family, these all started with low basal levels of expression (less than 50 copies) but were significantly upregulated with drought, with a faster and more elevated response occurring in the root tissue from CG202 than that from M9. The same trends were apparent in the leaf tissue from both rootstocks, but overall, the upregulation was lower than that in the root tissue, although the expression within some gene families was sometimes tissue- and isoform-specific. The PYL4 gene family was expressed at high basal levels in the roots (up to 5000 copies in both rootstocks) and leaves (up to 1000 copies in both CG202 and M9) of the irrigated plants and decreased significantly with drought, with greater downregulation occurring in GC202 than M9. The same pattern of downregulation was also found for this subfamily in Arabidopsis [50]. All of these gene families, except SDH1, are directly or indirectly involved in ABA metabolism. As well as modulating stomatal closure, increased ABA concentrations are known to increase the root:shoot ratio and promote the growth of the lateral roots in Arabidopsis [11]. This study did not analyse the transcriptional expression of genes where the predominant modes of regulation identified in the literature involved post-transcriptional, post-translational or epigenetic changes.

The differential expression of genes in the dry treatment versus the control was greater in trial 1 than that in trial 2, and the expression of phytohormone drought markers was also delayed in trial 2. This was reflected in the environmental data, where the temperature was hotter in trial 1 than that in trial 2, especially during the first few days, when the maximum temperature differed by approximately 8 °C and the minimum temperature by 10 °C degrees. High temperatures often accompany drought in nature and may have had an additive effect [51] on the stress response seen in trial 1. This is because elevated temperatures increase water loss through transpiration, thus amplifying the effect of water stress in plants facing drought [52]. However, plants’ physiological and molecular responses to multi-factorial stresses are primarily determined by the most severe stress factor [53], which in this study was drought. Since there were insufficient data to interrogate the temperature/drought interaction, our focus was on the consistent responses to the most severe stress (drought) across the two trials. Statistical comparisons between trials 1 and 2 were not made because of the many variables that would complicate the interpretation, e.g., different plants, seasonal factors and temperature differences. Further experiments are required to compare the effects of an elevated temperature and drought, individually and combined, on physiological, transcriptional and hormonal responses.

The CYP707A1/A2 genes were significantly upregulated by drought, especially in CG202 vs. M9 roots. The CYP707A1/A2 enzyme groups are key ABA catabolic genes encoding ABA 8′-hydroxylases that convert biologically active ABA into an unstable intermediate 8′-hydroxy-ABA that is cyclized into the inert PA metabolite [54]. Endogenous concentrations of ABA are thought to be controlled by the balance between biosynthesis and catabolism; transport to different parts of the plant; and cycling between inert glycosylated storage forms (ABA–glucose ester) and more biologically active aglycone pools [11]. However, the role of the CYP707A family is so crucial to regulating internal ABA concentrations that mutants deficient in CYP707A activity accumulate more ABA than lines overexpressing ABA biosynthesis enzymes do [11]. Different CYP707A enzyme groups are known to have different spatial and temporal patterns of expression that reflect their slightly different physiological roles [11]. For instance, CYP707A1 is most important to ABA catabolism midway during seed development and is localised within the embryo, whilst CYP707A2 regulates ABA levels during the late stages of seed maturation/germination and is found in both the embryo and the endosperm in Arabidopsis [55]. The transcript levels of all CYP707A groups were induced by dehydration in Arabidopsis, with CYP707A1 shown to play an important role in regulating ABA pools in the stomatal guard cells [56]. In the current study, tissue-differentiated gene expression patterns were also observed for the different CYP707A groups and isoforms within those groups. The basal expression levels of *CYP707A1_ii* and *CYP707A2_i* were up to 240-fold more abundant in the leaves than the roots and remained high in the leaves throughout the experiment but increased significantly in roots in the dry treatment. The reverse was true for *CYP707A1_i*, where its baseline levels were up to 120-fold greater in the roots than the leaves, and transcript numbers decreased by up to 50% in the roots in the dry treatment. These observations may suggest a more fundamental role of *CYP707A1_ii* and *CYP707A2_i* in regulating ABA in the leaf guard cells, whereas the reduced abundance of *CYP707A1_i* in the roots in the dry treatment would lead to increased ABA concentrations, possibly promoting adventitious rootformation.

All isoforms in the NCED3 family were also significantly upregulated in the roots in the dry treatment, and *NCED3_iii* was upregulated in the leaves, with greater upregulation in CG202 than that in M9. The NCED3 family is the key regulator of ABA synthesis in Arabidopsis, with increased ABA synthesis being a pivotal response to drought stress [12,13,14]. Recent CRISPR-cas knock-out and overexpression experiments in model species such as rice [57] indicate that OsNCED3 (one of three paralogs of dicot NCED3) plays a vital role in water stress tolerance. The upregulation of ABA synthesis was greater in the roots rather than that in the leaves in both rootstocks, but especially in GC202, a result mirrored in a drought study in kiwifruit [58]. Although ABA is thought to be predominantly synthesised in the leaves where the stomata are located, synthesis in the roots does also occur [59]. Moreover, ABA’s synthesis in one part of the plant and its subsequent transport to another are well-established phenomena, with Manzi et al. [60] showing that hormonal transport from the aerial organs contributed to sustained ABA accumulation in long-term drought-stressed tomato roots and Hu et al. [59]’s data supporting ABA transport in the reverse direction. In addition, Hu et al. [59] showed that the initial site of water stress governs the pattern of ABA synthesis, with synthesis occurring in the roots first when the root tissue is directly stressed and vice versa when water stress is imposed directly onto peanut leaves. In the current study, the roots were the first part of the plant to sense the water shortages and therefore could be expected to respond strongly/rapidly in terms of ABA synthesis, with a stronger response induced in CG202 than that in M9. Further spatial expression profiles underlying the ABA synthesis/catabolism in different tissue types could be an interesting avenue for further study. Overall, the significant increase in both NCED3-mediated ABA biosynthesis and CYP707A catabolysis suggests that drought appears to stimulate ABA metabolism as a whole, with a stronger transcriptomic response occurring in CG202 (especially in the roots) than that in M9.

Response to Dehydration 29B, isoform i (*RD29B_i*) was upregulated in both trials in the current study, with the strongest responses occurring in the roots of CG202. The RD29B family contains ABA-dependent genes involved in the downstream parts of the ABA signalling cascade that have been shown to be upregulated in response to both drought and to the application of an effective drought priming agent (a plant-growth-promoting rhizobacterium) in Arabidopsis [61]. The RD29B phylogeny tree in apple consists of two distinct branches, both of which are quite distant from RD29B in Arabidopsis, indicating evolutionary divergence. Only one of the family members, *RD29B_i*, was significantly upregulated in response to drought in the current study and mainly in the roots, which possibly points to the different tissue-specific roles played by members of the same gene family, with a strong drought-induced response in the root tissue.

bHLH130, a TF, is thought to be involved in stomatal regulation, with bHLH TFs identified as contributing to improved drought tolerance in transgenic apple calli [3]. Dehydration-induced bHLH130 has been shown to regulate stomatal closure and increase the expression of ROS-scavenging and stress-related genes in tobacco, leading to increased tolerance to drought [62], and belongs to the same clade as ABA-Responsive Kinase Substrate 1 (AKS1) from Arabidopsis. Takahashi et al. [63] showed that AKS1 is modulated by ABA, which causes the inhibition of KAT1 expression by releasing AKS1 from interaction with the KAT1 protein, thereby limiting stomatal opening. The basis of this release is the monomerization of AKS1 through phosphorylation by ABA-responsive SnRK2 kinases. This stomatal response mediated by AKS1 is likely a late process and may, for example, reduce the reopening of the stomata after severe drought episodes. In the current study, more than 2-fold significant upregulation of *MdbHLH130* was observed only in the roots of GC202, and in M9 to a lesser extent, which did not fit with the leaf stomatal response theory. However, the baseline abundance of *MdbHLH130* was up to 2.8-fold greater in the leaves than in the roots, suggesting that its main role in stomatal closure regulation in the leaves is a basal response rather than an induced response. This gene marker was only introduced into the second gene set used to probe trial 2, so further trial data are required to verify this result.

Sorbitol dehydrogenase (SDH1) is involved in sugar metabolism, converting sorbitol into fructose [64]. This marker was newly introduced in trial 2 and was the most abundantly expressed of all of the genes in that trial, with the greatest expression occurring in the roots and leaves from GG202 (basal levels of around 10,000 copies increased to around 30,000 copies after drought). The high abundance of SDH1 in the root and leaf tissues is consistent with the central role of sugar metabolism in plant growth and source/sink relationships. Potential dual roles of this enzyme in the drought response could include modulating cellular osmotic adaptation, which is a common response to water deficiency, and/or fructose may act as an emergency store of energy that can be utilised after an initial increase in sorbitol to adapt to water stress. Emergency energy stores become particularly important once photosynthesis is limited by the reduced external gaseous exchange associated with stomatal closure.

The PYL4 family acted as a negative marker of drought response, with significant downregulation occurring with drought in both rootstocks and tissue types, but with a more extreme and rapid response occurring in CG202 than that in M9, especially in the root tissue. A particular class of protein phosphatases (PP2Cs of the A clade) inhibits a key node of the ABA response by binding SnRK2 kinases, preventing these kinases from activating downstream ABA signalling responses [11]. These PP2Cs are kept quiescent and effectively sequestered by the PYR/PYL ABA receptor family in the presence of ABA. There are different classes of PYR/PYL ABA receptor proteins, which include dimeric and monomeric classes, as well as classes with different affinities to ABA and different expression profiles. The dimeric PYL classes (e.g., PYR1/PYL1-2) show lower affinity/higher dissociation constants for ABA (>50 µM) than monomeric PYLs do (e.g., PYL4, PYL9, ~1 µM), but in the presence of their matching PP2C-A clade protein partners, they form ternary (PP2C-sequestering) complexes with much higher ABA affinities, in the 30–60 nM range [65,66]. Dimeric PYR/PYLs are compromised on the surface that interacts with their PP2Cs partners and are therefore highly dependent upon ABA to adopt PP2C-binding/sequestering conformations. Monomeric receptors are able to interact to some degree with PP2Cs even in the absence of ABA. Monomeric PYL4 shows high expression across multiple tissues and therefore probably plays a critical role in sequestering PP2C-As [67]. In support of this theory, the four members of the PYL4 family tested in this study also showed high basal expression in the roots (up to 5000 copies) and leaves (up to 3000 copies) of both rootstocks. In maize, PYL4-like genes showed differential expression patterns in the leaves, with some members of the PYL4 family showing decreases in their response to ABA while others showed increases [68], whilst in the roots, PYL4 showed dose-dependent ABA downregulation. PYL4 is rapidly downregulated in response to drought in Arabidopsis [50], and drought-induced downregulation of the PYL4 family was also observed in the roots and leaves of another dicot, kiwifruit, with the degree of the response being more extreme in the roots than the leaves [58]. Interestingly, the PYL9 family is thought to play an important role in regulating lateral root growth, and coordinated signals sent through PYL9 are an important component of drought recovery [69], with the overexpression of PYL9 also shown to provide tolerance to drought in both Arabidopsis and rice [70]. Recent CRISPR-cas knock-out experiments of rice PYL proteins indicate that some poly-mutants like PYL1/4/6 are more sensitive to drought [71]. The PYL9 genes tested in trial 1 did not show any significant differential response to drought and so were excluded from trial 2. One possible explanation for this anomaly is that the complete family of PYL9-like genes in apple was not included in this study, and rather, representative members chosen from each of the distinct branches in the phylogenetic tree were used. It is therefore possible that we did not select the most responsive members of this gene family. Future studies could involve comprehensive screening of all PYL9-like genes in apple to identify the most drought-responsive isoforms.

Four main classes of phytohormones and their metabolites were quantified in this study, and their compositions were affected by tissue type, rootstock genotype and drought treatment, with consistent trends across both trials. In both genotypes, ABA and SA derivatives accumulated in the leaves, while higher concentrations of jasmonates and IAA were quantified in the roots. While these phytohormones can be produced in multiple cell types and organs, they are predominantly synthesised in the chloroplasts (SA, JA) or leaf cells (ABA, IAA) and transported where needed [72]. The root tips also synthesise IAA as a key regulator of root development in crosstalk with ethylene and jasmonates. While the most bioactive jasmonate form, JA-Ile, is mainly known for its roles in plant defences against biotic stresses, it also promotes lateral root formation by inhibiting primary and adventitious roots in crosstalk with auxin [73]. In this study, young/developing root tissue was sampled in contrast to mature leaves, which may explain the elevated concentrations of IAA and JA-Ile in the actively growing root samples. The genotype comparison showed that CG202 produced constitutively higher concentrations of abscisates and jasmonates in both the roots and leaves, while M9 had a higher SA content, especially in its leaves. A recent study highlighted the increased pest resistance of CG202 [74], which is likely instigated by its higher basal levels of JA-Ile and demonstrates the multifunctional role of this phytohormone. SA is also commonly associated with its role in mitigating biotic stresses; however, it is also known to induce stomatal closure and consequently reduce photosynthetic activity in a variety of crop plants [75]. Higher basal concentrations of SA might compensate for lower ABA in regulating stomata movement in M9 when compared to that in CG202.

ABA is the primary phytohormone associated with drought stress and stomatal closure [11] in plants, and in this study, its concentration increased rapidly in the roots and after 9 days in the leaves of both rootstock genotypes in response to water limitation. This was also observed for its catabolite PA, which forms spontaneously from 8′-hydroxy-ABA (8-OH-ABA) and might exhibit some residual physiological activity to extend ABA-regulated functions [76]. Both phytohormones were recognised as positive chemical markers of drought responses in the roots and leaves. Cytochrome P450 monoxidases catalyse the hydroxylation of ABA into 8-OH-ABA and are encoded by the CYP707A gene family. CYP707A2 was identified as its most drought-responsive member in this study, with its transcript levels being magnitudes higher in CG202 than those in M9. Together with the increased expression of the ABA biosynthesis gene NCED3_iii in droughted CG202, these findings indicate that ABA metabolism is genetically upregulated in this rootstock genotype.

In the roots only, IAA was highlighted as a positive chemical marker for drought stress, as it increased significantly after 14 days without irrigation in both genotypes. This aligns with observations in tobacco, where water deficits induced the accumulation of IAA in the roots but not in the leaves [77]. Moreover, drought-induced IAA was shown to increase the formation of first- and second-order lateral roots, likely as a strategy to increase water uptake, and a similar mechanism is possible for apple rootstocks.

In contrast, cis-OPDA was identified as a negative chemical marker of water limitation. While its concentrations were higher in the roots, they decreased in response to extended drought in both the leaves and roots of both genotypes. While cis-OPDA is the biosynthetic precursor of JA, it is known to act as a signalling molecule independent of JA responses. Besides acting as a transcriptional activator of stress-related genes [78], it has been shown to accumulate in the guard cells and partake in regulating stomatal closure [79], thus mitigating drought stress. Therefore, this drought-induced decrease in cis-OPDA in the apple rootstocks contrasts with the observations from studies using annual plants, which have commonly reported an increase in this oxylipin in response to stress [80], is difficult to explain and requires further investigation.

In recent years, there has been increasing interest in the use of phytohormones as priming agents to mitigate the negative effects of drought on plants [81]. ASM (a functional analogue of SA) was an excellent candidate to assess for potential drought priming in our apple system because SA has previously been shown to ameliorate the negative effects of drought through improving photosynthetic performance, stimulating SA-mediated defence responses and enhancing the activity of antioxidant enzymes [27,82]. The application of ASM also reduced transpirational water loss by inducing stomatal closure in the monocot creeping bentgrass [31,83]. However, the application of ASM did not alter any of the physiological factors measured in the current study, in contrast to the results shown in watermelon, rice, wheat, barley and tomatoes [26,28,30]. Differences between the current study and those cited might be due to the rate and frequency of ASM application and/or the timing of the biomarker measurements relative to ASM application. In this study, ASM was applied as a root drench, but ASM is more typically applied as a foliar spray; hence, further experiments should be conducted to compare foliar and soil applications before and/or during the onset of drought. However, one lasting ASM response post-application was genotype-specific and resulted in increased MeJA concentrations in the roots from both irrigated and dry M9 plants. As a precursor to JA-Ile production, MeJA might promote lateral root formation in ASM-treated M9 rootstocks, but it mitigating drought stress responses is unlikely because no other physiological, chemical or genetic markers were altered in response to its application.

An understanding of the sensitivity of the rootstocks and the dynamics of the hormone responses presented here can provide useful information to help guide irrigation strategies in the field under various water stress events. Depending on the growth stage of the crop, deficit irrigation methods such as partial root zone irrigation under mild drought conditions can maintain a moderate water supply from the ‘wetted’ roots whilst also triggering a drought sensitivity response from the ‘dry’ roots. This approach can be used to promote long-term resilience to water stress [20]. Within this strategy, there is a potential trade-off between growth and ABA-induced drought resistance; however, potential long-term benefits can include increases in water use efficiency and beneficial secondary metabolites in fruit, e.g., anthocyanins in grapes [84]. It is also worth considering the impact of partial root zone irrigation on the development and morphology of the shoots early during their growth and its effect on subsequent drought events later in the season. Smaller, thicker leaves with a thicker cuticular layer can increase the water use efficiency throughout the season [85]. Future work in this area is required to examine the usefulness and relative success of such irrigation strategies in our rootstocks with differing sensitivities to water stress, such as studies on the morphological adaptations of these rootstocks (and any grafted scions) under varying drought conditions. This study identified candidate phytohormonal and transcriptomic ‘drought’ biomarkers that correlated either positively or negatively with the physiological responses to drought in the M9 and CG202 rootstock plants. The transcriptomic biomarkers encompassed a wide range of functional processes associated with the main stress response hormone, ABA, including biosynthesis (NCED3), catabolism (CYP707A1/2) and the signalling response (PYL4, RD29B). The biochemical markers also represent three phytohormone pathways associated with abiotic stress responses, i.e., ABA, PA (the ABA pathway); cis-OPDA (the JA pathway); and IAA (auxins). The development of robust drought biomarkers is essential for facilitating the development of stress mitigation strategies that rely on increased plant tolerance to drought. This applies both to breeding programmes and the selection of chemical and biological agents that may be used to prime or condition plants for greater drought resilience, with the latter enabling the protection of existing plantings. The characterisation of the responses to moderate and severe drought across different apple rootstock genotypes can aid in the identification of genetic factors that are critical to drought tolerance. This would include integration between plant physiological, transcriptomic and phytohormonal data and environmental modelling to enable the selection of genotypes that are more suited to different drought scenarios.

## 4. Materials and Methods

### 4.1. The Plant Material and Treatment Application

#### 4.1.1. Trial 1

A set of 55 bare-rooted apple rootstocks each for M9 and CG202 was potted into 10 L plastic pots containing Daltons™ GB mix (Daltons, Matamata, New Zealand) and placed on a concrete floor in a polytunnel on dripper lines. For each pot, there were two drippers that delivered a total volume of 1.8 L water per day (900 mL delivered over a 30 min period at 4 am and 4 pm). Following budburst, each plant was reduced to two shoots.

This trial started on 9 February 2022 (day 0), 10 weeks after potting. There were two treatments, ‘irrigated’, where the pot continued to be watered, and ‘dry’, where the two drippers were removed. A Hydrosense™ soil moisture probe (Campbell Scientific Australia, Garbutt, QLD, Australia) found that a moisture content of 35–40% in an irrigated pot dropped to 3–5% by day 14 after the removal of the drippers.

There were five replicate pots for the baseline (day 0) and for each of the four sampling time points (day 2, 5, 9 and 14) at which the plant was destructively sampled of leaf and root material. The pots were arranged into a randomised design of the treatments and the two rootstock genotypes.

For the dry treatment, the drippers were removed on day 0 from 25 pots, and each pot was placed on an upturned tray to avoid accidental watering from any ground water leaking from neighbouring pots.

#### 4.1.2. Trial 2

A set of 90 bare-rooted apple rootstocks each for M9 and CG202 was potted and placed into a polytunnel on dripper lines, as in the previous trial, and the pots were arranged into a randomised design.

This trial started on 16 November 2022, 10 weeks from potting. The plants were divided into two treatment groups: (1) an untreated control and (2) an Actigard^®^ a.i. acibenzolar-S-methyl (ASM)-treated (purchased from Syngenta NZ, Auckland, New Zealand) group. ASM was applied at 10 mg of a.i./plant (100 mL of a 0.2 g/L solution of Actigard, which contained 50% a.i.) to the soil around each plant, both at 14 days prior to day 0 and again on day 0 (16 November 2022). Each treatment group was then subdivided further, with half receiving normal watering (irrigated) and the drippers removed from the other half (dry). This created four treatment groups: (1) irrigated control, (2) irrigated + ASM, (3) dry control and (4) dry + ASM.

There were five replicate pots for each of three time points (day 5, 9 and 14) at which the plant was destructively sampled of leaf and root material. For the irrigated and irrigated + ASM treatments, there was an additional time point for the baseline measurements on day 0.

### 4.2. Gas Exchange Measurements and Water Loss

Five plants for each treatment were used for repeat measurements of the pot weight, plant photosynthesis rates, stomatal conductance and transpiration rates over the trial period for both trials. Measurements were carried out on days 0, 2 (trial 1 only), 5, 9 and 14. The gas exchange measurements were carried out using a CIRAS-3 portable photosynthesis system (PP Systems, Amesbury, MA, USA). The soil water content of the irrigated and dry treatments was measured as the loss in weight over time attributed to water loss and presented as a percent reduction in the soil’s water content relative to that on day 0.

All gas exchange measurements were carried out on the same leaves for each date, and the second or third fully expanded leaves below the growing apex were selected. The day 0 measurements were carried out before the dry treatment was applied and so consisted of the irrigated plants only. All measurements were carried out between 10.30 am and 12.30 pm. The leaves were remeasured after the initial measurements to check for any photoinhibition in the later measurements. The random treatment layout ensured that all measurements were spread across the treatments during the measurement period.

### 4.3. Tissue Sampling for Phytohormone and Gene Expression Analysis

Leaves and roots were sampled at each of the time points between 10 am and noon. The leaf samples consisted of two fully expanded leaves, one from each shoot, collected approximately 15–20 cm from the shoot tip. The midribs were removed before placing the samples into vials and snap-freezing them in liquid nitrogen. The root tissue was collected after removing the plants from the pots and then washing the roots under running water. Young root tissue (approximately 1 g, located up to 5 cm from the growing tip) was removed, blotted dry and snap-frozen. For each of the two apple rootstock cultivars in trial 1, there were 45 leaf and 45 root samples, making a total of 180 samples each for the phytohormone and gene expression analyses. The samples were stored at −80 °C until processing. In trial 2, there were 70 leaf and 70 root samples, making a total of 280 samples for the phytohormone and gene expression analyses. Due to the much greater sample numbers, only three of the five replicates were processed for the gene expression in trial 2.

For processing, the tissue samples were ground into a fine powder using a mortar and pestle with liquid nitrogen. Subsamples of the ground tissue were weighed for RNA extraction and for freeze-drying for the phytohormone analyses. The samples were freeze-dried for trial 1 to normalise to changes in the tissue weight that could potentially occur due to drought-associated water loss, but fresh-weight samples were used in trial 2.

### 4.4. RNA Extraction

Total RNA was extracted from 100 mg of frozen, ground tissue using the Spectrum Plant Total RNA kit (Merck, Auckland, New Zealand), according to the manufacturer’s instructions. The RNA samples were quantified (with ≥20 ng/µL considered acceptable) and their purity assessed (with a 260/280 absorbance ratio of ~2.0 accepted as “pure”) using a NanoDrop 2000c spectrophotometer (Thermo Scientific, Waltham, MA, USA).

### 4.5. Gene Selection

Candidate genes were mined from a list of genes identified from the literature on both ABA metabolic synthesis and catabolic pathways in apple and model plants; plant responses to drought; and functional analyses of the genes involved in drought resistance (see the details in Supplementary Table S1). The two sequentially selected gene sets (one for each trial) consisted of the same 6 reference genes (RGs) that were chosen based upon the stability of their expression [86,87] and 42 genes of interest (GoIs) that were selected through a process of prioritisation (similar to that outlined by Wurms et al. [58]) based on the strength of the evidence of their involvement in drought responses in plants. The selection of the GoIs was based on a broad cross-section of the responses, including both upregulated and downregulated components. In cases of larger gene families with multiple members, candidates were selected to represent distinct branches of the family’s phylogenetic tree. The selected genes used in both sets are given in Supplementary Table S1, where the key literature supporting the gene candidacy is also identified for each chosen gene. The genes in set 2 used in trial 2 were an iteration of the genes in set 1, with the non-responding genes from gene set 1 used in trial 1 replaced with new candidates mined from the literature (Table S1).

### 4.6. Measurement of Gene Expression Using the PlexSet Nanostring

Measurement of the direct counts of the genes expressed was carried out on a NanoString nCounter^®^ platform (NanoString Technologies, Seattle, WA, USA) using PlexSet^®^ chemistry, following the steps of probe hybridisation, titration to determine the optimal RNA input, sample immobilisation onto a cartridge and digital counting, as described by Wurms et al. [58]. The only differences were that the RNA input was optimised at 280 ng per sample in trial 1 and 320 ng in trial 2. The results were normalised against the in situ system positive controls and the most stably expressed RGs, which comprised *CKB4*, *FYPP3* and *GPAT1* in trial 1 and *CKB4*, *FYPP3*, *GPAT1*, *LTL1* and *Protein GRIP* in trial 2.

### 4.7. Phytohormone Extraction and Analysis

The phytohormones in trial 1 were extracted and quantified through liquid chromatography mass spectrometry (LCMS) on a 5500 QTrap triple-quadrupole/linear ion trap (QqLIT) mass spectrometer equipped with a Turbo V™ ion-source ESI probe (AB Sciex, Concord, ON, Canada) coupled to a Shimadzu Exion UHPLC (Shimadzu, Tokyo, Japan), as described in Wurms et al. [58], with modifications to the sample weight extracted (trial 1, 100 mg dry weight, DM).

For trial 2, the methodology was modified to expand the scope of the targeted analytes to include neophaseic acid (neoPA) and to expand the scope of the labelled internal standards added at extraction ([^2^H_3_]-DPA 5 ng, [^2^H_3_]-PA 2 ng, [^2^H_3_]-neoPA 2 ng, [^2^H_4_]-7-OH-ABA 5 ng, [^2^H_5_]-ABA-GE 5 ng). For trial 2, the LCMS experiments were carried out on a 7500 QTrap QqLIT mass spectrometer equipped with a Turbo V™ ion-source ESI probe (AB Sciex, Concord, ON, Canada) coupled to a Shimadzu Nexera LC40 UHPLC (Shimadzu, Tokyo, Japan), using material extracted from 100 mg of fresh weight, FW.

### 4.8. The Statistical Analysis and Data Visualisation

Phytohormone and gene expression data from trials 1 and 2 were log-transformed (to stabilise the variance) and analysed using a multi-factor analysis of variance. The factors in trial 1 were rootstock genotype, irrigated/dry treatment and sampling day, plus their interactions. The factors in trial 2 were rootstock genotype, irrigated/dry treatment, ASM treatment and day, plus their interactions. As the treatments did not apply on day 0, treatment and day were not completely orthogonal, and so the effects were tested and adjusted for other factors.

The means were summarised as fold changes, either compared to the control treatment on the same rootstock on the same sample day or between the two rootstocks on the same treatment on the same day. The least significant differences (*p* = 0.05) from the models corresponded to fold changes between 1.5- and 3-fold, so a 2-fold change (positive or negative) was taken as the threshold for reporting.

This analysis was performed using Genstat, version 22, (VSNi Ltd., London, UK, 2022).

For the gas exchange data, an analysis of variance was carried out separately for the responses of photosynthesis, transpiration and stomatal conductance to the effects of rootstock (CG202 and M9), assessment time (days), ASM treatment (ASM and none; trial 2 only) and water treatment (watered vs. drought conditions). Pairwise linear contrasts were used to assess significant differences between the means for the watered and droughted treatments for each combination of rootstock and assessment day, for an α = 0.05 level of significance. The analyses were carried out in Genstat 24th Edition (VSN International 2025).

The phytohormone data were analysed and visualised using R version 4.4.2. A small offset was used for concentrations below the limit of detection (LOD), which were replaced with 0.2* LOD. The data were log_10_-transformed and standardised through autoscaling (mean-centred and feature-scaled) using the ‘mdatools’ package [88]. Heat maps were computed based on the Euclidean distances of the means and clustered using Ward’s method, as implemented in the ‘gplots’ package [89]. A redundancy analysis (RDA) was performed using ‘vegan’ [90] to analyse the relationships between phytohormones and rootstock genotype, treatment and sampling day as explanatory variables. The ‘permutest’ function was used to assess the significance of the model with 9999 permutations, and the significance of the constraints was computed using ANOVA-like permutation testing by ‘term’ (explanatory variables) and ‘axis’ (component) at α = 0.05. The proportion of variance explained by each RDA axis was computed based on the eigenvalues and ordination scores ($site) and visualised as RDA sample plots. Data ellipses were computed at a confidence level of 95%. Biplot visualisation was used to graph the ordination of the explanatory variables and response variables ($species) as loading vectors.

Univariate, pairwise comparisons were made for the log10-transformed data using the ‘tukey-HSD’ function with false discovery rate correction for multiple comparisons at α = 0.05, as implemented in ‘rstatix’ [91]. Line graphs of the raw data were prepared using ‘ggplot2’ [92] with the ‘geom-smooth’ function and a default confidence level of 95%.

## Figures and Tables

**Table 1:** Change in mean pot weight (%) ± standard error (SE) of droughted (dry) plants over time relative to that for watered (irrigated) plants at time 0 in two apple rootstocks, CG202 and M9, in trial 1.

Rootstock	±Water	% Weight Relative to Controls at T0 ^1^				
		0 d	2 d	5 d	9 d	14 d
CG202	Irrigated	-	103 ± 0.7	103 ± 0.6	103 ± 0.5	103 ± 0.8
	Dry	100 ± 2.9	89 ± 2.5	79 ± 2.2	60 ± 1.9	52 ± 1.7
M9	Irrigated	-	100 ± 1.3	100 ± 1.3	99 ± 1.0	103 ± 1.7
	Dry	100 ± 1.3	91 ± 1.4	82 ± 1.5	65 ± 1.8	54 ± 1.1

^1^ Average weight (n = 5) at T0: CG202 = 6.99 kg, M9= 7.19 kg. Least significant difference (LSD) when comparing between rootstocks = 5.0, LSD when comparing within rootstocks = 1.8.

**Table 2:** Change in mean pot weight (%) ± SE of droughted (dry) plants over time relative to that for watered (irrigated) plants at time 0 in two apple rootstocks in trial 2.

Rootstock	±Water	±ASM	% Weight Relative to Controls at T0 ^1^		
			5 d	9 d	14 d
CG202	Irrigated	No ASM	99 ± 1.4	101 ± 1.8	100 ± 1.1
		ASM	100 ± 1.7	101 ± 1.4	100 ± 0.8
	Dry	No ASM	77 ± 2.4	63 ± 1.6	56 ± 1.7
		ASM	78 ± 1.7	64 ± 2.3	55 ± 1.2
M9	Irrigated	No ASM	100 ± 1.2	98 ± 1.5	99 ± 1.4
		ASM	100 ± 1.6	98 ± 2.0	99 ± 1.7
	Dry	No ASM	79 ± 2.0	68 ± 1.6	59 ± 3.4
		ASM	82 ± 1.7	69 ± 0.4	61 ± 0.9

^1^ Average weight (n = 5) at T0: CG202 = 6.38 Kg, M9 = 6.35 Kg. LSD when comparing between rootstocks = 4.8, LSD when comparing within rootstocks = 4.2.

## Data Availability

Data is contained within the article and Supplementary Material.

## Supplementary Materials

The supporting information can be downloaded at https://www.mdpi.com/article/10.3390/ijms26146986/s1.

## References

[1] Ji X.L., Li H.L., Qiao Z.W., Zhang J.C., Sun W.J., Wang C.K., Yang K., You C.X., Hao Y.J. (2020). The BTB-TAZ protein MdBT2 negatively regulates the drought stress response by interacting with the transcription factor MdNAC143 in apple. Plant Sci..

[2] Jia X., Mao K., Wang P., Wang Y., Jia X., Huo L., Sun X., Che R., Gong X., Ma F. (2021). Overexpression of *MdATG8i* improves water use efficiency in transgenic apple by modulating photosynthesis, osmotic balance, and autophagic activity under moderate water deficit. Hortic. Res..

[3] Li X., Ma Z., Song Y., Shen W., Yue Q., Khan A., Tahir M., Wang X., Malnoy M., Ma F. (2023). Insights into the molecular mechanisms underlying responses of apple trees to abiotic stresses. Hortic. Res..

[4] Singh D., Laxmi A. (2015). Transcriptional regulation of drought response: A tortuous network of transcriptional factors. Front. Plant Sci..

[5] Kim W., Iizumi T., Nishimori M. (2019). Global Patterns of Crop Production Losses Associated with Droughts from 1983 to 2009. J. Appl. Meteorol. Climatol..

[6] Espinoza-Meza S., Ortega-Farias S., López-Olivari R., Araya-Alman M., Carrasco-Benavides M. (2023). Response of fruit yield, fruit quality, and water productivity to different irrigation levels for a microsprinkler-irrigated apple orchard (cv. Fuji) growing under Mediterranean conditions. Eur. J. Agron..

[7] Suran P., Pravcova G. (2023). Drought stress affects productivity and fruit size of new apple cultivars. Acta Hortic..

[8] Khan A.A., Wang Y.-F., Akbar R., Alhoqail W.A. (2025). Mechanistic insights and future perspectives of drought stress management in staple crops. Front. Plant Sci..

[9] Urano K., Maruyama K., Jikumaru Y., Kamiya Y., Yamaguchi-Shinozaki K., Shinozaki K. (2017). Analysis of plant hormone profiles in response to moderate dehydration stress. Plant J..

[10] Zandalinas S.I., Sengupta S., Fritschi F.B., Azad R.K., Nechushtai R., Mittler R. (2021). The impact of multifactorial stress combination on plant growth and survival. New Phytol..

[11] Finkelstein R. (2013). Abscisic acid synthesis and response. Arab. Book Am. Soc. Plant Biol..

[12] Hewage K.A.H., Yang J.-F., Wang D., Hao G.-F., Yang G.-F., Zhu J.-K. (2020). Chemical Manipulation of Abscisic Acid Signaling: A New Approach to Abiotic and Biotic Stress Management in Agriculture. Adv. Sci..

[13] Todaka D., Takahashi F., Yamaguchi-Shinozaki K., Shinozaki K., Seo M., Marion-Poll A. (2019). Chapter three—ABA-responsive gene expression in response to drought stress: Cellular regulation and long-distance signaling. Advances in Botanical Research.

[14] Ali S., Hayat K., Iqbal A., Xie L.N. (2020). Implications of abscisic acid in the drought stress tolerance of plants. Agronomy.

[15] Shu K., Zhou W.G., Chen F., Luo X.F., Yang W.Y. (2018). Abscisic Acid and Gibberellins Antagonistically Mediate Plant Development and Abiotic Stress Responses. Front. Plant Sci..

[16] Verma V., Ravindran P., Kumar P.P. (2016). Plant hormone-mediated regulation of stress responses. BMC Plant Biol..

[17] Conrath U., Beckers G.J., Langenbach C.J., Jaskiewicz M.R. (2015). Priming for enhanced defense. Annu. Rev. Phytopathol..

[18] Mauch-Mani B., Baccelli I., Luna E., Flors V., Merchant S.S. (2017). Defense Priming: An Adaptive Part of Induced Resistance. Annual Review of Plant Biology.

[19] Savvides A., Ali S., Tester M., Fotopoulos V. (2016). Chemical Priming of Plants Against Multiple Abiotic Stresses: Mission Possible?. Trends Plant Sci..

[20] Yang B., Fu P., Lu J., Ma F., Sun X., Fang Y. (2022). Regulated deficit irrigation: An effective way to solve the shortage of agricultural water for horticulture. Stress Biol..

[21] Godoy F., Olivos-Hernández K., Stange C., Handford M. (2021). Abiotic Stress in Crop Species: Improving Tolerance by Applying Plant Metabolites. Plants.

[22] Antonic D., Milosevic S., Cingel A., Lojic M., Trifunovic-Momcilov M., Petric M., Subotic A., Simonovic A. (2016). Effects of exogenous salicylic acid on *Impatiens walleriana* L. grown in vitro under polyethylene glycol-imposed drought. S. Afr. J. Bot..

[23] Rao S.R., Qayyum A., Razzaq A., Ahmad M., Mahmood I., Sher A. (2012). Role of foliar application of salicylic acid and l-tryptophan in drought tolerance of maize. J. Anim. Plant Sci..

[24] Aremu A., Fawole O., Makunga N., Masondo N., Moyo M., Buthelezi N., Amoo S., Spíchal L., Dolezal K. (2020). Applications of Cytokinins in Horticultural Fruit Crops: Trends and Future Prospects. Biomolecules.

[25] Sah S., Reddy K., Li J. (2016). Abscisic Acid and Abiotic Stress Tolerance in Crop Plants. Front. Plant Sci..

[26] Khan M., Fatma M., Per T., Anjum N., Khan N. (2015). Salicylic acid-induced abiotic stress tolerance and underlying mechanisms in plants. Front. Plant Sci..

[27] Rai G., Magotra I., Khanday D., Choudhary S., Bhatt A., Gupta V., Rai P., Kumar P. (2024). Boosting Drought Tolerance in Tomatoes through Stimulatory Action of Salicylic Acid Imparted Antioxidant Defense Mechanisms. Agronomy.

[28] Silva J., da Silva G.J., Bonifácio A., Dutra A., Prado R., Neto F., Zuffo A., Melo R., Pereira T., de Sousa R. (2023). Exogenous salicylic acid alleviates water stress in watermelon plants. Ann. Appl. Biol..

[29] Song W., Shao H., Zheng A., Zhao L., Xu Y. (2023). Advances in roles of salicylic acid in plant tolerance responses to biotic and abiotic stresses. Plants.

[30] Szepesi A., Csiszár J., Gémes K., Horváth E., Horváth F., Simon M., Tari I. (2009). Salicylic acid improves acclimation to salt stress by stimulating abscisic aldehyde oxidase activity and abscisic acid accumulation, and increases Na^+^ content in leaves without toxicity symptoms in *Solanum lycopersicum* L.. J. Plant Physiol..

[31] Jespersen D., Yu J., Huang B. (2017). Metabolic Effects of Acibenzolar-S-Methyl for Improving Heat or Drought Stress in Creeping Bentgrass. Front. Plant Sci..

[32] Sakata N., Ishiga T., Taniguchi S., Ishiga Y. (2020). Acibenzolar-S-Methyl Activates Stomatal-Based Defense Systemically in Japanese Radish. Front. Plant Sci..

[33] Reglinski T., Wurms K.V., Vanneste J.L., Chee A.A., Schipper M., Cornish D., Yu J.E., McAlinden J., Hedderley D. (2023). Kiwifruit Resistance to *Sclerotinia sclerotiorum* and *Pseudomonas syringae* pv. actinidiae and Defence Induction by Acibenzolar-S-methyl and Methyl Jasmonate Are Cultivar Dependent. Int. J. Mol. Sci..

[34] Ebel R., Proebsting E., Evans R. (2001). Apple tree and fruit responses to early termination of irrigation in a semi-arid environment. Hortscience.

[35] Lopez G., Boini A., Manfrini L., Torres-Ruiz J., Pierpaoli E., Zibordi M., Losciale P., Morandi B., Corelli-Grappadelli L. (2018). Effect of shading and water stress on light interception, physiology and yield of apple trees. Agric. Water Manag..

[36] Volz R. Streamlining the Breeding Process. https://www.sciencelearn.org.nz/videos/428-streamlining-the-breeding-process#more.

[37] Fenning T., Gershenzon J. (2002). Where will the wood come from? Plantation forests and the role of biotechnology. Trends Biotechnol..

[38] Jones H. (2012). How do rootstocks control shoot water relations?. New Phytol..

[39] Valverdi N., Kalcsits L. (2021). Apple rootstock genotype affects scion responses to water limitations under field conditions. Acta Physiol. Plant..

[40] Tworkoski T., Fazio G., Glenn D. (2016). Apple rootstock resistance to drought. Sci. Hortic..

[41] Xu H., Ediger D. (2021). Rootstocks with Different Vigor Influenced Scion-Water Relations and Stress Responses in Ambrosia^TM^ Apple Trees (*Malus Domestica* var. Ambrosia). Plants.

[42] Choi B., Bhusal N., Jeong W., Park I., Han S., Yoon T. (2020). Drought Tolerance of ‘Fuji’ Apple Trees Grafted onto G, CG, or M Series Rootstocks: Growth and Physiology. Hortic. Sci. Technol..

[43] Shi C., Liu L., Li Q., Wei Z., Gao D. (2022). Comparison of drought resistance of rootstocks ‘M9-T337’ and ‘M26’ grafted with ‘Huashuo’ apple. Hortic. Environ. Biotechnol..

[44] Sun P., Tahir M., Lu X., Liu Z., Zhang X., Zuo X., Shao Y., Xiao X., An N., Wang C. (2022). Comparison of leaf morphological, anatomical, and photosynthetic responses to drought stress among eight apple rootstocks. Fruit Res..

[45] Wright D., Cline J., Earl H. (2019). Physiological responses of four apple (*Malus* x *domestica* Borkh.) rootstock genotypes to soil water deficits. Can. J. Plant Sci..

[46] Chen J., Liu Z., Mao J., Zhao T., Tu T., Cheng L., Dong C. (2023). Co-regulation of water and energy in the spatial heterogeneity of drought resistance and resilience. Environ. Res. Lett..

[47] Farquhar G., Sharkey T. (1982). Stomatal conductance and photosynthesis. Annu. Rev. Plant Physiol..

[48] Liang X., Wang D., Ye Q., Zhang J., Liu M., Liu H., Yu K., Wang Y., Hou E., Zhong B. (2023). Stomatal responses of terrestrial plants to global change. Nat. Commun..

[49] Fernandez R., Perry R., Flore J. (1997). Drought response of young apple trees on three rootstocks: Growth and development. J. Am. Soc. Hortic. Sci..

[50] Goda H., Sasaki E., Akiyama K., Maruyama-Nakashita A., Nakabayashi K., Li W., Ogawa M., Yamauchi Y., Preston J., Aoki K. (2008). The AtGenExpress hormone and chemical treatment data set: Experimental design, data evaluation, model data analysis and data access. Plant J..

[51] Prasad P.V.V., Pisipati S.R., Momcilovic I., Ristic Z. (2011). Independent and Combined Effects of High Temperature and Drought Stress During Grain Filling on Plant Yield and Chloroplast EF-Tu Expression in Spring Wheat. J. Agron. Crop Sci..

[52] Hatfield J., Prueger J. (2015). Temperature extremes: Effect on plant growth and development. Weather. Clim. Extrem..

[53] Jiang Z., van Zanten M., Sasidharan R. (2025). Mechanisms of plant acclimation to multiple abiotic stresses. Commun. Biol..

[54] Marion-Poll A., Leung J. (2018). Abscisic acid synthesis, metabolism and signal transduction. Annu. Plant Rev. Online.

[55] Okamoto M., Kuwahara A., Seo M., Kushiro T., Asami T., Hirai N., Kamiya Y., Koshiba T., Nambara E. (2006). CYP707A1 and CYP707A2, which encode abscisic acid 8′-hydroxylases, are indispensable for proper control of seed dormancy and germination in Arabidopsis. Plant Physiol..

[56] Okamoto M., Tanaka Y., Abrams S.R., Kamiya Y., Seki M., Nambara E. (2009). High Humidity Induces Abscisic Acid 8′-Hydroxylase in Stomata and Vasculature to Regulate Local and Systemic Abscisic Acid Responses in Arabidopsis. Plant Physiol..

[57] Huang Y., Guo Y., Liu Y., Zhang F., Wang Z., Wang H., Wang F., Li D., Mao D., Luan S. (2018). 9-*cis*-Epoxycarotenoid Dioxygenase 3 Regulates Plant Growth and Enhances Multi-Abiotic Stress Tolerance in Rice. Front. Plant Sci..

[58] Wurms K.V., Reglinski T., Buissink P., Chee A.A., Fehlmann C., McDonald S., Cooney J., Jensen D., Hedderley D., McKenzie C. (2023). Effects of Drought and Flooding on Phytohormones and Abscisic Acid Gene Expression in Kiwifruit. Int. J. Mol. Sci..

[59] Hu B., Cao J., Ge K., Li L. (2016). The site of water stress governs the pattern of ABA synthesis and transport in peanut. Sci. Rep..

[60] Manzi M., Lado J., Rodrigo M., Zacarías L., Arbona V., Gómez-Cadenas A. (2015). Root ABA Accumulation in Long-Term Water-Stressed Plants is Sustained by Hormone Transport from Aerial Organs. Plant Cell Physiol..

[61] Liu W., Thapa P., Park S.-W. (2023). *RD29A* and *RD29B* rearrange genetic and epigenetic markers in priming systemic defense responses against drought and salinity. Plant Sci..

[62] Zhao Q., Fan Z., Qiu L., Che Q., Wang T., Li Y., Wang Y. (2020). *MdbHLH130*, an Apple bHLH Transcription Factor, Confers Water Stress Resistance by Regulating Stomatal Closure and ROS Homeostasis in Transgenic Tobacco. Front. Plant Sci..

[63] Takahashi Y., Ebisu Y., Shimazaki K.-i. (2017). Reconstitution of Abscisic Acid Signaling from the Receptor to DNA via bHLH Transcription Factors. Plant Physiol..

[64] Yang J., Zhang J., Li C., Zhang Z., Ma F., Li M. (2019). Response of sugar metabolism in apple leaves subjected to short-term drought stress. Plant Physiol. Biochem..

[65] Dupeux F., Santiago J., Betz K., Twycross J., Park S.-Y., Rodriguez L., Gonzalez-Guzman M., Jensen M.R., Krasnogor N., Blackledge M. (2011). A thermodynamic switch modulates abscisic acid receptor sensitivity. EMBO J..

[66] Szostkiewicz I., Richter K., Kepka M., Demmel S., Ma Y., Korte A., Assaad F.F., Christmann A., Grill E. (2010). Closely related receptor complexes differ in their ABA selectivity and sensitivity. Plant J..

[67] Pizzio G.A., Rodriguez L., Antoni R., Gonzalez-Guzman M., Yunta C., Merilo E., Kollist H., Albert A., Rodriguez P.L. (2013). The PYL4 A194T Mutant Uncovers a Key Role of PYR1-LIKE4/PROTEIN PHOSPHATASE 2CA Interaction for Abscisic Acid Signaling and Plant Drought Resistance. Plant Physiol..

[68] Fan W., Zhao M., Li S., Bai X., Li J., Meng H., Mu Z. (2016). Contrasting transcriptional responses of PYR1/PYL/RCAR ABA receptors to ABA or dehydration stress between maize seedling leaves and roots. BMC Plant Biol..

[69] Xing L., Zhao Y., Gao J., Xiang C., Zhu J.-K. (2016). The ABA receptor PYL9 together with PYL8 plays an important role in regulating lateral root growth. Sci. Rep..

[70] Zhao Y., Chan Z., Gao J., Xing L., Cao M., Yu C., Hu Y., You J., Shi H., Zhu Y. (2016). ABA receptor PYL9 promotes drought resistance and leaf senescence. Proc. Natl. Acad. Sci. USA.

[71] Miao C., Xiao L., Huaa K., Zou C., Zhao Y., Bressan R.A., Zhu J.-K. (2018). Mutations in a subfamily of abscisic acid receptor genes promote rice growth and productivity. Proc. Natl. Acad. Sci. USA.

[72] Bittner A., Ciesla A., Gruden K., Lukan T., Mahmud S., Teige M., Vothknecht U.C., Wurzinger B. (2022). Organelles and phytohormones: A network of interactions in plant stress responses. J. Exp. Bot..

[73] Xu P., Zhao P.X., Cai X.T., Mao J.L., Miao Z.Q., Xiang C.B. (2020). Integration of Jasmonic Acid and Ethylene Into Auxin Signaling in Root Development. Front. Plant Sci..

[74] Dallabetta N. Innovative Rootstocks for Apple Crop. https://apal.org.au/wp-content/uploads/2019/08/Innovative-Rootstocks-for-Apple-crop.pdf.

[75] Akter F., Munemasa S., Nakamura T., Nakamura Y., Murata Y. (2022). Negative regulation of salicylic acid-induced stomatal closure by glutathione in Arabidopsis thaliana. Biosci. Biotechnol. Biochem..

[76] Weng J.-K., Ye M., Li B., Noel J.P. (2016). Co-evolution of Hormone Metabolism and Signaling Networks Expands Plant Adaptive Plasticity. Cell.

[77] Wang C., Zhao Y., Gu P., Zou F., Meng L., Song W., Yang Y., Wang S., Zhang Y. (2018). Auxin is Involved in Lateral Root Formation Induced by Drought Stress in Tobacco Seedlings. J. Plant Growth Regul..

[78] Boettcher C., Pollmann S. (2009). Plant oxylipins: Plant responses to 12-oxo-phytodienoic acid are governed by its specific structural and functional properties. Febs J..

[79] Savchenko T., Dehesh K. (2014). Drought stress modulates oxylipin signature by eliciting 12-OPDA as a potent regulator of stomatal aperture. Plant Signal. Behav..

[80] de Ollas C., Dodd I.C. (2016). Physiological impacts of ABA-JA interactions under water-limitation. Plant Mol. Biol..

[81] Iqbal S., Wang X., Mubeen I., Kamran M., Kanwal I., Diaz G.A., Abbas A., Parveen A., Atiq M.N., Alshaya H. (2022). Phytohormones Trigger Drought Tolerance in Crop Plants: Outlook and Future Perspectives. Front. Plant Sci..

[82] Khalil N., Elhady S., Diri R., Fekry M., Bishr M., Salama O., El-Zalabani S. (2022). Salicylic Acid Spraying Affects Secondary Metabolites and Radical Scavenging Capacity of Drought-Stressed *Eriocephalus africanus* L. *Agronomy*
**2022**, *12*, 2278. Agronomy.

[83] Shekoofa A., Rosas-Anderson P., Carley D.S., Sinclair T.R., Rufty T.W. (2016). Limited transpiration under high vapor pressure deficits of creeping bentgrass by application of Daconil-Action^®^. Planta.

[84] Guo S.-H., Yang B.-H., Wang X.-W., Li J.-N., Li S., Yang X., Ren R.-H., Fang Y.-L., Xu T.-F., Zhang Z.-W. (2021). ABA signaling plays a key role in regulated deficit irrigation-driven anthocyanins accumulation in ‘Cabernet Sauvignon’ grape berries. Environ. Exp. Bot..

[85] Alongi F., Petek-Petrik A., Mukarram M., Torun H., Schuldt B., Petrik P. (2025). Somatic drought stress memory affects leaf morpho-physiological traits of plants via epigenetic mechanisms and phytohormonal signalling. Plant Gene.

[86] Bowen J., Ireland H., Crowhurst R., Luo Z., Watson A., Foster T., Gapper N., Giovanonni J., Mattheis J., Watkins C. (2014). Selection of low-variance expressed *Malus x domestica* (apple) genes for use as quantitative PCR reference genes (housekeepers). Tree Genet. Genomes.

[87] Zhou Z., Cong P., Tian Y., Zhu Y. (2017). Using RNA-seq data to select reference genes for normalizing gene expression in apple roots. PLoS ONE.

[88] Kucheryayskiy S. (2020). mdatools—R package for chemometrics. Chemom. Intell. Lab. Syst..

[89] Warnes G.R. Gplots: Various R Programming Tools for Plotting Data. https://CRAN.R-project.org/package=gplots.

[90] Oksanen J., Simpson G., Blanchet F., Kindt R., Legendre P., Minchin P., O’Hara R., Solymos P., Stevens M., Szoecs E. Vegan: Community Ecology Package. R Package Version 2.6-8. https://CRAN.R-project.org/package=vegan.

[91] Kassambara A. Rstatix: Pipe-Friendly Framework for Basic Statistical Tests. R Package Version 0.7.2. https://CRAN.R-project.org/package=rstatix.

[92] Wickham H. (2016). Ggplot2: Elegant Graphics for Data Analysis.

