# Supplementary material for: The Identification of Transcriptomic and Phytohormonal Biomarkers for Monitoring Drought and Evaluating the Potential of Acibenzolar-S-Methyl Root Application to Prime Two Apple Rootstock Genotypes for Drought Resistance
Source: Int J Mol Sci. 2025 Jul 21;26(14):6986. doi: 10.3390/ijms26146986 (PMC12295346; doi:10.3390/ijms26146986)
Supplement: Supplementary file 1 [file ijms-26-06986-s001.zip › Apple drought paper Supplementary Section.pdf]

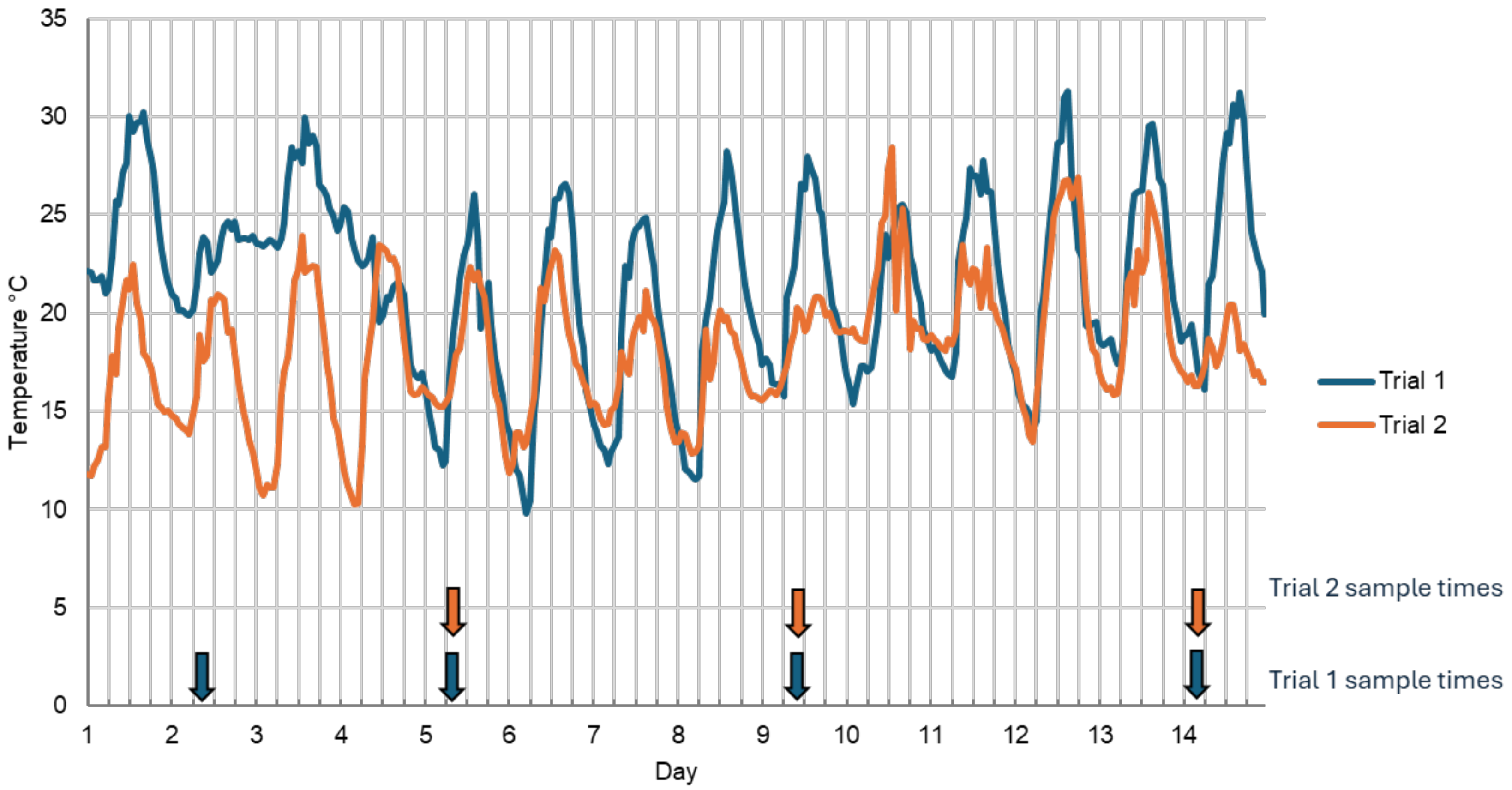

Supplementary Figure S1: Temperature data and respective sample time points (arrows) in Trial 1 and Trial 2. Tissue samples were collected, and physiology measurements were recorded between 10:00 am and noon each day.

A) Roots	CG202				M9			
	**2**	**5**	**9**	**14**	**2**	**5**	**9**	**14**
ABI4_i	0.90	0.71	0.77	0.70	0.80	1.06	0.50	0.77
ABI4_ii	1.53	0.76	1.21	0.76	1.14	**0.42**	0.71	0.80
ARF17_i	0.90	**0.71**	0.84	0.96	1.10	1.03	1.02	1.11
CAS	0.89	**1.59**	1.07	0.98	1.26	0.79	0.70	0.74
CER2_i	1.55	1.02	0.69	1.26	0.75	2.23	0.57	1.32
CER2_ii	1.17	1.35	1.48	0.98	**1.80**	0.89	**2.37**	0.56
DREB2C	0.65	**0.40**	0.88	1.22	**0.52**	0.62	0.77	1.24
ERD15_i	**1.26**	0.60	1.15	**1.26**	1.02	0.80	**1.46**	**1.52**
ERD15_ii	1.15	0.88	**1.42**	**2.04**	1.04	0.80	**1.66**	**2.37**
ERF053_i	**1.75**	1.30	**4.72**	**2.07**	**1.79**	**1.80**	**5.47**	**3.63**
ERF053_ii	1.47	1.19	**1.52**	1.34	1.21	0.75	**2.07**	**2.14**
FKBP_i	0.97	0.55	0.54	**0.33**	0.97	0.75	0.83	**0.40**
FKBP_ii	0.99	0.89	0.73	**0.56**	1.01	1.17	0.82	**0.62**
HY5	0.95	0.90	0.90	0.93	1.10	0.65	1.01	0.83
MYB68	**0.30**	**0.31**	**0.10**	**0.09**	**0.26**	**0.37**	**0.10**	**0.23**
MYB88_MYB124	0.72	1.74	1.00	**0.60**	1.24	0.85	1.02	0.51
PYL9_i	**0.86**	**0.76**	**0.72**	**0.66**	0.97	**0.72**	**0.81**	**0.67**
PYL9_ii	**0.84**	**0.98**	**0.74**	**0.67**	0.91	1.01	**0.64**	**0.62**
PYL9_iii	**1.20**	**1.47**	**0.67**	**0.63**	0.98	0.93	**0.57**	**0.53**
RAP2_4_i	**0.52**	0.65	**0.37**	**0.51**	1.04	0.83	0.92	**0.38**
RAP2_4_ii	1.08	**0.78**	0.98	1.07	1.05	0.83	**1.47**	1.06
RD29B_ii	1.75	0.77	1.62	1.01	0.69	0.67	0.67	0.66
SAT1	1.06	0.99	**1.33**	1.26	1.25	0.88	1.25	0.97
SERRATE	0.92	0.88	0.99	0.99	0.90	1.07	0.90	0.95
TIP1_1	0.97	1.00	**1.48**	1.35	1.37	1.15	**2.17**	**1.52**
TIP4	1.12	1.07	**0.23**	**0.32**	1.19	0.79	**0.38**	**0.38**
VHA_B_i	0.96	0.92	0.94	1.12	0.92	**0.76**	**0.87**	0.93
VHA_B_ii	0.96	0.95	0.94	1.14	0.94	**0.85**	1.03	1.04
WRI4_i	**1.59**	**1.64**	**1.58**	**2.04**	**1.55**	1.30	**1.90**	**2.39**
WRI4_ii	10.74	6.17	0.93	0.13	1.17	0.53	0.32	0.49
WRKY40	0.86	1.24	0.92	**0.39**	0.62	0.78	0.74	**0.51**

B) Leaves	CG202				M9			
	**2**	**5**	**9**	**14**	**2**	**5**	**9**	**14**
ABI4_i	**0.44**	0.84	**0.32**	1.67	1.79	1.43	1.15	0.89
ABI4_ii	1.36	**2.02**	0.63	0.71	1.76	0.76	1.08	0.87
ARF17_i	0.86	1.23	0.69	**0.64**	0.97	1.10	0.89	**0.26**
CAS	1.17	0.98	0.94	**0.67**	0.95	1.03	1.30	**0.21**
CER2_i	0.94	0.92	1.05	**3.71**	1.61	1.27	1.11	**4.22**
CER2_ii	1.03	0.80	0.90	1.04	1.13	1.80	1.06	**0.39**
CYP707A1_i	0.99	1.15	**0.32**	0.99	**4.22**	1.03	0.83	1.68
CYP707A1_ii	1.09	0.87	**0.61**	1.05	1.05	1.17	1.00	0.96
CYP707A2_i	0.98	0.85	**0.50**	1.26	**1.74**	**1.77**	0.73	1.50
DREB2C	0.76	0.90	1.04	0.81	1.18	**1.61**	1.06	0.47
ERD15_i	0.88	1.10	**0.81**	0.86	1.19	1.18	0.83	0.90
ERD15_ii	0.91	0.81	1.33	**1.58**	**1.35**	1.00	0.87	**1.45**
ERF053_i	0.69	0.80	**1.56**	1.03	**1.72**	1.04	1.26	**2.17**
ERF053_ii	0.82	0.78	1.19	1.09	1.14	1.10	0.96	**2.22**
FKBP_i	0.76	1.44	1.18	**2.01**	1.11	**1.81**	0.73	1.03
FKBP_ii	1.00	1.22	0.81	1.11	1.12	1.10	1.02	1.02
HY5	0.89	0.90	0.81	0.88	1.10	0.93	0.88	0.81
MYB68	0.89	0.56	**0.25**	0.69	1.03	1.49	**2.49**	**0.18**
MYB88_MYB124	1.15	0.88	0.81	**0.61**	1.21	1.06	0.79	**0.26**
NCED3_i	1.00	0.70	1.35	0.45	1.15	0.54	1.04	**0.41**
NCED3_ii	0.84	**0.54**	**0.51**	**0.24**	0.97	1.36	1.30	**0.52**
PYL9_i	0.89	**0.86**	**0.87**	**0.68**	1.10	**1.52**	**0.79**	**0.57**
PYL9_ii	1.02	**0.84**	0.90	**0.65**	0.87	1.15	1.20	**0.57**
PYL9_iii	1.00	0.94	**0.80**	**0.81**	**0.81**	1.17	1.08	**0.51**
RAP2_4_i	0.86	0.87	0.61	**0.11**	0.94	0.95	0.92	**0.02**
RAP2_4_ii	0.87	0.94	0.95	**0.60**	1.04	1.04	**0.73**	**0.63**
RD29B_ii	0.71	1.29	1.50	0.85	1.30	**2.19**	0.69	0.47
SAT1	0.97	0.95	0.67	**1.43**	**1.24**	1.11	1.07	**2.09**
SERRATE	0.94	1.03	1.06	1.02	0.96	**1.15**	**0.83**	**0.82**
TIP1_1	0.90	0.93	1.05	**0.44**	1.22	0.98	**0.46**	**0.05**
TIP4	0.93	1.35	0.49	1.16	1.34	0.91	0.75	1.57
VHA_B_i	**0.82**	0.90	0.96	**0.69**	1.13	**1.29**	0.89	**0.54**
VHA_B_ii	0.92	**0.85**	0.94	**0.60**	1.05	1.08	**0.72**	**0.50**
WRI4_i	1.51	0.68	1.61	**2.28**	0.99	1.40	0.58	0.95
WRI4_ii	0.64	0.55	1.05	1.28	1.07	0.79	**0.51**	0.70
WRKY40	0.70	0.74	**0.58**	**0.24**	1.54	1.24	0.67	**0.24**

Supplementary Figure S2: Heat map presenting fold-change gene expression data in **A)** root, and **B)** leaf tissue, induced by drought stress conditions in *Malus domestica* CG202 and M9 rootstocks after 2, 5, 9, and 14 days without water, in Trial 1. Control irrigated plants received a total volume of 1.8 L water/day via drippers There were five replicate plants/sample time/treatment/rootstock. Gene expression was quantified by PlexSet® NanoString using *CKB4*, *FYPP3* and *GPAT1* as reference genes. Genes of interest are listed in the left-hand column. Numeric values give the fold changes relative to irrigated plants, sampled at the same time periods. Red colouration indicates a fold-increase, blue a fold-decrease, and white is no fold change relative to the control. Colour intensity is indicative of the degree of change, but all fold increases greater than 10 have the same red colour intensity. Statistically significant differences from the control plants at each timepoint, as determined by AVOVA ($p \leq 0.05$) analysis, are indicated in bold typeface.

Root	CG202									M9								
	Dry			ASM			ASM+Dry			Dry			ASM			ASM+Dry		
	**5**	**9**	**14**	**5**	**9**	**14**	**5**	**9**	**14**	**5**	**9**	**14**	**5**	**9**	**14**	**5**	**9**	**14**
ABI4_i	**0.22**	0.40	0.65	**0.09**	**0.14**	0.39	**0.15**	**0.32**	0.75	0.88	0.88	1.11	0.86	0.81	0.83	0.63	0.88	2.25
ABI4_ii	1.24	1.03	1.01	0.96	1.18	1.17	1.31	1.10	1.40	0.70	1.29	0.74	0.59	1.22	0.88	0.84	0.97	0.60
ARF17	0.92	0.97	1.19	0.92	0.76	1.02	1.00	0.86	**1.56**	0.86	1.03	1.33	0.93	1.01	1.15	0.86	1.07	1.19
ATG8i	0.99	0.94	0.90	1.10	1.01	1.01	**1.18**	1.05	0.97	1.01	1.03	0.98	**0.82**	0.96	0.93	0.88	0.91	1.06
BES1/BZR2	1.02	1.04	0.81	1.05	1.08	1.08	1.12	0.87	0.72	0.93	0.98	0.80	0.76	1.07	0.86	0.79	0.84	0.96
CAS	1.28	1.16	1.03	0.77	0.77	0.92	1.29	1.13	0.67	1.10	1.14	0.87	1.06	1.19	0.91	1.34	1.53	1.02
CER2_i	**2.57**	2.20	2.21	2.00	1.14	1.00	2.33	1.53	0.99	0.53	1.40	1.28	0.79	1.59	0.81	0.82	0.71	0.91
CER2_ii	1.34	1.45	1.48	0.96	1.04	1.00	**1.86**	**1.52**	1.53	**1.93**	**1.72**	**1.71**	1.28	0.96	0.93	**1.89**	1.36	1.34
CYP707A1_i	1.20	**0.43**	**0.42**	1.42	0.90	1.03	0.75	**0.41**	**0.51**	**0.61**	**0.59**	**0.34**	0.95	1.16	0.90	0.67	**0.71**	**0.47**
DREB2C	0.90	0.74	0.70	0.91	0.80	0.97	**0.53**	0.63	1.01	0.84	0.99	**0.27**	0.75	1.15	0.71	1.33	1.49	0.53
ERD15_ii	1.25	0.91	1.31	1.42	0.88	1.16	1.09	1.00	1.21	1.10	0.93	1.00	1.04	1.06	0.91	0.99	1.21	1.06
ERF053_i	1.48	1.48	**1.95**	1.02	0.74	1.28	**1.88**	**1.72**	1.49	**1.78**	**1.84**	**2.69**	1.16	0.79	1.02	1.34	1.47	**2.09**
ERF053_ii	1.82	0.97	**1.94**	1.24	0.70	1.29	1.44	1.51	**1.82**	1.21	0.99	1.49	1.05	1.00	1.20	1.02	1.23	1.60
FKBP_i	0.75	1.19	**1.97**	0.98	0.71	0.56	0.80	0.83	**1.95**	0.69	0.86	0.79	0.74	0.85	1.03	0.78	0.91	0.79
MADS_box_AGL16_i	0.97	1.03	0.92	1.03	0.97	1.22	1.10	1.13	1.18	1.09	0.93	0.77	0.77	0.69	0.87	0.82	0.78	0.82
MADS_box_AGL16_ii	0.77	0.83	1.07	0.98	0.65	0.89	1.04	0.78	1.16	0.72	1.12	0.75	0.65	1.08	1.30	0.74	1.08	1.01
MdNAC1	0.96	**1.73**	1.18	0.81	1.16	**0.67**	1.41	**1.62**	0.69	1.07	1.20	1.03	1.03	0.97	1.07	1.33	**1.34**	1.19
MdNAC143	1.37	1.09	1.91	1.41	0.87	1.28	1.41	1.29	**1.96**	1.13	1.14	0.77	0.93	1.14	0.83	1.14	**1.43**	1.06
MYB68	**0.18**	**0.14**	**0.18**	1.50	0.89	1.37	**0.33**	**0.17**	**0.19**	**0.44**	0.67	0.47	**0.45**	0.81	0.64	**0.45**	**0.40**	**0.42**
MYB88_MYB124	1.03	1.19	0.85	0.93	1.10	1.07	0.97	0.96	0.91	1.16	1.01	0.98	1.20	0.94	0.88	1.01	0.93	0.96
OST1_SnRK_2_6	0.91	**0.78**	**0.69**	**1.22**	1.13	1.10	1.04	0.84	**0.72**	0.85	0.86	**0.82**	0.84	0.94	1.06	0.85	0.88	0.84
RAP2_4_i	1.27	**0.51**	0.63	1.21	0.96	1.13	1.14	0.96	0.79	0.63	0.68	**0.55**	0.74	1.00	0.82	**0.56**	0.60	**0.59**
RD29B_ii	1.66	1.45	0.93	0.72	1.05	0.56	1.59	1.33	0.97	0.72	1.30	0.74	0.67	1.21	0.73	0.85	0.87	1.30
SnRK_2_3	0.98	0.81	**0.67**	1.18	1.01	1.04	1.03	0.90	**0.65**	0.99	0.82	**0.67**	0.80	0.84	0.81	0.80	**0.79**	**0.72**
SnRK_2_8	1.01	0.82	**0.76**	1.06	0.97	1.12	0.86	**0.75**	**0.64**	**0.76**	**0.74**	**0.71**	0.91	0.85	0.92	**0.74**	**0.67**	**0.78**
TIP1_1	1.31	1.16	1.34	0.80	0.89	1.09	1.01	0.88	**1.61**	1.40	**1.45**	1.37	**1.49**	1.21	0.97	1.26	1.09	1.26
TIP4	0.80	0.69	0.52	1.44	1.13	1.08	1.03	0.83	**0.62**	0.98	0.74	**0.56**	0.74	0.77	0.83	0.77	**0.60**	**0.51**
WRI4_i	0.82	1.07	**1.58**	0.72	0.71	1.08	0.99	1.06	**2.26**	**1.82**	**1.81**	1.31	1.07	0.94	0.82	1.43	1.37	1.22
WRI4_ii	0.64	0.75	1.12	0.43	0.49	0.63	0.69	1.06	1.63	0.71	1.20	1.44	1.19	1.91	1.60	1.23	1.51	1.27
WRKY40	1.32	0.52	0.71	**2.30**	0.85	1.28	0.98	0.54	0.72	0.94	0.70	**0.43**	1.34	1.21	0.88	0.75	1.10	0.67

Supplementary Figure S3: Heat map presenting fold-change in gene expression data in roots, induced by drought stress conditions and acibenzolar-S-methyl (ASM) application in *Malus domestica* GG202 and M9 rootstocks after 5, 9, and 14 days without water, in Trial 2. ASM (10 mg a.i./plant) was applied as a root drench, both 14 days before the start of experiment and again on day 0, immediately before dripper removal. Control irrigated plants received a total volume of 1.8 L water/day. There were three replicate plants/sample time/treatment/rootstock. Gene expression was quantified by PlexSet® NanoString using *CKB4*, *FYPP3*, *GPAT1*, *LTL1*, and *Protein GRIP* as reference genes. All fold changes (numeric values in the figure) are relative to irrigated plants at the same time. Red colouration indicates a fold-increase, blue a fold-decrease, and white is no fold change relative to the control. Statistically significant differences from the control as each timepoint, as determined by ANOVA ($p \leq 0.05$) analysis, are indicated in bold typeface.

Leaf	CG202									M9								
	Dry			ASM			ASM+Dry			Dry			ASM			ASM+Dry		
	5	9	14	5	9	14	5	9	14	5	9	14	5	9	14	5	9	14
ABI4_i	0.81	0.65	**0.47**	0.79	0.85	0.80	0.59	**0.42**	0.54	0.99	1.51	0.69	0.79	0.97	0.82	**4.49**	**3.24**	**2.62**
ABI4_ii	1.33	1.35	1.04	1.77	**2.23**	1.67	1.57	**1.91**	**1.88**	0.75	0.77	0.88	0.78	0.86	1.21	0.56	0.72	0.89
ARF17	**0.63**	0.90	1.13	0.90	0.74	0.84	0.72	0.85	1.01	1.03	**1.56**	1.14	**0.55**	1.43	0.82	1.21	1.46	1.10
ATG8i	1.04	**1.22**	**1.30**	1.05	0.99	1.06	**1.21**	**1.19**	**1.37**	0.87	1.06	1.09	**0.83**	0.90	**0.83**	0.89	0.89	1.09
BES1/BZR2	1.07	1.02	**0.61**	**0.71**	0.85	0.86	0.85	0.79	**0.51**	0.83	**0.74**	**0.69**	0.93	0.86	1.03	0.95	**0.75**	**0.72**
CAS	1.13	0.84	**0.56**	**1.23**	0.95	0.89	1.03	**0.75**	**0.50**	1.01	1.08	0.91	1.01	0.98	0.95	0.97	1.01	0.86
CER2_i	1.19	1.29	**0.61**	1.05	**1.91**	1.28	0.99	0.89	**0.48**	1.00	0.74	1.15	1.13	0.78	1.10	0.93	0.81	0.93
CER2_ii	1.06	**1.24**	1.09	0.91	1.06	1.01	1.03	1.13	1.01	1.00	**0.82**	1.13	1.13	0.86	1.01	1.07	**0.84**	0.97
CYP707A1_i	0.81	0.71	1.00	0.83	**0.44**	0.84	0.84	1.59	1.48	1.00	1.51	1.56	0.79	1.42	1.23	1.00	1.44	1.18
CYP707A1_ii	1.29	0.87	1.24	1.21	**0.65**	0.75	1.00	0.92	1.26	1.19	1.08	0.97	1.18	1.06	0.97	1.10	1.08	1.08
CYP707A2	0.74	1.14	1.45	1.04	0.99	0.77	0.84	0.92	1.45	1.21	0.99	**2.25**	1.07	1.01	1.34	1.29	1.02	**2.04**
DREB2C	**0.59**	0.69	1.15	0.70	0.76	0.81	0.70	0.91	1.25	**0.59**	0.80	0.76	0.96	0.79	0.83	0.73	0.96	1.17
ERD15_ii	1.22	1.17	1.24	1.05	1.26	1.21	0.98	1.10	1.35	0.95	0.97	0.94	0.96	1.03	0.99	0.86	1.00	0.77
ERF053_i	1.22	1.35	**1.62**	0.85	1.06	0.86	0.87	**1.63**	**1.71**	1.07	1.23	1.11	0.85	1.07	0.93	0.90	1.07	0.73
ERF053_ii	1.01	1.07	**0.67**	0.79	0.89	0.73	0.87	0.97	**0.58**	0.83	1.04	0.73	0.95	0.87	0.85	1.09	1.07	**0.70**
FKBP_i	0.99	1.23	0.91	1.10	0.99	0.86	1.01	**1.23**	0.93	1.04	1.21	1.15	1.04	1.01	1.02	1.00	1.03	1.13
MADS_box_AGL16_i	1.22	1.30	1.20	1.14	1.10	1.27	1.45	1.31	1.21	**0.51**	**0.57**	**0.59**	**0.33**	**0.37**	**0.60**	**0.44**	**0.49**	**0.48**
MADS_box_AGL16_ii	0.71	0.89	0.72	1.46	**1.81**	1.57	**1.93**	1.60	1.45	1.52	1.51	0.68	0.82	1.12	0.80	0.71	0.68	0.61
MdbHLH130	0.96	1.17	**1.30**	0.95	1.08	1.00	1.04	**1.20**	**1.46**	0.91	1.01	**1.19**	0.91	1.00	1.04	0.94	0.93	1.13
MdNAC1	1.18	**1.59**	1.45	1.06	1.04	1.00	1.34	1.17	1.10	0.86	1.24	1.30	0.83	1.07	0.80	0.79	1.28	1.12
MdNAC143	0.85	1.66	1.61	0.95	1.23	1.11	0.87	1.46	1.79	0.78	1.12	0.56	0.76	1.55	0.79	0.60	1.25	0.71
MYB68	1.69	1.28	0.85	1.67	1.39	1.29	1.24	1.20	1.24	0.90	0.84	0.89	0.87	0.84	0.82	0.99	1.08	0.66
MYB88_MYB124	1.16	1.14	0.60	1.05	1.11	0.96	1.03	0.93	**0.59**	0.96	0.84	1.01	1.04	0.97	1.07	0.88	0.87	0.81
NCED3_i	**1.53**	1.13	**0.64**	0.88	0.96	1.08	1.07	1.30	**0.60**	1.05	**1.57**	1.26	0.78	1.23	0.90	1.02	1.50	1.25
NCED3_ii	1.23	0.67	**0.14**	0.93	0.87	0.98	0.90	**0.40**	**0.08**	1.31	0.80	0.70	0.98	0.98	1.10	0.98	0.78	**0.50**
OST1_SnRK_2_6	1.01	0.92	**0.83**	1.06	0.88	1.01	1.01	**0.85**	**0.72**	0.93	0.95	0.88	0.98	1.04	1.06	0.95	0.90	0.90
RAP2_4_i	0.70	0.81	**0.56**	1.01	0.98	0.80	0.79	0.79	**0.50**	1.02	0.80	0.78	0.87	1.21	0.89	0.83	0.80	0.79
RD29B_ii	1.05	0.79	0.51	1.12	1.30	0.62	0.66	0.68	0.51	0.66	1.06	1.17	0.72	0.78	1.19	0.73	0.86	1.04
SnRK_2_3	0.97	0.97	0.89	0.99	0.97	1.05	0.98	0.88	0.90	0.95	1.09	0.94	0.90	0.91	0.92	0.86	0.93	0.90
SnRK_2_8	0.99	0.99	0.88	1.02	1.00	1.06	0.97	0.94	**0.80**	1.00	0.99	0.93	0.90	0.93	0.96	0.94	0.95	0.99
TIP1_1	1.06	0.72	**0.47**	0.98	1.14	0.92	0.75	0.66	**0.36**	1.01	**0.62**	0.68	1.04	0.93	1.01	1.01	0.67	**0.54**
TIP4	0.59	0.74	0.96	0.88	1.15	1.32	0.79	1.36	1.35	**0.50**	**0.46**	**0.51**	**0.38**	**0.45**	**0.57**	**0.33**	**0.41**	**0.24**
WRI4_i	0.60	1.14	0.77	1.66	1.53	1.32	1.31	1.42	1.26	1.48	**2.23**	1.05	0.91	0.74	0.54	1.19	0.50	0.80
WRI4_ii	0.64	0.94	0.59	0.94	0.96	0.84	0.72	1.35	0.78	1.51	1.41	0.90	0.58	0.97	1.30	0.55	0.86	0.61
WRKY40	0.92	0.87	0.71	1.39	1.07	1.46	0.99	0.81	0.75	0.89	1.16	**0.46**	0.68	1.49	0.70	0.71	1.02	**0.40**

Supplementary Figure S4: Heat map presenting fold-change in gene expression data in leaves, induced by drought stress conditions and acibenzolar-S-methyl (ASM) application in *Malus domestica* GG202 and M9 rootstocks after 5, 9, and 14 days without water, in Trial 2. ASM (10 mg a.i./plant) was applied as a root drench, both 14 days before the start of experiment and again on day 0, immediately before dripper removal. Control irrigated plants received a total volume of 1.8 L water/day. There were three replicate plants/sample time/treatment/rootstock. Gene expression was quantified by PlexSet® NanoString using *CKB4*, *FYPP3*, *GPAT1*, *LTL1*, and *Protein GRIP* as reference genes. All fold changes (numeric values in the figure) are relative to irrigated plants at the same time. Red colouration indicates a fold-increase, blue a fold-decrease, and white is no fold change relative to the control. Statistically significant differences from the control at each timepoint, as determined by ANOVA ($p \leq 0.05$) analysis, are indicated in bold typeface.

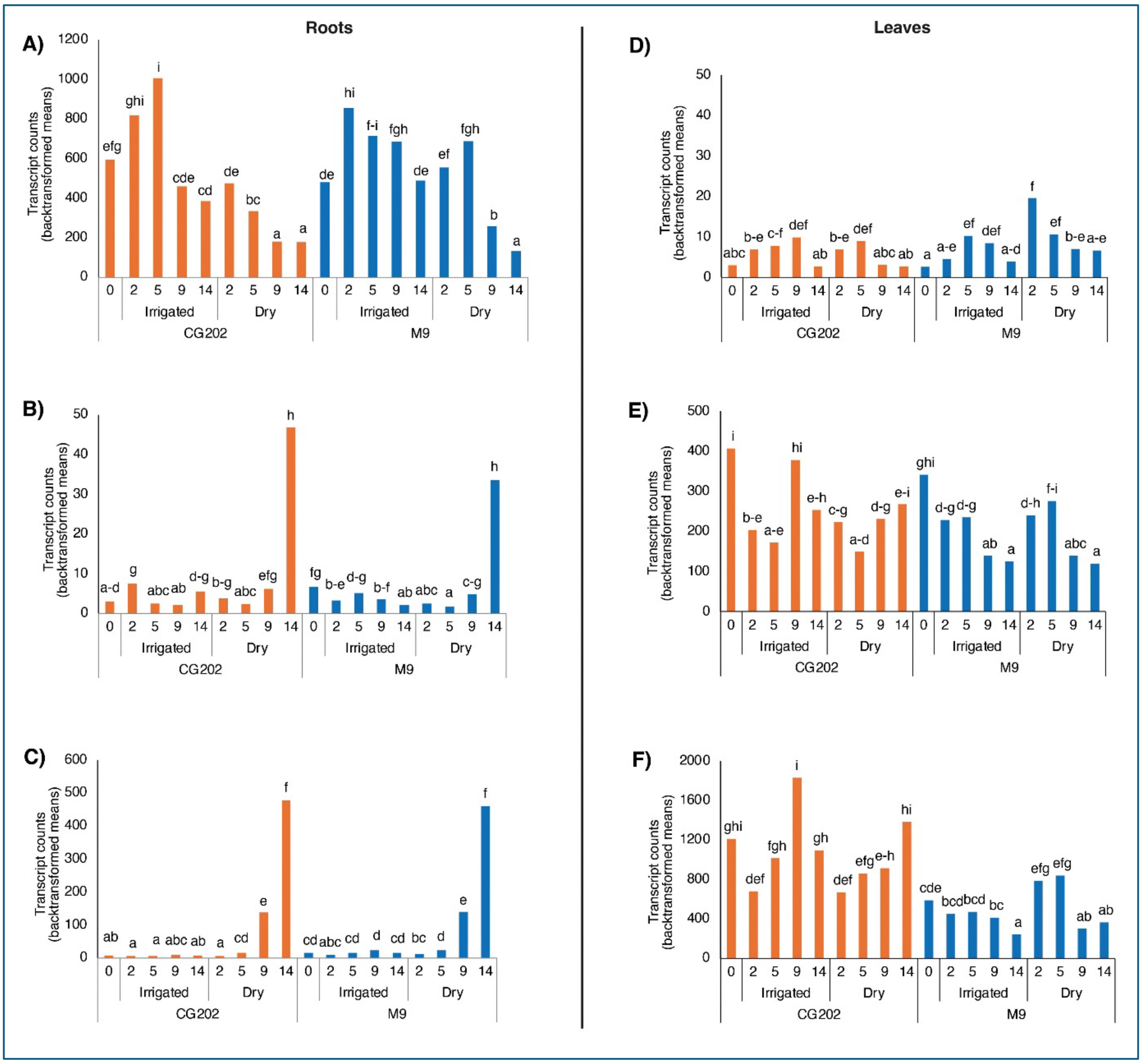

Supplementary Figure S5: Transcript counts (backtransformed $\log_2$ gene counts) as measured by PlexSet® NanoString in roots (A-C) and leaves (D-E) in CG202 and M9 *Malus domestica* rootstocks after 2, 5, 9 and 14 days without water, in Trial 1. Control irrigated plants received a total volume of 1.8 L water/day via drippers. Measurements taken on day 0 represent the baseline level of gene expression for plants from both treatment groups. There were five replicate plants/sample time/treatment/rootstock. CKB4, FYPP3 and GPAT1 were used as reference genes. The genes presented are A&D) *CYP707A1_i*; B&E) *CYP707A1_ii* C&F) *CYP707A2_i*. Different lettering over bars indicates statistically significant differences, as shown by Fisher's Least Significant Difference (LSD), $p \leq 0.05$, for each gene.

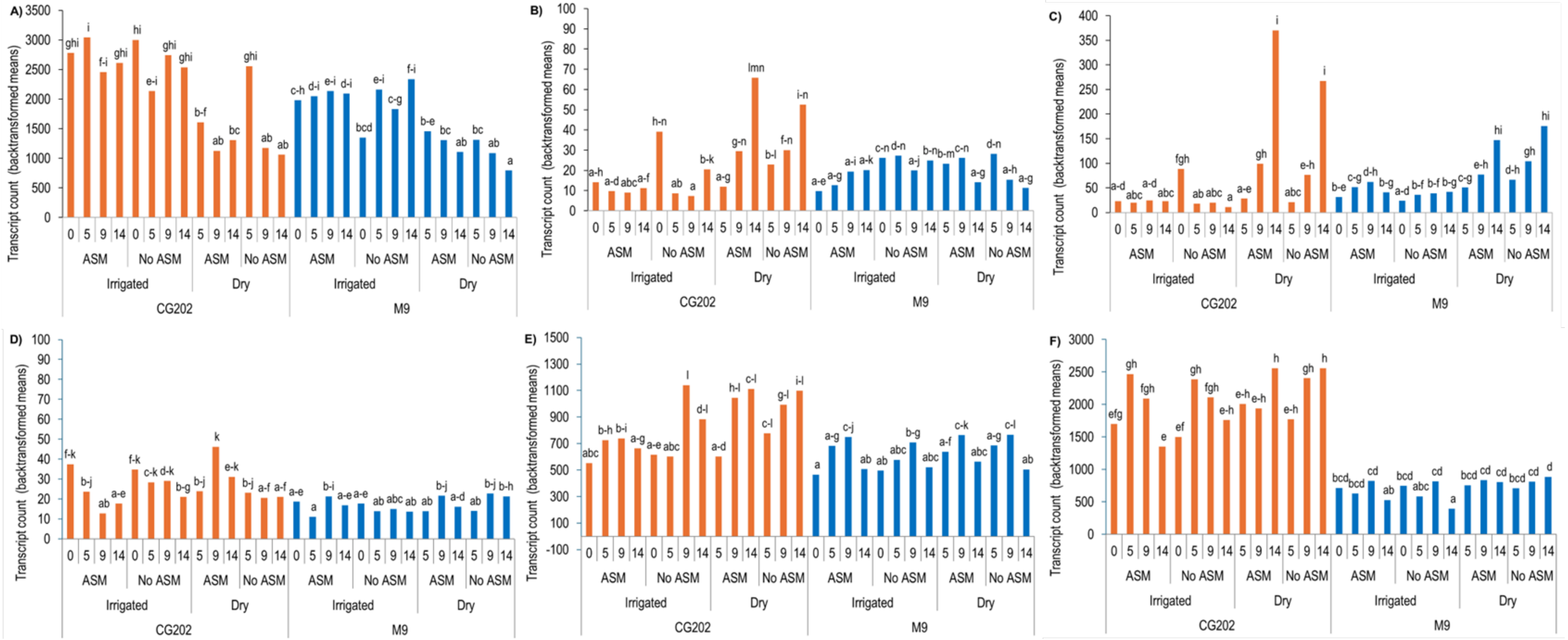

Supplementary Figure S6: Transcript counts (backtransformed $\log_2$ gene counts) as measured by PlexSet® NanoString in roots **(A-C)** and leaves **(D-F)** in CG202 and M9 *Malus domestica* rootstocks after 5, 9 and 14 days without water, in Trial 2. Control irrigated plants received a total volume of 1.8 L water/day via drippers. Acetyl-S-methyl (ASM), at 10 mg a.i./plant, was applied as a root drench, both 14 days before the start of experiment and again on day 0, immediately before dripper removal. There were three replicate plants/sample time/treatment/rootstock used for NanoString. *CKB4*, *FYPP3*, *GPAT1*, *LTL1*, and *Protein GRIP* were used as reference genes. The genes presented are **A&D)** *CYP707A1_i*; **B&E)** *CYP707A1_ii* **C&F)** *CYP707A2_i*. Different lettering over bars indicates statistically significant differences, as shown by Fisher's Least Significant Difference (LSD), $p \leq 0.05$, for each gene.

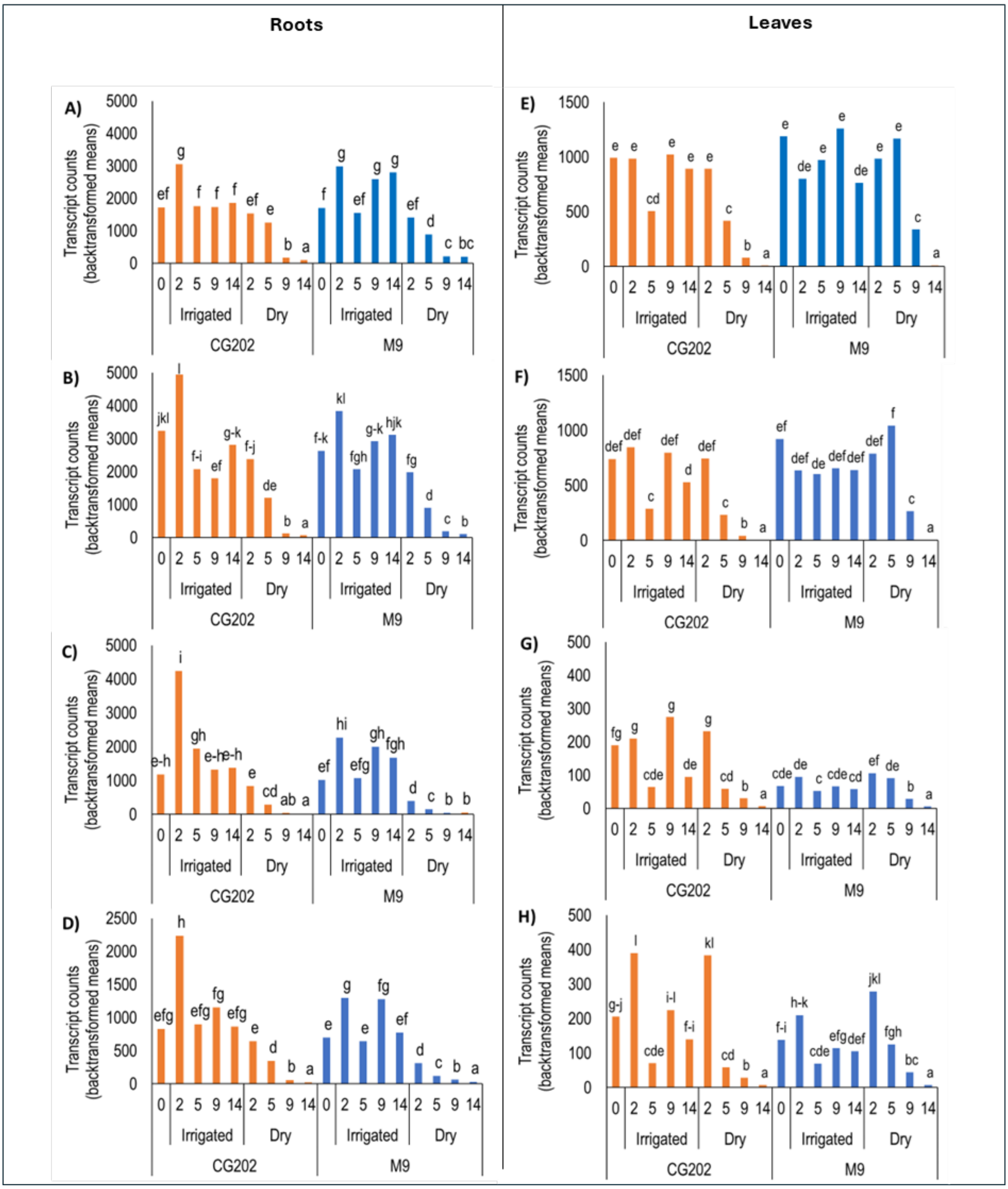

Supplementary Figure S7: Transcript counts (backtransformed log_2 gene counts) as measured by PlexSet® NanoString in roots (A-D) and leaves (E-H) in CG202 and M9 *Malus domestica* rootstocks after 2, 5, 9, and 14 days without water, in Trial 1. Control irrigated plants received a total volume of 1.8 L water/day via drippers. Measurements taken on day 0 represent the baseline level of gene expression for plants from both treatment groups. There were five replicate plants/sample time/treatment/rootstock. CKB4, FYPP3 and GPAT1 were used as reference genes. The genes presented are A&E) *PYL4_i*; B&F) *PYL4_ii*; C&G) *PYL4_iii*; and D&H) *PYL4_iv*. Different lettering over bars indicates statistically significant differences, as shown by Fisher's Least Significant Difference (LSD), $p \leq 0.05$, for each gene.

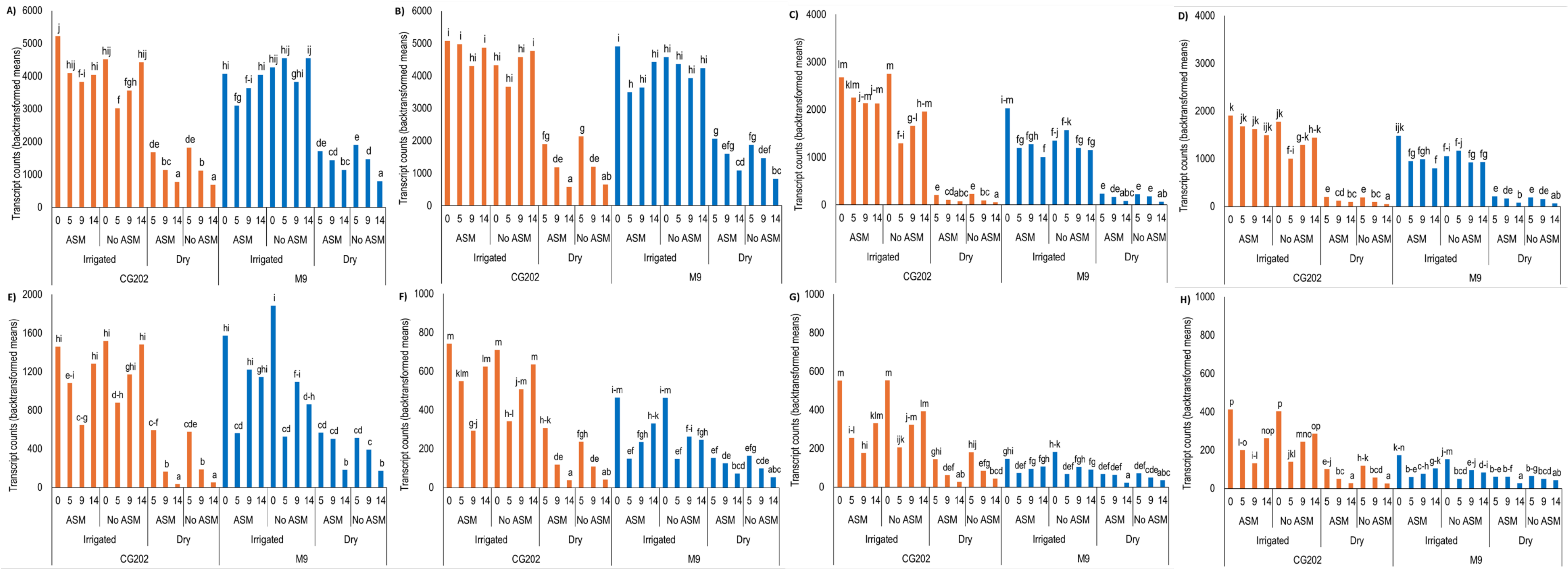

Supplementary Figure S8: Transcript counts (backtransformed $\log_2$ gene counts) as measured by PlexSet® NanoString in roots **(A-D)** and leaves **(E-H)** in CG202 and M9 *Malus domestica* rootstocks after 5, 9 and 14 days in irrigated and Dry plants, in Trial 2. Control irrigated plants received a total volume of 1.8 L water/day via drippers. Acibenzolar-S-methyl (ASM), at 10 mg a.i./plant, was applied as a root drench, both 14 days before the start of experiment and again on day 0, immediately before dripper removal. There were three replicate plants/sample time/treatment/rootstock. *CKB4*, *FYPP3*, *GPAT1*, *LTL1*, and *Protein GRIP* were used as reference genes. The genes presented are **A&E)** *PYL4_i*; **B&F)** *PYL4_ii* **C&G)** *PYL4_iii*; **D&H)** *PYL4_iv*. Different lettering over bars indicates statistically significant differences, as shown by Fisher's Least Significant Difference (LSD), $p \leq 0.05$, for each gene.

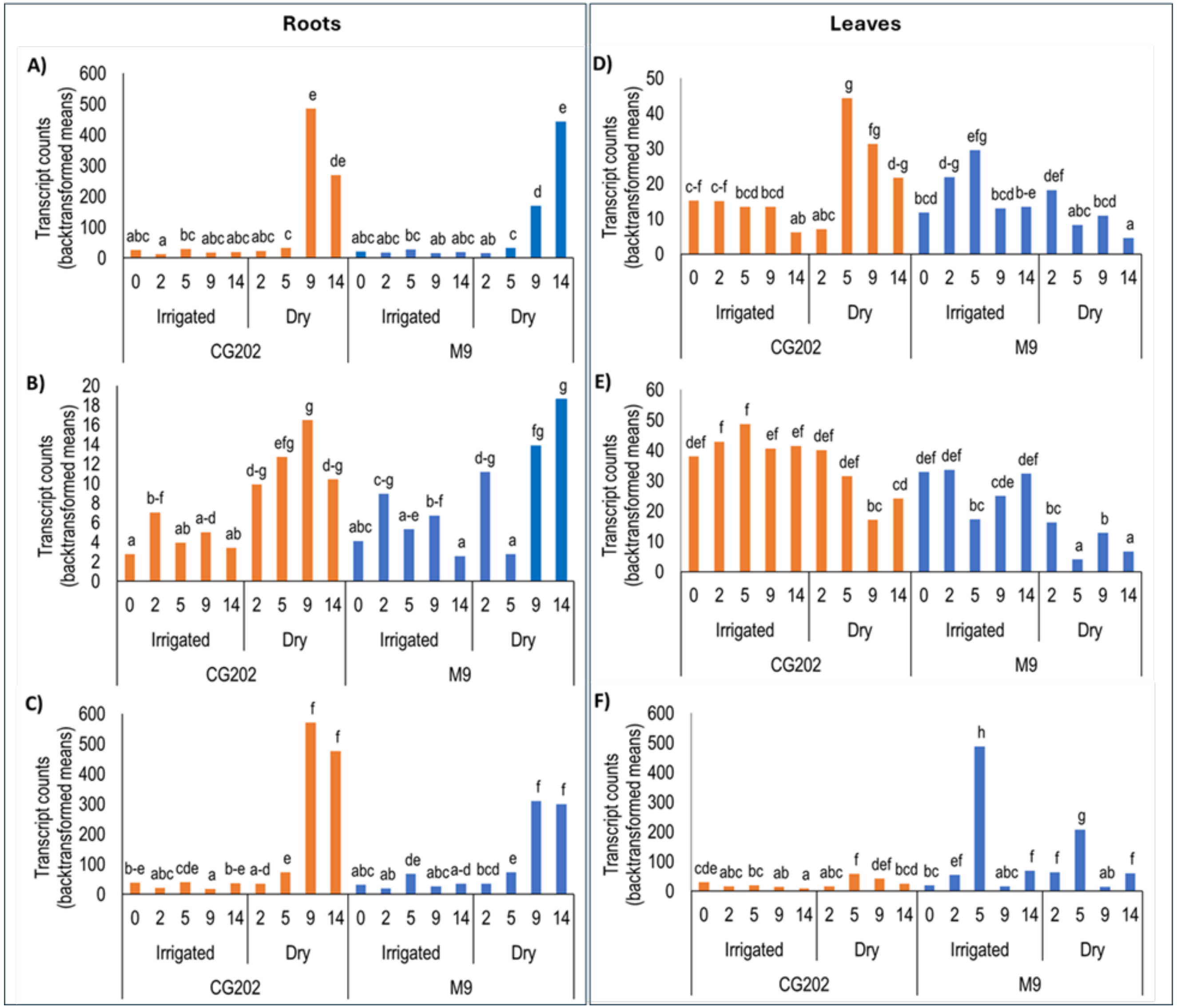

Supplementary Figure S9: Transcript counts (backtransformed $\log_2$ gene counts) as measured by PlexSet® NanoString in roots **(A-C)** and leaves **(D-F)** in CG202 and M9 *Malus domestica* rootstocks after 0, 2, 5, 9 and 14 days in irrigated and Dry plants, in Trial 1. Control irrigated plants received a total volume of 1.8 L water/day via drippers. There were five replicate plants/sample time/treatment/rootstock used for NanoString. *CKB4*, *FYPP3* and *GPAT1* were used as reference genes. The genes presented are **A&D)** *NCED3_i*; **B&E)** *NCED3_ii;* **C&F)** *NCED3_iii*. Different lettering over bars indicates statistically significant differences, as shown by Fisher's Least Significant Difference (LSD), $p \leq 0.05$, for each gene.

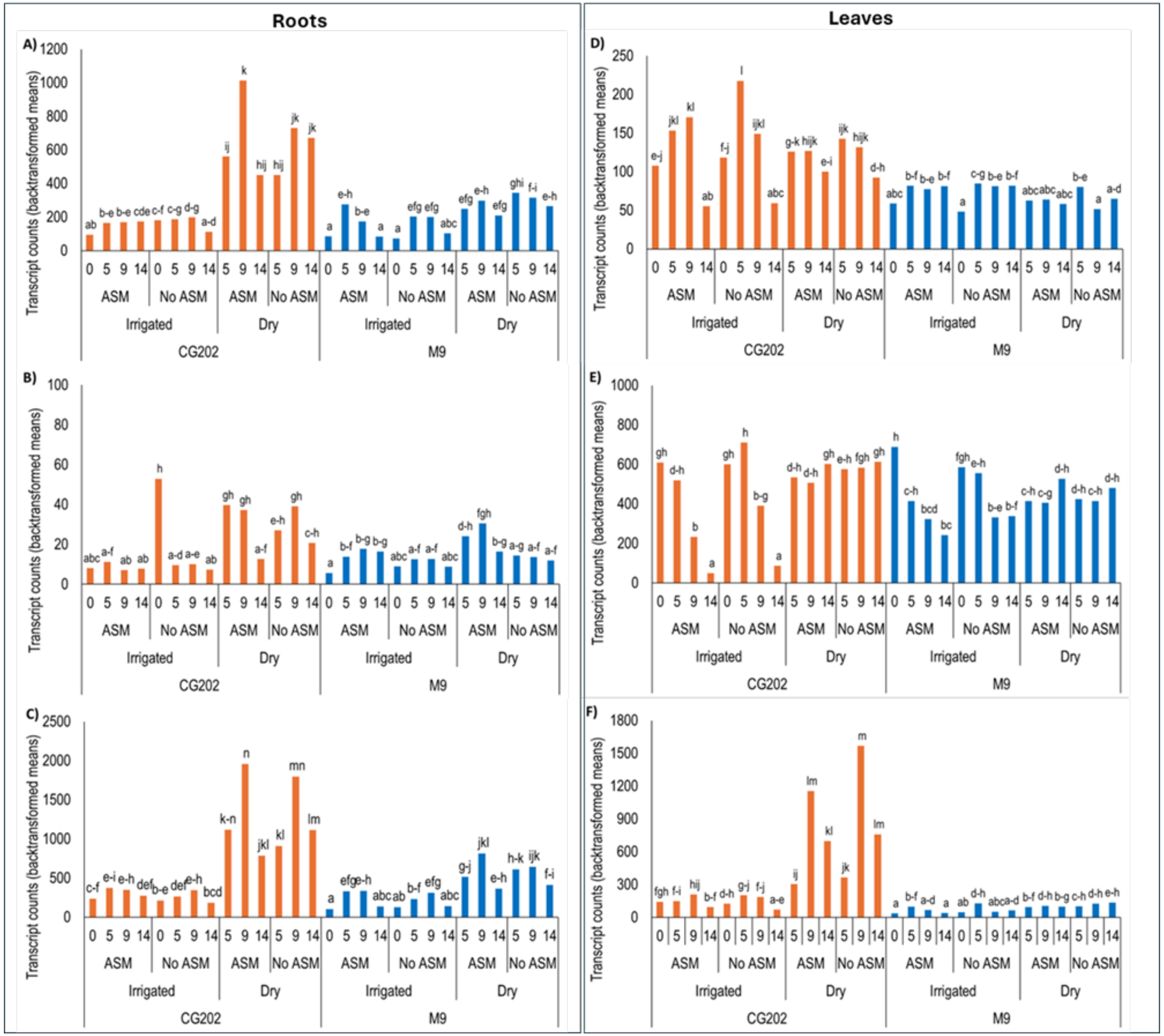

Supplementary Figure S10: Transcript counts (backtransformed log_2 gene counts) as measured by PlexSet® NanoString in roots **(A-C)** and leaves **(D-F)** in CG202 and M9 *Malus domestica* rootstocks after 0, 5, 9 and 14 days in irrigated and Dry plants, in Trial 2. Control irrigated plants received a total volume of 1.8 L water/day via drippers. Acibenzolar-S-methyl (ASM), at 10 mg a.i./plant, was applied as a root drench, both 14 days before the start of experiment and again on day 0, immediately before dripper removal. There were three replicate plants/sample time/treatment/rootstock. *CKB4*, *FYPP3*, *GPAT1*, *LTL1*, and *Protein GRIP* were used as reference genes. The genes presented are **A&D)** *NCED3_i*; **B&E)** *NCED3_ii;* **C&F)** *NCED3_iii*. Different lettering over bars indicates statistically significant differences, as shown by Fisher's Least Significant

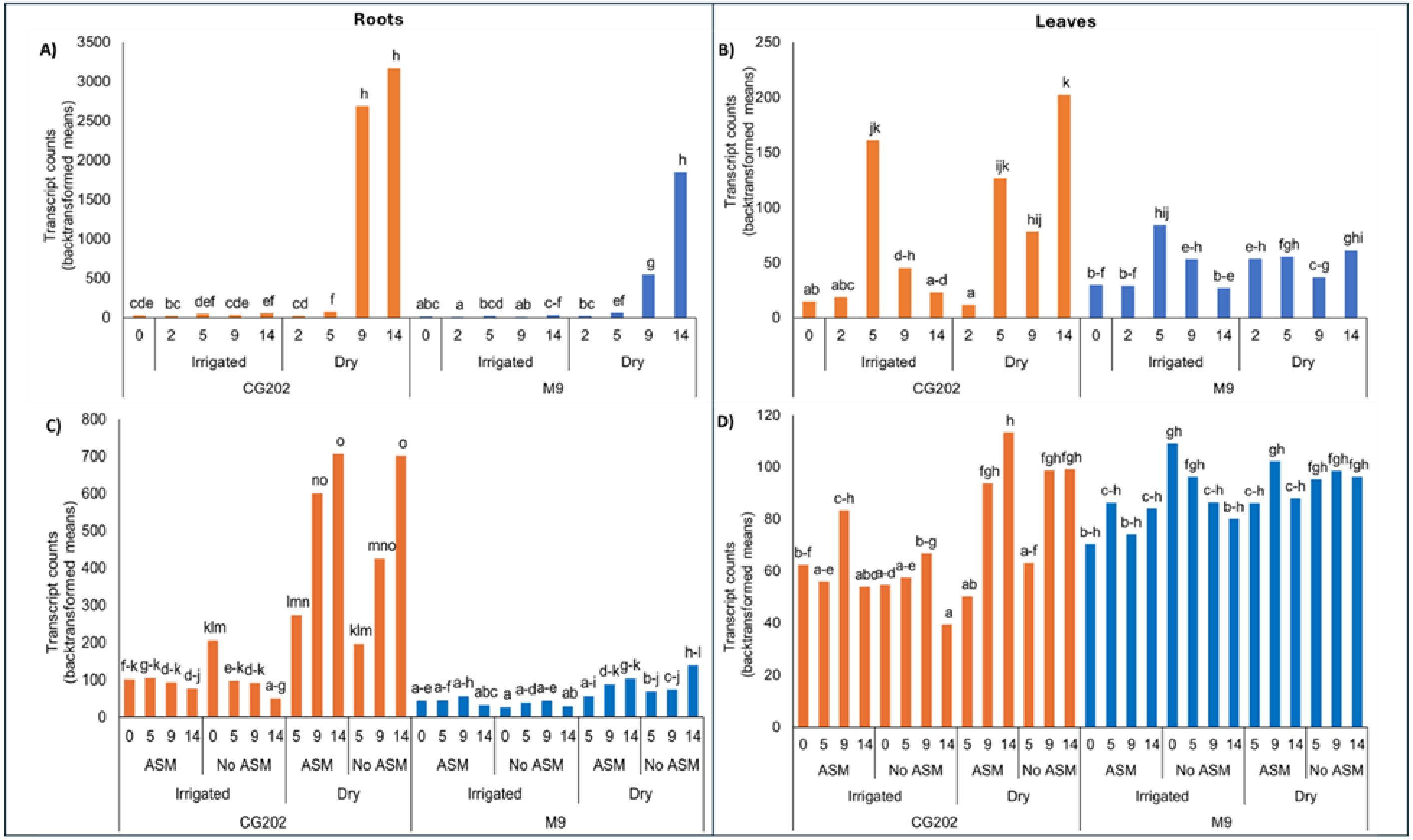

Supplementary Figure S11: Transcript counts (backtransformed log_2 gene counts) for *RD29B_i* as measured by PlexSet® NanoString in roots **(A&C)** and leaves **(B&D)** in CG202 and M9 *Malus domestica* rootstocks. Measurements were recorded after 0, 2, 5, 9 and 14 days in irrigated and Dry plants in Trial1 **(A&C)** and after 5, 9 and 14 days in Trial 2 **(B&D)**. Control irrigated plants received a total volume of 1.8 L water/day via drippers. In Trial 2, acibenzolar-S-methyl (ASM), at 10 mg a.i./plant, was applied as a root drench, at 14 days before the start of experiment and again on day 0, immediately before dripper removal. There were five replicate plants/sample time/treatment/rootstock in Trial 1 and three in Trial 2. *CKB4*, *FYPP3* and *GPAT1* were used as reference genes in Trial 1, and *CKB4*, *FYPP3*, *GPAT1*, *LTL1*, and *Protein GRIP* in Trial 2. Different lettering over bars indicates statistically significant differences, as shown by Fisher's Least Significant Difference (LSD), $p \leq 0.05$.

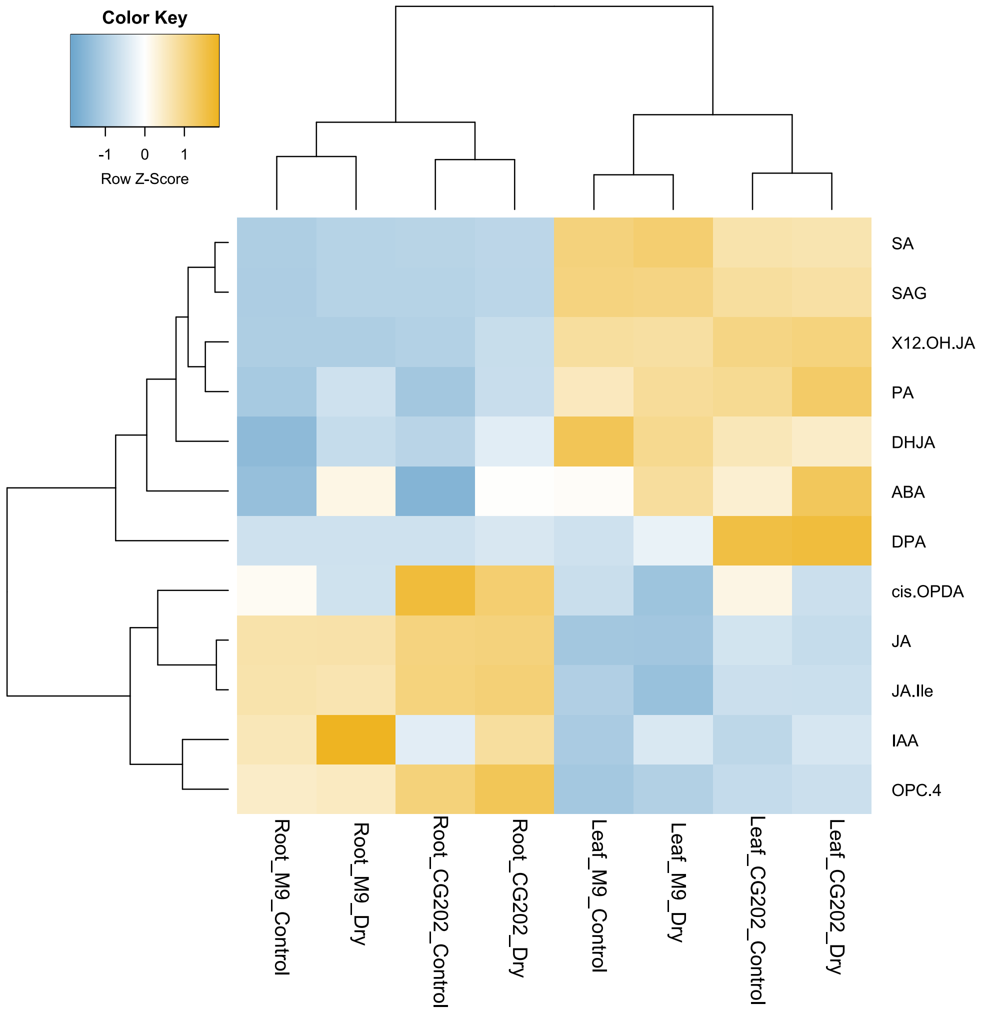

Supplementary Figure S12: Clustered Heatmap of phytohormone concentrations common to leaves and roots of two apple rootstock genotypes (CG202, M9) across common sampling timepoints (day 0, 2, 5, 9, 14) in Trial 1. Dry: drought-treated, Control: Irrigated. Data are scaled by row with yellow hues indicating positive and blue hues negative standard (Z-) scores, i.e., relative concentrations. **SA:** salicylic acid; **SAG:** salicylic acid glucoside; **IAA:** indole-3-acetic acid; **ABA**: abscisic acid; **PA**: phaseic acid; **DPA**: dihydrophaseic acid; **JA**: jasmonic acid; **JA-Ile**: jasmonic acid-isoleucine; **X12.OH.JA**: 12-hydroxyjasmonic acid; **DHJA**: dihydrojasmonic acid; **OPC-4**: 4-(3-oxo-2-(pent-2-en-1-yl)cyclopentyl)octanoic acid; **cis-OPDA**: 12-oxo-phytodienoic acid.

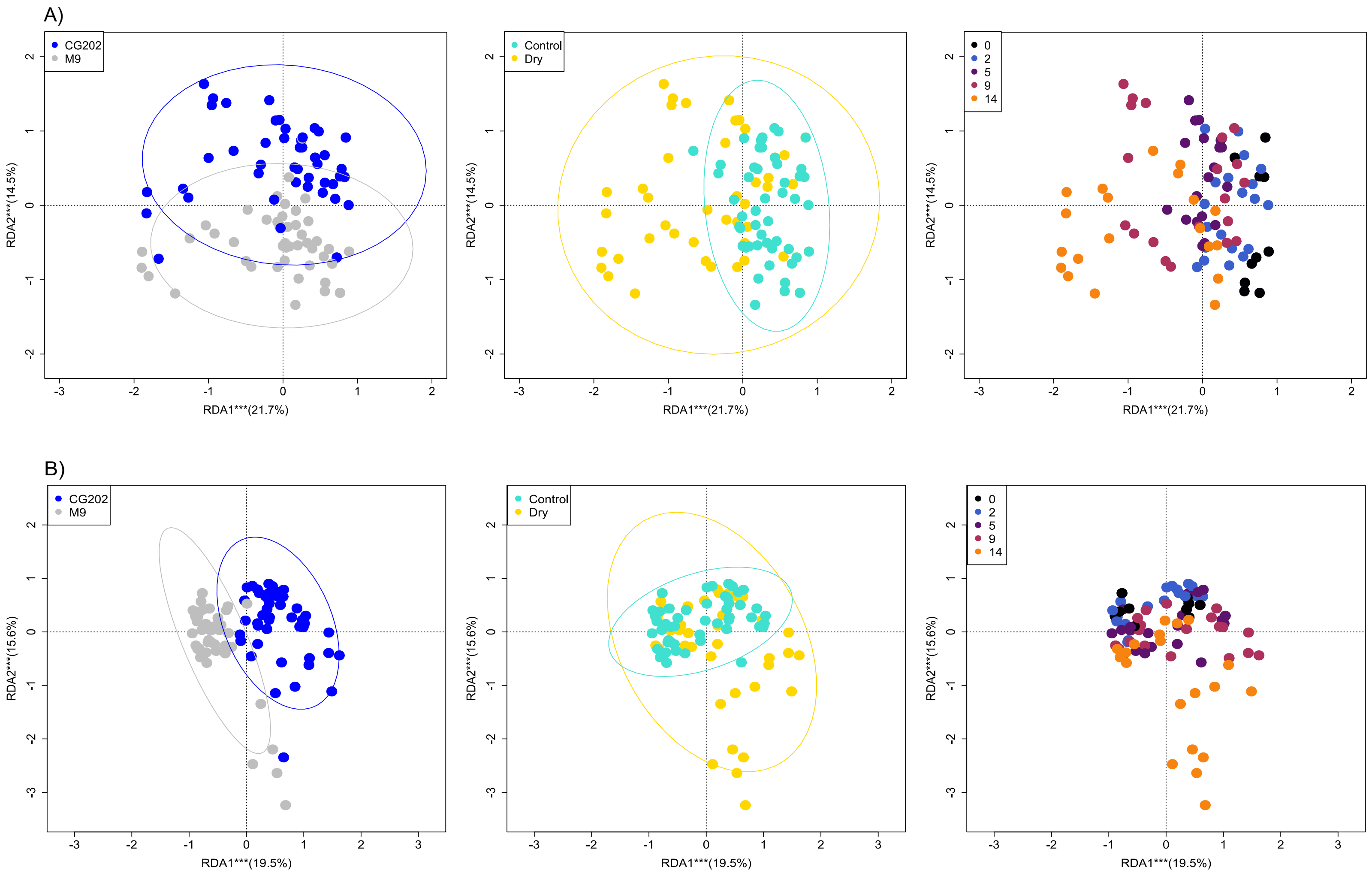

Supplementary Figure S13: Redundancy analysis (RDA) sample plots for **A**) roots and **B**) leaves from two apple rootstock genotypes (CG202, M9), with genotype, treatment (Dry vs Irrigated) and sampling day (0, 2, 5, 9, 14 days) as response variables, in Trial 1. The asterisks indicate that explained variance is significant at *α=0.05, ** α=0.01, *** α=0.001.

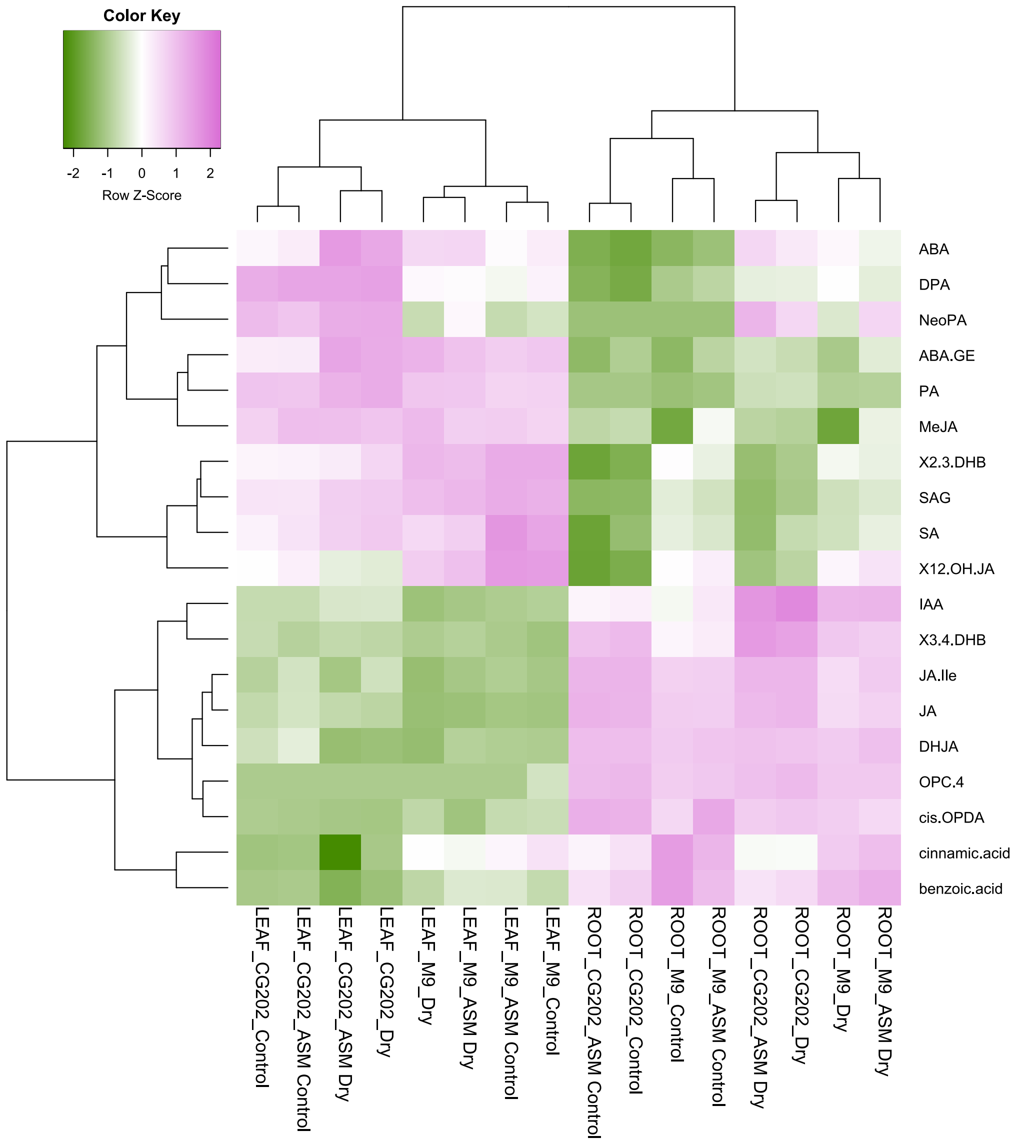

Supplementary Figure S14: Clustered Heatmap of phytohormone concentrations common to leaves and roots of two apple rootstock genotypes (CG202, M9) across common sampling timepoints (day 5, 9, 14) in Trial 2. Dry: drought-treated, Control: Irrigated, ASM: Actiguard-treated. Data are scaled by row with purple hues indicating positive and green hues negative standard (Z-) scores, i.e., relative concentrations. **SA:** salicylic acid; **SAG:** salicylic acid glucoside; **DHB**: dihydroxy benzoic acid; **IAA:** indole-3-acetic acid; **ABA**: abscisic acid; **PA**: phaseic acid; **DPA**: dihydrophaseic acid; **ABA.GE:** abscisic acid glucoside; **NeoPA**: Neophaseic acid; **JA**: jasmonic acid; **JA-Ile**: jasmonic acid-isoleucine; **X12.OH.JA**: 12-hydroxyjasmonic acid; **DHJA**: dihydrojasmonic acid; **OPC-4**: 4-(3-oxo-2-(pent-2-en-1-yl)cyclopentyl)octanoic acid; **cis-OPDA**: 12-oxo-phytodienoic acid; **MeJA**: Methyl Jasmonate.

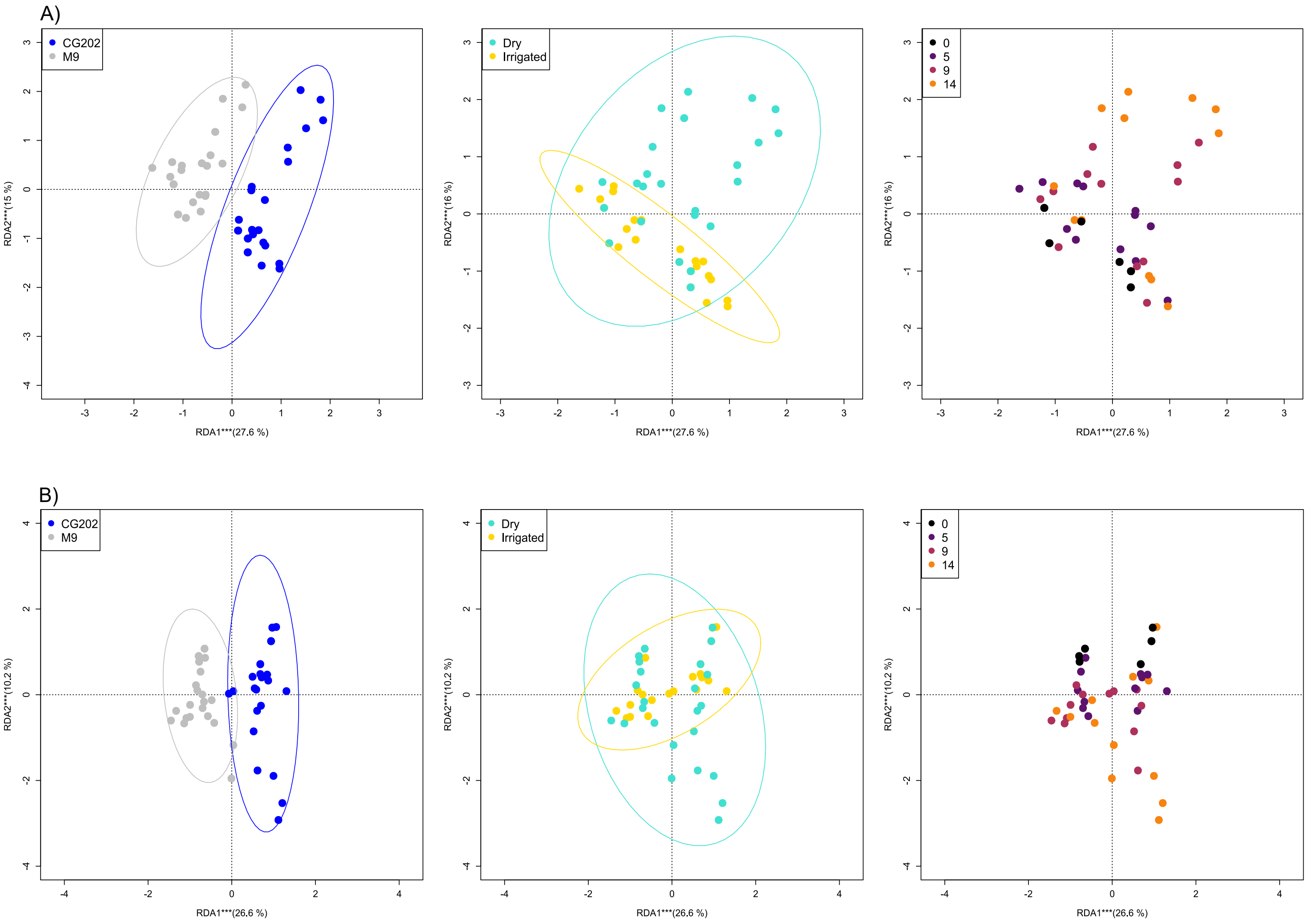

Supplementary Figure S15: Redundancy analysis (RDA) sample plots for **A**) roots and **B**) leaves from two apple rootstock genotypes (CG202, M9), with genotype, treatment (dry vs irrigated) and sampling day (5, 9, 14 days) as response variables, in Trial 2. The asterisks indicate that explained variance is significant at *α=0.05, ** α=0.01, *** α=0.001.

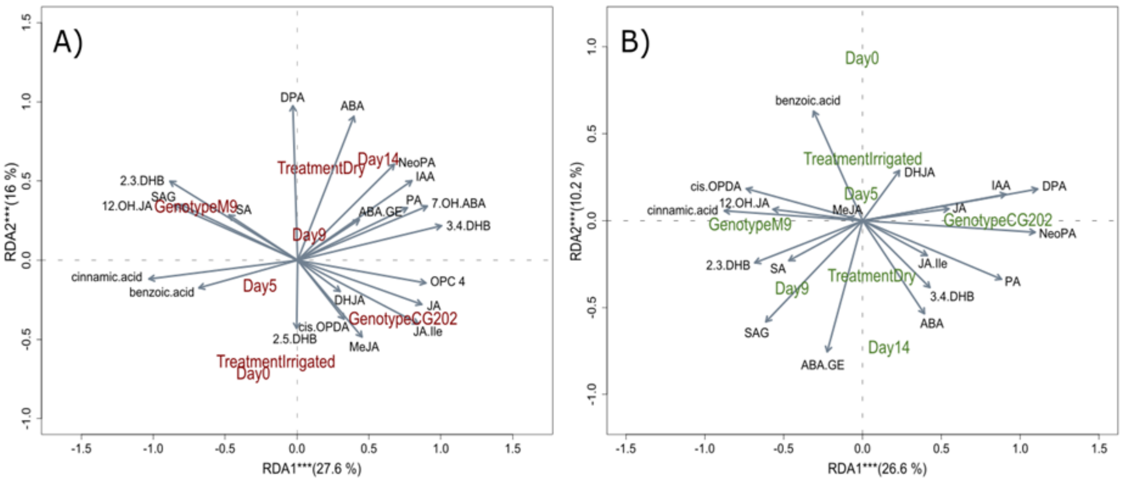

Supplementary Figure S16. Redundancy analysis (RDA) biplots for **A**) roots and **B**) leaves from two apple rootstock genotypes (CG202, M9) with treatment (Dry vs Irrigated), genotype and sampling day as response variables in Trial 2. The vectors visualise variable loadings and sample distributions by group are highlighted in brown (A) and green (B) text for leaves and roots, respectively. The asterisks indicate that explained variance by the RDA components is significant at *α=0.05, ** α=0.01, *** α=0.001. **SA:** salicylic acid; **SAG:** salicylic acid glucoside; **DHB**: dihydroxy benzoic acid; **IAA:** indole-3-acetic acid; **ABA**: abscisic acid; **PA**: phaseic acid; **DPA**: dihydrophaseic acid; **7-OHABA**: 7'-hydroxy-abscisic acid**; ABA-GE:** abscisic acid glucoside; **NeoPA**: neo phaseic acid; **JA**: jasmonic acid; **JA-Ile**: jasmonic acid-isoleucine; **12-OH-JA**: 12-hydroxyjasmonic acid; **DHJA**: dihydrojasmonic acid; **OPC-4**: 4-(3-oxo-2-(pent-2-en-1-yl)cyclopentyl)octanoic acid; **cis-OPDA**: 12-oxo-phytodienoic acid; **MeJA**: methyl jasmonate.

**A) Roots**	**CG202**			**M9**		
	**5**	**9**	**14**	**5**	**9**	**14**
12-OH-JA	1.6	1.0	**2.0**	1.6	1.0	0.8
2.5-DHB	1.1	0.7	0.8	1.6	0.8	**0.5**
ABA	**4.0**	**10.4**	**29.4**	**3.5**	**4.5**	**12.5**
ABA-GE	**2.2**	1.0	0.8	1.5	1.1	1.4
Cinnamic acid	1.0	1.1	**0.3**	0.8	0.6	0.7
cis-OPDA	1.9	**0.4**	**0.4**	**2.0**	2.7	**0.3**
DPA	**2.7**	**6.7**	**7.4**	**2.0**	**3.1**	**6.5**
IAA	1.4	1.6	**3.2**	1.3	1.7	**2.0**
NeoPA	1.0	1.0	**4.8**	1.0	1.0	1.0
PA	2.0	**3.7**	**4.1**	1.5	1.6	**3.1**
SAG	**2.0**	1.3	1.0	0.9	0.7	0.7

**B) Leaves**	**CG202**			**M9**		
	**5**	**9**	**14**	**5**	**9**	**14**
2.3-DHB	**2.3**	0.7	1.9	0.8	0.9	0.9
ABA	1.3	**4.1**	**12.7**	0.7	1.3	**3.7**
ABA-GE	1.1	**2.2**	**3.9**	1.2	1.1	1.5
cis-OPDA	0.9	1.2	0.7	1.1	1.3	**0.4**
JA	1.1	0.7	0.9	1.2	1.2	**0.4**
JA-Ile	1.7	1.5	1.4	0.9	1.8	**0.3**
NeoPA	0.9	0.7	**2.2**	1.0	1.0	1.0
PA	1.4	**2.4**	**4.4**	1.0	1.0	**3.7**
SAG	1.8	0.7	**2.5**	0.7	0.8	0.8

Supplementary Figure S17: Heat map presenting fold-change data of metabolite concentrations in **A)** leaves, and **B)** roots of *Malus domestica* 'GG202' and 'M9' rootstocks after 0, 2, 5, 9, and 14 days without water. All fold changes are relative to irrigated plants at the same time point. Red colouration indicates a fold-increase, blue a fold-decrease, and white is no change relative to the irrigated control. A 2-fold change cutoff was applied for variable selection and bolded data indicate samples with min 2-foldchange difference. **DHB**: dihydroxy benzoic acid; **IAA:** indole-3-acetic acid; **ABA**: abscisic acid; **PA**: phaseic acid; **DPA**: dihydrophaseic acid; **7-OH-ABA**: 7'-hydroxy-abscisic acid**; ABA-GE:** abscisic acid glucoside; **NeoPA**: Neo phaseic acid; **JA**: jasmonic acid; **JA-Ile**: jasmonic acid-isoleucine; **12-OH-JA**: 12-hydroxyjasmonic acid; **DHJA**: dihydrojasmonic acid; **cis-OPDA**: 12-oxo-phytodienoic acid; **MeJA**: Methyl Jasmonate.

Supplementary Table S1: Identification of reference genes (RGs) and genes of interest (GoI) used for gene expression analysis by PlexSet® NanoString of apple rootstock responses to drought and acibenzolar-S-methyl (ASM) application in this study.

Gene Name (Abbreviation)	Identification number[5]	GDDH13 apple reference models [5]	Description, function and key reference(s)
BMS1 ribosome biogenesis factor (*BMS1*)[1]	MDP0000233640	MD00G1071300[6]	RG – GTPase protein BMS1 homolog involved in ribosome biogenesis, [1,2]
Casein kinase II beta chain (*CKB4*)[1]	MDP0000095375	MD02G1221400	RG – casein kinase II beta chain, CK2 regulatory unit involved in circadian clock regulation, [3]
Phytochrome-associated serine/threonine-protein phosphatase 3 (*FYPP3*)[1]	MDP0000060858	MD16G1004700	RG – flower-specific phytochrome-associated protein phosphatase, modulates phytochrome signals in flowering time control, [3]
Glycerol-3-phosphate acyltransferase 1 (*GPAT1*)[1]	MDP0000326399	MD10G1138100	RG – involved in the formulation of acyl lipids (present in membranes, lipid storage forms), [3]
Li-tolerant lipase 1 (*LTL1*)[1]	MDP0000173025	MD16G1209000	RG – StaR-related lipid transport like protein, [3]
GRIP-domain protein (*Protein GRIP*)[1]	MDP0000147424	MD15G1233300	RG – GRIP-domain golgi trafficking protein, [1,2]
Abscisic acid-insensitive 4, isoform i (*ABI4_i*)[3]	MD01G0125200	MD01G1155400	GoI – ABI4 ortholog of the ERF/AP2 TF family, regulates the ABA dependent stress response, [4,5]
ABI4, isoform ii (*ABI4_ii*)[3]	MD07G0184900	MD07G1224400	GoI – same as *ABI4_i*, [4,5]
Arabidopsis MADS-box factor, isoform i (*AGL16_i*)[4]	MD08G0170000	MD08G1206400	GoI – MADS-box protein, isoform i, based on *Arabidopsis thaliana* data for its ortholog At3g57230, a negative regulator of salt stress, [6]
AGL16, isoform ii (*AGL16_ii*)[4]	MD15G0339300	MD15G1389800	GoI – same as for *AGL16_i*, [6]
Auxin response factor 17 (*ARF17*)[3]	XM_008340583.3 (MDP0000232116 MD15G0307900)	MD15G1359400	GoI – involved in auxin responses & a negative regulator of adventitious rooting, downregulated under drought stress, [7,8]
Autophagy-related protein 18 (*ATG18*)[4]	KC800804.1 MD11G0248600	MD11G1303400	GoI – autophagy gene which prevents premature cleavage during phagophore assembly & alleviates oxidative damage in drought, [9]
Basic helix-loop-helix protein 130 (*Md bHLH130*)[4]	MDP0000581816 MD03G0019800	MD03G1023200	GoI – ABA-responsive transcription factor (TF), positive regulator of stomatal closure and ROS scavenging, [10-12]

Brassinazole resistant 1/BRI1-EMS suppressor 1 (*BZR2/BES1*)[4]	MD08G0030000	MD08G1035400	GoI – positively regulates brassinosteroid signalling action, acting as a hub to coordinate growth and stress responses, [13,14]
Calcium sensing receptor (*CAS*)[3]	XM_008362669.3<br>MD14G0190100,<br>MDP0000236317	MD14G1219000	GoI – calcium receptor, based on *A. thaliana* data for its ortholog, At5g23060, influences water use efficiency under drought stress, [15]
Eceriferum 2, isoform i (*CER2_i*) [3]	MD15G0330500<br>MDP0000275850	MD15G1386800	GoI – promotes cuticular wax accumulation, decreases water permeability, increases lateral root growth, & changes ABA sensitivity, providing water-loss protection, [16]
CER2, isoform ii (*CER2_ii*)[3]	MD04G0015000<br>MDP0000698860	MD04G1016900	GoI – same as for *CER2_i*, [16]
Cytochrome P450-707A1, isoform i (*CYP707A1_i*)[3]	MD16G0240600	MD16G1285900	GoI – ABA 8'-hydroxylase 1, catabolises ABA to inert PA, [17]
CYP450-707A1, isoform ii (*CYP707A1_ii*)[3]	MD05G0067900	MD05G1081400	GoI – same as for *CYP707A1_i*, [17]
Cytochrome P450-707A2, (*CYP707A2*)[3]	XM_008358695.3<br>MDP0000326412<br>MD03G0072500	MD03G1088100	GoI – encodes a different ABA 8'-hydroxylase family member to CYP707A1, [17]
Dehydration-responsive element binding factor 2C, isoform i (*DREB2C*)[3]	XM_008355947.3<br>MD01G0128100	MD01G1158600	GoI – DREB2C ortholog of the ERF/AP2 TF family, regulates the ABA dependent stress response, [4,5]
Early Response to Dehydration 15, isoform 1 (*ERD15_i*)[2]	MDP0000557979<br>MD12G0070300	MD12G1089300[7]	GoI – modulates ABA-induced gene expression, especially in response to drought, [7]
ERD15, isoform ii (ERD15_ii)[3]	MDP0000557979<br>MD14G0071500	MD14G1081500	GoI – same as for *ERD15_i*, [7]
Ethylene responsive transcription factor, isoform i (*ERF053-i*)[3]	MD09G0216600.1	MD09G1252000	GoI – stress responsive, drought-induced TF family, [18]
ERF053, isoform ii (*ERF053_ii*)[3]	MD17G0208100.1	MD17G1244300	GoI – same as for ERF053_i, [18]
FK506-binding protein 65a (*FKBP65a*)[3]	MDP0000175388<br>MD04G0020200	MD04G1022400	GoI – involved in water-deficit signalling, [19]
FK506-binding protein 62a (*FKBP62a*)[2]	MDP0000141863<br>MD05G0136800	MD05G1166200	GoI – involved in water-deficit signalling, [19]

Hypocotyl 5 (HY5)[2]	MD08G0124100	MD08G1147100	GoI – basic-leucine zipper (bZIP) TF that mediates ABA responses, based on Arabidopsis data, [20]
Myeloblastosis 68 (MYB68)[3]	MDP0000836365 MD15G0175700	MD15G1201500	GoI – MYB-domain TF, negative regulator for root development, and down-regulated by drought, [7,21]
Myeloblastosis 88/ myeloblastosis 124 (MYB88/ MYB124)[3]	MDP0000210970 MD13G0066400	MD13G1075000	GoI – MYB-domain TF critical for ABA accumulation in apple following drought, regulation negatively controlled by ABA, [22]
NAC-domain transcription factor 1 (Md NAC1)[4]	MF401514.1 MDP0000240094 MD15G0352200	MD15G1415700	GoI – NAC/ANAC TF, enhances drought tolerance in apple, [23]
NAC-domain transcription factor 143 (Md NAC143)[4]	MDP0000334047 MD15G0119800	MD15G1136600	GoI – NAC/ANAC TF, responds to stress and interacts with negative regulator drought stress, homologous to AtNAC002, [24,25]
Nine-cis-epoxycarotenoid dioxygenase 3, isoform i (NCED3_i)[3]	MD05G0237600	MD05G1282700	GoI – critical enzyme in ABA synthesis, especially under dehydration stress, [4,26]
NCED3, isoform ii (NCED3_ii)[3]	MD10G0218700	MD10G1261000	GoI – same as for *NCED3_i*, [4,26]
NCED3, isoform iii (NCED3_iii)[3]	XM_008384748.3 MD10G0164700.1	MD10G1194200	GoI – same as for *NCED3_i*, [4,26]
Pyrabactin resistance1 like regulatory components of ABA receptor 4, isoform i (PYL4_i)[3]	MDP0000228470 MD04G0131100	MD04G1165000	GoI – ABA-receptor important in initiation of ABA signalling, responsive to drought stress, [18,27]
PYL4, isoform ii (PYL4_ii)[3]	MD12G0147400	MD12G1178800	GoI – same as for *PYL4_i*, [18,27]
PYL4, isoform iii (PYL4-iii)[3]	MD01G0128000	MD01G1158500	GoI – same as for *PYL4_i*, [18,27]
PYL4, isoform iv (PYL4-iv)[3]	MD07G0187200	MD07G1227100	GoI – same as for *PYL4_i*, [18,27]
Pyrabactin resistance1 like regulatory components of ABA receptor 9, isoform i (PYL9_i)[2]	MD07G0121300	DD07G1147700	GoI – another ABA-receptor family important in initiation of ABA signalling, responsive to drought stress, [18,27]

PYL9, isoform ii (*PYL9_ii*)[2]	MD01G0060400 MDP0000284624	MD01G1078900	GoI – same as for *PYL9_i*, [18,27]
PYL9, isoform iii (*PYL9_iii*)[2]	MD01G0178700	MD01G1216100	GoI – same as for *PYL9_i*, [18,27]
Ethylene-responsive factor RAP2.4, isoform i (*RAP2.4_i*)[3]	MDP0000865032 MD01G0016100.1	Match 5'& 3' UTRs and ORF of MD01G1027200	GoI – Ethylene responsive TF/AP2 domain protein, upregulated under drought stress, [7,28]
RAP 2.4, isoform ii (*RAP2.4_ii*)[2]	MD08G0148800	MD08G1180200	GoI – same as for *RAP2.4_i*, [7,28]
Responsive to desiccation 29B, isoform i (*RD29B_i*)[3]	MD07G0224300	MD07G1268800	GoI – controls differential expression of drought-related TFs and genes, [4,29,30]
RD29B, isoform ii (*RD29B_ii*)[3]	MD01G0164400	MD01G1201000	GoI – same as for *RD29B_i*, [4,29,30]
Serine acyl transferase 1 (*SAT1*)[2]	XM_008365876.4 MDP0000123573 MD03G0152800	MD03G1187100	GoI – chloroplast enzyme regulating final step in cysteine biosynthesis. SAT1 refolding by an OPDA binding cyclophilin that is modulated by ABA and regulated by light and redox signals linking to stress response via glutathione. Mitigates ROS production during abiotic stress. Arabidopsis evidence, [31,32]
Sorbitol dehydrogenase1 (*SDH1*)[4]	MD01G0086500	MD01G1110100	GoI – Involved in sugar metabolism and possible osmostic regulation in response to water deficiency, [33], note found by matching qPCR primers only
SERRATE (*SERRATE*)[2]	MD11G0146500	MD11G1180900	GoI – RNA processing Zinc Finger protein, that negatively regulates drought. The SERRATE in the publication (MD00G1031700) is most likely a homoeolog to the gene used here, but both are 94% identical at the protein level, [34]
Sucrose non-forming (SNF)-related kinase family 2.3 (*SnRK2.3*)[4]	MDP0000224969 MD02G0143100	MD02G1166500	GoI – ABA-dependent serine/threonine kinases regulating the plant response to abiotic stresses, [18,27]
SNF-related kinase family 2.6 (*SnRK2.6/OST1*)[4]	MD10g0073900	MD10G1089100	GoI – ABA-dependent serine/threonine kinases regulating the plant response to abiotic stresses. Nearest match to OST1 [18,27,30]

SNF-related kinase family 2.8 (*SnRK2.8*)[4]	MD15G0318600	MD15G1373000	GoI – ABA-dependent serine/threonine kinases regulating the plant response to abiotic stresses, [18,27]
Tonoplast instrinsic protein 1;1 (*TIP1;1*)[3]	JF834207<br>MDP0000561026<br>MD12G0086700	MD12G1107400[8]	GoI – membrane aquaporin (water channel protein) differentially expressed in drought tolerant & sensitive cultivars, [35]
Tonoplast instrinsic protein 4;1 (*TIP4;1*)[3]	MDP0000271305<br>MDP0000278588<br>MD15G0082000	MD15G1094400	GoI – aquaporin TIP4s are reported to express in epidermal and cortical cells of the differentiation zone of Arabidopsis roots, [7,36]
Vacuolar proton (H^+) *ATPase B1 unit, isoform i* (*VHA-B_i*)[2]	NM106251.4<br>MDP0000945182<br>MD10G0104600	MD10G1127400	GoI – interact with actin cytoskeletons and E3 ligases linked to ABA signal modulation by endomembrane trafficking, [37]
VHA-B, isoform ii (*VHA-B_ii*)[2]	MDP0000631168<br>MD05G0102900	MD05G1124700	GoI – same as for *VHA-B_i*, [37]
Wrinkled 4, isoform i (WRI4_i)[3]	LOC103444002<br>MDP0000166059<br>MD09G0197300	MD09G1228500	GoI – An AP2/ERF TF that controls wax synthesis, increasing cuticular wax load and enhancing resistance to drought in apple, [38]
WRI4, isoform ii (*WRI4_ii*)[3]	MD16G0219300	MD16G1258700	GoI – same as for *WRI4_i*, [38]
WRKY DNA-binding protein 40 (*WRKY40*)[3]	XM_008344585.3<br>MDP0000177906<br>MD17G0190600	MD17G1223100	GoI – biotic response TF with WRKYs 18 and 60, but a also negative regulator of cysteine-rich receptor-like protein kinase AtCRK5, which confers drought tolerance in Arabidopsis [7,39]

[1]Reference gene set used in both trials, derived from the reference gene sets identified by Bowen et al. [3] and Velasco et al. [1].

[2]Used in Trial 1 only – not used in Trial 2 because these genes did not show any significant differential changes in expression.

[3]Used in both Trial 1 and Trial 2.

[4]Genes newly introduced in Trial 2.

[5]MDP numbers refer to the original gene calls from contigs of the Golden Delicious genome of Velasco et al [1] before they were placed into fully ordered chromosomes. Matches to these gene calls (M. x domestica v1.0 genes) are also placed as a track on the GDDH13 v1.1 genome in JBrowse. The MD numbers refer to the original ordering and re-annotated gene calls in the Golden Delicious genome after fully ordering them on chromosomes, the originals are given as they are used in EnsemblePlants as the names of *Malus* genes and are distinguishable from the re-annotated GDDH13 gene

names by having a 0 as the first digit after the chromosome ID in the name. GDDH13 gene names have a 1 as the first digit after the chromosome ID in the name. Original GD-ASM apple models in blue, current GD-ASM apple models in black, accession numbers in pink.

[6]Note that this gene has not been place on a chromosome in GDDH13, the closest matching gene placed in GDDH13 (likely a homoeolog) is MD03G1154000.

[7]Note that in the GDDH13 database the annotation of this gene matches an alternative transcript in the same region that reads regions equivalent to an intron in the original version of the genome. That alternative version does not match the open reading frames deduced in the homoeologous version in LG14 which suggests this GDDH13 gene may be an erroneous annotation, and the original annotation may have been correct.

[8]Note an alternative transcript that runs in the reverse direction is annotated from this locus in GDDH13. Protein reads from the original annotation match gene models in apple and Arabidopsis. Conversely, protein reads from the re-annotation do not have any reasonable BlastP gene model matches in apple or Arabidopsis, nor any reasonable tBlastn matches to 19 Arabidopsis genomes, implying this is likely an erroneous re-annotation.

Table References

1. Velasco, R.; Zharkikh, A.; Affourtit, J.; Dhingra, A.; Cestaro, A.; Kalyanaraman, A.; Fontana, P.; Bhatnagar, S.K.; Troggio, M.; Pruss, D.*, et al*. The genome of the domesticated apple (*Malus domestica* Borkh.). *Nature Genetics* **2010**, *42*, 833-+, doi:10.1038/ng.654.
2. Zhou, Z.; Cong, P.; Tian, Y.; Zhu, Y. Using RNA-seq data to select reference genes for normalizing gene expression in apple roots. *PLoS One* **2017**, *12*, 17, doi:10.1371/journal.pone.0185288.
3. Bowen, J.; Ireland, H.; Crowhurst, R.; Luo, Z.; Watson, A.; Foster, T.; Gapper, N.; Giovanonni, J.; Mattheis, J.; Watkins, C.*, et al*. Selection of low-variance expressed *Malus domestica* (apple) genes for use as quantitative PCR reference genes (housekeepers). *Tree Genet. Genomes* **2014**, *10*, 751-759, doi:10.1007/s11295-014-0720-6.
4. Chen, K.; Guo, Y.; Song, M.; Liu, L.; Xue, H.; Dai, H.; Zhang, Z. Dual role of MdSND1 in the biosynthesis of lignin and in signal transduction in response to salt and osmotic stress in apple. *Hortic. Res.-England* **2020**, *7*, doi:10.1038/s41438-020-00433-7.
5. Li, X.; Xie, Y.; Lu, L.; Yan, M.; Fang, N.; Xu, J.; Wang, L.; Yan, Y.; Zhao, T.; van Nocker, S.*, et al*. Contribution of methylation regulation of *MpDREB2A* promoter to drought resistance of *Malus prunifolia*. *Plant and Soil* **2019**, *441*, 15-32, doi:10.1007/s11104-019-04149-z.
6. Zhao, P.-X.; Zhang, J.; Chen, S.-Y.; Wu, J.; Xia, J.-Q.; Sun, L.-Q.; Ma, S.-S.; Xiang, C.-B. Arabidopsis MADS-box factor AGL16 is a negative regulator of plant response to salt stress by downregulating salt-responsive genes. *New Phytol.* **2021**, *232*, 2418-2439, doi:10.1111/nph.17760.
7. Da-li, G.; Li-yuan, L.; Ming-jia, Y.; Xiao-xia, S.; Li-juan, J.; Hai-yan, L.; Li-ping, W.; Yan, Y.; Ji-di, X.; Cui-ying, L.*, et al*. Physiological and transcriptomic analyses of roots from *Malus sieversii* under drought stress. *Journal of Integrative Agriculture* **2019**, *18*, 1280-1294, doi:10.1016/s2095-3119(19)62571-2.

8. Gutierrez, L.; Mongelard, G.; Flokova, K.; Pacurar, D.I.; Novak, O.; Staswick, P.; Kowalczyk, M.; Pacurar, M.; Demailly, H.; Geiss, G., *et al*. Auxin Controls <i>Arabidopsis</i> Adventitious Root Initiation by Regulating Jasmonic Acid Homeostasis. *Plant Cell* **2012**, *24*, 2515-2527, doi:10.1105/tpc.112.099119.
9. Sun, X.; Wang, P.; Jia, X.; Huo, L.; Che, R.; Ma, F. Improvement of drought tolerance by overexpressing *MdATG18a* is mediated by modified antioxidant system and activated autophagy in transgenic apple. *Plant Biotechnology Journal* **2018**, *16*, 545-557, doi:10.1111/pbi.12794.
10. Li, X.; Ma, Z.; Song, Y.; Shen, W.; Yue, Q.; Khan, A.; Tahir, M.; Wang, X.; Malnoy, M.; Ma, F., *et al*. Insights into the molecular mechanisms underlying responses of apple trees to abiotic stresses. *Hortic. Res.-England* **2023**, *10*, 14, doi:10.1093/hr/uhad144.
11. Takahashi, Y.; Ebisu, Y.; Shimazaki, K.-i. Reconstitution of Abscisic Acid Signaling from the Receptor to DNA via bHLH Transcription Factors. *Plant Physiology* **2017**, *174*, 815-822, doi:10.1104/pp.16.01825.
12. Zhao, Q.; Fan, Z.; Qiu, L.; Che, Q.; Wang, T.; Li, Y.; Wang, Y. <i>MdbHLH130</i>, an Apple bHLH Transcription Factor, Confers Water Stress Resistance by Regulating Stomatal Closure and ROS Homeostasis in Transgenic Tobacco. *Front. Plant Sci.* **2020**, *11*, doi:10.3389/fpls.2020.543696.
13. Jiang, S.; Li, S.; Liu, X.; Wen, B.; Wang, N.; Zhang, R.; Li, D.; Chen, X.; Fu, X.; Xiao, W., *et al*. Genome-wide identification and characterization of the <i>MdBZR1</i> gene family in apple and their roles in improvement of drought tolerance. *Sci. Hortic.* **2021**, *288*, doi:10.1016/j.scienta.2021.110359.
14. Kono, A.; Yin, Y. Updates on BES1/BZR1 Regulatory Networks Coordinating Plant Growth and Stress Responses. *Front. Plant Sci.* **2020**, *11*, doi:10.3389/fpls.2020.617162.
15. Wang, W.-H.; Chen, J.; Liu, T.-W.; Chen, J.; Han, A.-D.; Simon, M.; Dong, X.-J.; He, J.-X.; Zheng, H.-L. Regulation of the calcium-sensing receptor in both stomatal movement and photosynthetic electron transport is crucial for water use efficiency and drought tolerance in *Arabidopsis*. *Journal of Experimental Botany* **2014**, *65*, 223-234, doi:10.1093/jxb/ert362.
16. Zhong, M.-S.; Jiang, H.; Cao, Y.; Wang, Y.-X.; You, C.-X.; Li, Y.-Y.; Hao, Y.-J. <i>MdCER2</i> conferred to wax accumulation and increased drought tolerance in plants. *Plant Physiology and Biochemistry* **2020**, *149*, 277-285, doi:10.1016/j.plaphy.2020.02.013.
17. Kondo, S.; Sugaya, S.; Sugawa, S.; Ninomiya, M.; Kittikorn, M.; Okawa, K.; Ohara, H.; Ueno, K.; Todoroki, Y.; Mizutani, M., *et al*. Dehydration tolerance in apple seedlings is affected by an inhibitor of ABA 8'-hydroxylase CYP707A. *J. Plant Physiol.* **2012**, *169*, 234-241, doi:10.1016/j.jplph.2011.09.007.
18. Tan, Y.; Li, M.; Yang, Y.; Sun, X.; Wang, N.; Liang, B.; Ma, F. Overexpression of <i>MpCYS4</i>, A Phytocystatin Gene from <i>Malus prunifolia</i> (Willd.) Borkh., Enhances Stomatal Closure to Confer Drought Tolerance in Transgenic *Arabidopsis* and Apple. *Front. Plant Sci.* **2017**, *8*, doi:10.3389/fpls.2017.00033.
19. Dong, Q.; Mao, K.; Duan, D.; Zhao, S.; Wang, Y.; Wang, Q.; Huang, D.; Li, C.; Liu, C.; Gong, X., *et al*. Genome-wide analyses of genes encoding FK506-binding proteins reveal their involvement in abiotic stress responses in apple. *Bmc Genomics* **2018**, *19*, doi:10.1186/s12864-018-5097-8.

20. Chen, H.; Zhang, J.; Neff, M.M.; Hong, S.-W.; Zhang, H.; Deng, X.-W.; Xiong, L. Integration of light and abscisic acid signaling during seed germination and early seedling development. *Proceedings of the National Academy of Sciences of the United States of America* **2008**, *105*, 4495-4500, doi:10.1073/pnas.0710778105.
21. Feng, C.; Andreasson, E.; Maslak, A.; Mock, H.; Mattsson, O.; Mundy, J. *Arabidopsis MYB68* in development and responses to environmental cues. *Plant Sci.* **2004**, *167*, 1099-1107, doi:10.1016/j.plantsci.2004.06.014.
22. Xie, Y.; Bao, C.; Chen, P.; Cao, F.; Liu, X.; Geng, D.; Li, Z.; Li, X.; Hou, N.; Zhi, F., *et al.* Abscisic acid homeostasis is mediated by feedback regulation of *MdMYB88* and *MdMYB124*. *Journal of Experimental Botany* **2021**, *72*, 592-607, doi:10.1093/jxb/eraa449.
23. Jia, D.; Jiang, Q.; van Nocker, S.; Gong, X.; Ma, F. An apple (*Malus domestica*) NAC transcription factor enhances drought tolerance in transgenic apple plants. *Plant Physiology and Biochemistry* **2019**, *139*, 504-512, doi:10.1016/j.plaphy.2019.04.011.
24. Ji, X.L.; Li, H.L.; Qiao, Z.W.; Zhang, J.C.; Sun, W.J.; Wang, C.K.; Yang, K.; You, C.X.; Hao, Y.J. The BTB-TAZ protein MdBT2 negatively regulates the drought stress response by interacting with the transcription factor MdNAC143 in apple. *Plant Sci.* **2020**, *301*, 10, doi:10.1016/j.plantsci.2020.110689.
25. Su, H.; Zhang, S.; Yuan, X.; Chen, C.; Wang, X.-F.; Hao, Y.-J. Genome-wide analysis and identification of stress-responsive genes of the NAM-ATAF1,2-CUC2 transcription factor family in apple. *Plant Physiology and Biochemistry* **2013**, *71*, 11-21, doi:10.1016/j.plaphy.2013.06.022.
26. Endo, A.; Sawada, Y.; Takahashi, H.; Okamoto, M.; Ikegami, K.; Koiwai, H.; Seo, M.; Toyomasu, T.; Mitsuhashi, W.; Shinozaki, K., *et al.* Drought induction of Arabidopsis 9-cis-epoxycarotenoid dioxygenase occurs in vascular parenchyma cells. *Plant Physiology* **2008**, *147*, 1984-1993, doi:10.1104/pp.108.116632.
27. Guo, T.; Wang, N.; Xue, Y.; Guan, Q.; van Nocker, S.; Liu, C.; Ma, F. Overexpression of the RNA binding protein MhYTP1 in transgenic apple enhances drought tolerance and WUE by improving ABA level under drought condition. *Plant Sci.* **2019**, *280*, 397-407, doi:10.1016/j.plantsci.2018.11.018.
28. Lin, R.-C.; Park, H.-J.; Wang, H.-Y. Role of *Arabidopsis* RAP2.4 in regulating light- and ethylene-mediated developmental processes and drought stress tolerance. *Molecular Plant* **2008**, *1*, 42-57, doi:10.1093/mp/ssm004.
29. Liu, W.; Thapa, P.; Park, S.-W. *RD29A* and *RD29B* rearrange genetic and epigenetic markers in priming systemic defense responses against drought and salinity. *Plant Sci.* **2023**, *337*, doi:10.1016/j.plantsci.2023.111895.
30. Shao, Y.; Zhang, X.; van Nocker, S.; Gong, X.; Ma, F. Overexpression of a protein kinase gene *MpSnRK2.10* from *Malus prunifolia* confers tolerance to drought stress in transgenic *Arabidopsis thaliana* and apple. *Gene* **2019**, *692*, 26-34, doi:10.1016/j.gene.2018.12.070.
31. Dominguez-Solis, J.R.; He, Z.; Lima, A.; Ting, J.; Buchanan, B.B.; Luan, S. A cyclophilin links redox and light signals to cysteine biosynthesis and stress responses in chloroplasts. *Proceedings of the National Academy of Sciences of the United States of America* **2008**, *105*, 16386-16391, doi:10.1073/pnas.0808204105.
32. Park, S.-W.; Li, W.; Viehhauser, A.; He, B.; Kim, S.; Nilsson, A.K.; Andersson, M.X.; Kittle, J.D.; Ambavaram, M.M.R.; Luan, S., *et al.* Cyclophilin 20-3 relays a 12-oxo-phytodienoic acid signal during stress responsive regulation of cellular redox homeostasis. *Proceedings of the National Academy of Sciences of the United States of America* **2013**, *110*, 9559-9564, doi:10.1073/pnas.1218872110.

33. Yang, J.; Zhang, J.; Li, C.; Zhang, Z.; Ma, F.; Li, M. Response of sugar metabolism in apple leaves subjected to short-term drought stress. *Plant Physiology and Biochemistry* **2019**, *141*, 164-171, doi:10.1016/j.plaphy.2019.05.025.
34. Li, X.; Chen, P.; Xie, Y.; Yan, Y.; Wang, L.; Dang, H.; Zhang, J.; Xu, L.; Ma, F.; Guan, Q. Apple SERRATE negatively mediates drought resistance by regulating MdMYB88 and MdMYB124 and microRNA biogenesis. *Hortic. Res.-England* **2020**, *7*, 98-98, doi:10.1038/s41438-020-0320-6.
35. Liu, C.; Li, C.; Liang, D.; Ma, F.; Wang, S.; Wang, P.; Wang, R. Aquaporin expression in response to water-deficit stress in two <i>Malus</i> species: relationship with physiological status and drought tolerance. *Plant Growth Regulation* **2013**, *70*, 187-197, doi:10.1007/s10725-013-9791-x.
36. Gattolin, S.; Sorieul, M.; Hunter, P.R.; Khonsari, R.H.; Frigerio, L. In vivo imaging of the tonoplast intrinsic protein family in Arabidopsis roots. *Bmc Plant Biology* **2009**, *9*, doi:10.1186/1471-2229-9-133.
37. Hu, D.-G.; Wang, S.-H.; Luo, H.; Ma, Q.-J.; Yao, Y.-X.; You, C.-X.; Hao, Y.-J. Overexpression of *MdVHA/B*, a V/ATPase gene from apple, confers tolerance to drought in transgenic tomato. *Sci. Hortic.* **2012**, *145*, 94-101, doi:10.1016/j.scienta.2012.08.010.
38. Zhang, C.-L.; Wang, Y.-X.; Hu, X.; Zhang, Y.-L.; Wang, G.-L.; You, C.-X.; Li, Y.-Y.; Hao, Y.-J. An apple AP2/EREBP-type transcription factor, *MdWRI4*, enhances plant resistance to abiotic stress by increasing cuticular wax load. *Environmental and Experimental Botany* **2020**, *180*, doi:10.1016/j.envexpbot.2020.104206.
39. Lu, K.; Liang, S.; Wu, Z.; Bi, C.; Yu, Y.-T.; Wang, X.-F.; Zhang, D.-P. Overexpression of an Arabidopsis cysteine-rich receptor-like protein kinase, CRK5, enhances abscisic acid sensitivity and confers drought tolerance. *Journal of Experimental Botany* **2016**, *67*, 5009-5027, doi:10.1093/jxb/erw266.
